# Core Competencies of an Anti-racist Physician: Elective Course for Undergraduate Medical Students

**DOI:** 10.15766/mep_2374-8265.11395

**Published:** 2024-05-14

**Authors:** J. Corey Williams, Zharia Crisp, Brendan Crow, Aaron Alexander-Bloch, Katie Galvin, Zheala Qayyum, Jaya Aysola, Susan M. Cheng

**Affiliations:** 1 Assistant Professor, Department of Psychiatry, MedStar Georgetown University Hospital; 2 Second-Year Medical Student, University of Chicago Pritzker School of Medicine; 3 Third-Year Resident Physician, Mountain Area Health Education Center (MAHEC) Family Medicine Residency; 4 Assistant Professor, Department of Child and Adolescent Psychiatry and Behavioral Sciences, The Children's Hospital of Philadelphia and Perelman School of Medicine at the University of Pennsylvania; 5 Assistant Director of Longitudinal Curricular Coordination, Georgetown University School of Medicine; 6 Assistant Professor of Psychiatry, Harvard Medical School; 7 Associate Professor of Medicine, Associate Professor of Pediatrics, and Executive Director, Penn Medicine Center for Health Equity Advancement; 8 Associate Professor, Department of Family Medicine, and Senior Associate Dean for Diversity, Equity, Inclusion & Belonging, Georgetown University School of Medicine

**Keywords:** Case-Based Learning, Problem-Based Learning, Self-Assessment, Anti-racism, Diversity, Equity, Inclusion

## Abstract

**Introduction:**

Medical schools seeking to correct and reform curricula towards anti-racist perspectives need to address anti-Black forms of racism specifically and teach students critical upstander skills to interrupt manifestations of racism. We developed a course to teach preclinical medical students basic anti-racism competencies including recognition and awareness of anti-Black racism in medicine and upstander skills to advocate for patients and colleagues.

**Methods:**

In 2021 and 2022, we designed, implemented, and evaluated an elective course for second-year medical students (*N* = 149) to introduce competencies of anti-racism focusing on upstander skills for addressing anti-Blackness. We designed three patient cases and one student-centered case to illustrate manifestations of anti-Black racism in medicine and used these cases to stimulate small-group discussions and guide students toward recognizing and understanding ways of responding to racism. We designed pre- and postassessments to evaluate the effectiveness of the course and utilized anonymous feedback surveys.

**Results:**

Participants showed significant improvement in pre- to postassessment scores in both years of the course. The anonymous feedback survey showed that 97% of students rated the course at least somewhat effective, and the qualitative responses revealed five core themes: course timing, case complexity, learner differentiation, direct instruction, and access to resources.

**Discussion:**

This course reinforces upstander competencies necessary for advancing anti-racism in medicine. It addresses a gap in medical education by reckoning with the entrenched nature of anti-Black racism in the culture of medicine and seeks to empower undergraduate medical students to advocate for Black-identifying patients and colleagues.

## Educational Objectives

By the end of this activity, students will be able to:

1. Describe multiple ways in which anti-Black racism from physicians negatively influences a clinical encounter.
2. Examine the ways in which legacies of racialized historical violence and structurally racist policies influence various present-day health care encounters.
3. Recognize opportunities to advocate and be an upstander for the Black patients and colleagues within the health care system.
4. Reflect on upstander and advocacy strategies for interrupting racism in a clinical setting.
5. Understand the importance of proactive self-care strategies when engaging in advocacy by being aware of institutional supports and mentorship at multiple levels.

## Introduction

Recent civic unrest and calls for racial justice have amplified the national conversation about the need for anti-racism education in medicine. A proliferation of medical education literature reflects a growing awareness of the entrenched misunderstandings about race in medical education. A recent multi-institutional study of undergraduate medical curricula demonstrated that race is often not contextualized as a social and political construct; rather, race is given false biological importance, thereby reinforcing false biological beliefs about racial differences.^1^ In addition, student advocacy has increased the desire and demand for more contextualized education on race that advances students’ understanding of social determinants of health and the impacts of structural racism. Leaders in medical education are beginning to understand that medical education must correct these missteps, beyond teaching about implicit bias, towards teaching specific anti-racist competencies (i.e., knowledge, skills, and attitudes) for undergraduate medical students.^2^

Not only do medical students need to understand the toll of structural racism broadly—for example, some estimates show over 70,000 excess Black deaths occur in the US annually that are attributable to health inequalities^3^—but they also need to understand how anti-Black racism (a particular form of racism that devalues people of African descent specifically^4^) shapes the culture of medicine. While all forms of bias are critical, students need to recognize that anti-Black forms of racism are a defining aspect of American medical institutions and that well-meaning health care providers participate in anti-Black racism within clinical contexts. Furthermore, evidence suggests that students’ preexisting anti-Black biases are exacerbated by cultural exposure in medical school over time: In one study, 49% of US medical students surveyed reported having been exposed to negative comments about Black patients by attending or resident physicians, and those students demonstrated significantly greater implicit racial bias in year four when compared to year one.^5^ Therefore, undergraduate medical curricula must be targeted directly at disrupting the transfer and acquisition of negative racial attitudes that occur in medicine. Medical educators need curriculum resources that are aimed at describing and teaching students competencies on issues related to anti-racism, social justice, and health equity, ultimately equipping the next generation of physicians with the tools and frameworks necessary to tackle structural racism.

Despite this need, there is a dearth of published curricula that both engage undergraduate medical learners and address the specific forms of anti-Black racism that pervade medical culture. Upon review of the anti-racism collection in *MedEdPORTAL,* we found a number of published curricula for undergraduate medical students that confront the realities of racism in clinical contexts^6–11^—several focusing on social determinants of health, microaggression, bias, and stereotypes—but only one that focuses on anti-Black racism.^9^ To date, no curriculum that focuses on anti-Black racism, given its central role in influencing clinical encounters, is specially tailored for undergraduate medical students.

To address some of these curriculum challenges, the National Anti-racism in Medicine Curriculum Coalition (NAMCC) was formed in early 2020 as a multi-institutional, multispecialty collaboration of physician educators working together to develop a comprehensive anti-racism and social justice curriculum that is broadly applicable and can be disseminated across specialties to academic centers. Using a collaborative approach and tapping into the 10+ years of experience designing anti-racism curricula within the organization's leadership, NAMCC develops competency-based curricular materials that integrate case-based learning and seek to meet the needs of learners from beginner to advanced levels. A major aim of the initiative is to alleviate the resource burden placed on medical educators when they attempt to integrate anti-racism content into existing curricula. Given that the NAMCC case-based materials were in development, authors J. Corey Williams (NAMCC co-director) and Susan M. Cheng (senior associate dean of diversity, equity, inclusion & belonging) met on a biweekly basis to decide which materials would be best suited for beginner-level undergraduate medical students as well as to develop the priorities, learning objectives, and course content, utilizing intermittent consultation from external experts in the NAMCC organization.

Here, we report on the design, implementation, and evaluation of a novel undergraduate medical student course addressing manifestations of anti-Black racism for preclinical students. In partnership with Georgetown University School of Medicine faculty, members of NAMCC developed a stand-alone, 3-hour course to be delivered to a cohort of second-year medical students who were about to enter their clinical clerkships. Our team sought to design a course that would expose students to the realities of anti-Black racism in clinical practice and prepare them with practical strategies for patient advocacy. To respond to racism, our team posited that students must first learn to recognize or see it when it occurs (represented in Educational Objectives 1 and 2); therefore, we selected a case-based discussion format as the primary method of instruction to provide students with specific real-life examples and create context. Then, after the moment of recognition of a biased incident, a student should reflect on how to disrupt or redirect a situation (referred to as an upstander skills—represented in Educational Objectives 3 and 4). We also included Educational Objective 5, focusing on self-care, as we intended to raise awareness around some of the complex considerations that students undergo to be patient advocates, including taking action that might have retributive consequences, as well as strategies to obtain institutional support. Our team conceptualized the advocacy-related (or upstander) knowledge, skills, and attitudes outlined in the Educational Objectives as being core components of the competencies necessary to be an anti-racist physician.

## Methods

### Curricular Context

This course was first piloted at the Georgetown University School of Medicine for preclinical students in 2021 (*n* = 72) and delivered again in 2022 (*n* = 77). We implemented the course in the Journeys section of the curriculum, where students had the option of choosing from five different elective courses that dealt with an aspect of cultural competency in medicine. Implementing this course as an elective was necessary as space in the required curriculum was not readily available at the time. The course was situated 2 weeks before students started their clinical rotations.

Prior to the course, students had had some exposure to issues of racism in medicine across three different curricular contexts. Upon entry into medical school, this cohort of students engaged in required anti-racism summer reading and discussion groups during the first-year medical student orientation, where they read *Fatal Invention,* by Dorothy Roberts.^12^ Then, they accessed content related to anti-racism and social justice topics in an intensive first-year course entitled Patients, Populations, and Policy, which focused on the intersection of policy, social determinants of health, and the needs of marginalized populations. During this course, students viewed the documentary *13th*^13^ and reflected on their results on the Implicit Association Test,^14^ as well as engaging in a required visit to the National Museum of African American History and Culture in Washington, DC. Furthermore, first-year medical students may have been exposed to various anti-racism topics within a community-based learning course, an experiential course located at different community-based organizations.

We developed four clinical vignettes (Appendix A: Disorientating Dilemmas), based on the prior experiences of course directors and NAMCC consultants, to serve as real-life examples of how anti-Black racism could influence a clinical scenario (specific details were changed to protect identities). We designed each case to demonstrate blatant forms of racism in order to readily move beyond discussions of whether the case represented an incident of racism and towards action planning in the moment. We designed each case to emphasize a particular theme—patient care, interpersonal communication, structural and historical competency, and systems-based practice—which would eventually shape the live discussions. Then, we developed discussion questions accompanying each case, designed to encourage students to unpack how the case connected to broader histories of anti-Black racism, reflect on how the specific scenario could impact the care of the patient, and discuss potential upstander strategies.

Given the small-group discussion format, as well as the potential for strong emotional reactions to the cases, the course directors were proactive in recruiting facilitators who had both relevant content expertise and skills in facilitation. While we recruited faculty and staff facilitators with a variety of academic expertise, representing various clinical specialties, historians, and community service backgrounds, we were limited by faculty availability to participate in extensive training and preparation. Thus, we held two virtual training sessions for facilitators and recorded the training in case some facilitators were unable to attend both live sessions. During the training, facilitators reviewed their case assignments and discussed the community agreements they would use for dialogue, and we introduced them to some potential content that they could weave into the discussion. We gave them specific instructions to encourage open dialogue among the students, incorporate their own lived experiences as appropriate, and reframe misconceptions when necessary. We also gave each facilitator a facilitator guideline document (Appendix B) to review before the live session, as well as a slide deck with cases, relevant information, and discussion questions. We encouraged the facilitators to use the slides at their discretion.

### Implementation

Approximately 1 week before the live course, we emailed the students enrolled in the course the precourse materials designed to familiarize them with the core concepts (Appendix C). The precourse materials included a reflection question and an article by Bailey, Feldman, and Bassett entitled “How Structural Racism Works—Racist Policies as a Root Cause of U.S. Racial Healthy Inequalities.”^15^

Due to COVID19-related in-person restrictions, we implemented the elective course virtually in 2021 and 2022. We allotted each session of the course 3 hours on two dates (the same session with a different cohort of students on the second date). The live session opened with a whole-group slide presentation (Appendix D) that reminded students of the relevant concepts previously covered in the curriculum, established community agreements for difficult discussions, introduced new definitions, and provided the overarching purpose of the course. We explained the expectations for the format of the course and encouraged honest, courageous dialogue around the case discussions.

Subsequently, we generated virtual breakout rooms with instructions for students to join the room they had been previously assigned. Once students had joined the virtual breakout rooms (approximately eight to 10 students per room), their facilitator greeted them with brief introductions. Using the screenshare function, facilitators then showed the students the clinical vignette on a PowerPoint slide (Appendix E) and gave them time to read the case. Afterwards, the facilitators opened the discussion by asking in an open-ended manner about students’ initial thoughts and reactions, then transitioned to more specific discussion questions. After each discussion (25 minutes), students rotated through the other breakout rooms, moving in numerical order to the next case. For the closing segment, the students and facilitators joined the whole group for a debrief wherein volunteers shared key takeaways and reflections. The course directors offered some closing remarks (Appendix F) to remind students of institutional resources for their protection and support (i.e., self-care strategies) and reporting mechanisms. Finally, we gave students the last 5 minutes to complete an anonymous postcourse feedback survey (i.e., exit ticket, Appendix G).

### Evaluation Strategy

The evaluation strategy for this course included a 16-item pre- and postassessment (Appendix H; the pre and post versions were identical to allow for direct comparisons). We developed the assessment items to examine self-reported confidence level and knowledge in alignment with the course Educational Objectives. We asked students to numerically rate, using a 5-point Likert scale (1 = *strongly disagree,* 5 = *strongly agree*), how strongly they agreed with each item. Higher scores meant higher performance on the assessment. The last item asked students to identify a racial equity goal to encourage a commitment to continued engagement after the course.

We analyzed the assessment results by first calculating the mean pre- and postassessment scores for each student's response in both years (2021 and 2022). Then, given that the assessment scores were not normally distributed, we conducted the primary analysis using Wilcoxon signed rank tests, confirming the analysis with paired *t* tests. We excluded student responses that were incomplete from the analysis. In both years of the course, a small subset of students took either their pre- or postassessment during the live sessions, which created some uncertainty as to which assessment they had taken (pre vs. post). We took this into account during the data analysis by making approximate designations based on the time stamp when students began the assessment and ran the analysis with and without these responses.

The course evaluation strategy also included a postcourse anonymous feedback survey (i.e., exit ticket, Appendix G) to elicit students’ reactions, comments, and suggestions, as well as a 5-point Likert scale to numerically rate the overall course effectiveness (1 = *not effective,* 5 = *highly effective*). We designed the feedback survey questions to align with Kirkpatrick's level 1 (i.e., learner reactions) and level 2 (i.e., learning) framework for assessment^16^ and to understand how the course could be improved. Course directors worked collaboratively to read each student's comments, then grouped the comments based on a corresponding theme or element of the course. We selected the most descriptive constructive comments to illustrate the general themes that emerged and described how we planned to make changes to the course in response to each theme. This course evaluation was determined to be exempt by the Georgetown University Institutional Review Board.

## Results

**Figure 1.**
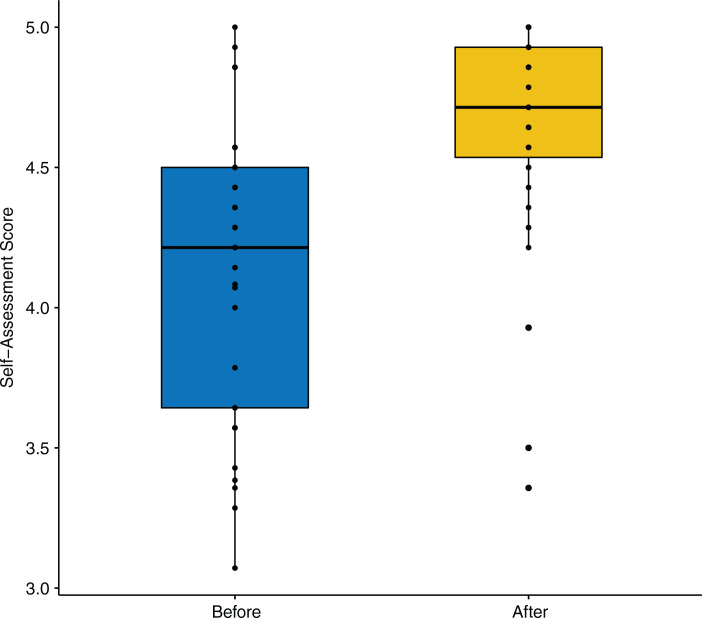
Assessment results before and after the anti-racism course in 2021 (*n* = 54), as rated on a 5-point Likert scale (1 = *strongly disagree,* 5 = *strongly agree*). The line splitting the boxes in two represents the median value. Vertical lines represent upper and lower quartiles. Tips of the vertical lines represent the extreme highs and extreme lows.

In 2021, 72 students completed the course, 54 of whom submitted the pre- and postassessments (75% response rate), including one incomplete response that was excluded from the analysis. There was a significant difference in assessment scores before and after the course for all learners combined (Wilcoxon signed rank test: median change in score = 0.54; 95% confidence interval [CI], 0.39–0.64; *V* = 40.0; *p* < .001), which is depicted in Figure 1. When we included the additional learners (*n* = 15) about whose pre versus post designation we were less confident (see Methods), the difference remained statistically significant (Wilcoxon signed rank test: median change in score = 0.43; 95% CI, 0.29–0.57; *V* = 244.5; *p* < .001), which was also robust to a paired *t* test (mean change in score = 0.53; 95% CI, 0.39–0.65; *t*(38) = 7.96; *p* < .001).

**Figure 2.**
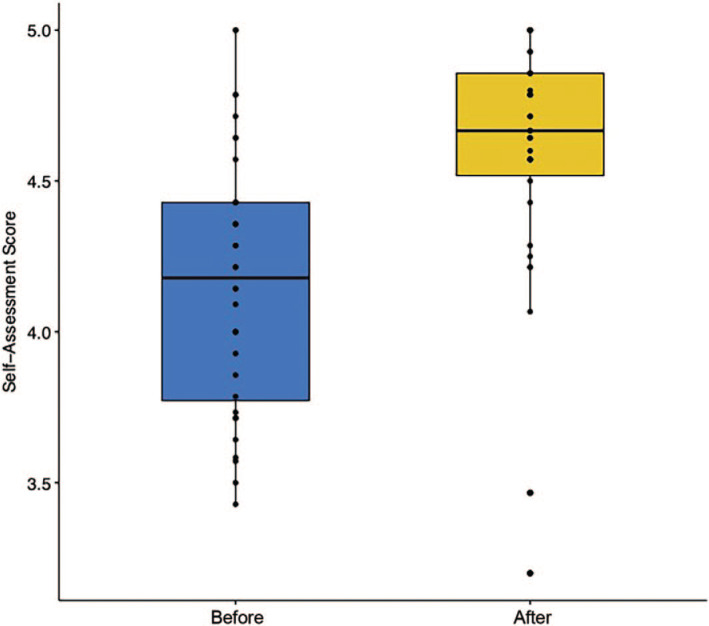
Assessment results before and after the anti-racism course in 2022 (*n* = 60), as rated on a 5-point Likert scale (1 = *strongly disagree,* 5 = *strongly agree*). The line splitting the boxes in two represents the median value. Vertical lines represent upper and lower quartiles. Tips of the vertical lines represent the extreme highs and extreme lows.

In 2022, 77 students completed the course, 60 of whom submitted the pre- and postassessments (78% response rate), including five incomplete responses that were excluded from the analysis. There was a significant difference in assessment scores before and after the course for all learners combined (Wilcoxon signed rank test: median change in score = 0.46; 95% CI, 0.32–0.59; *V* = 40.0; *p* < .001), which is depicted in Figure 2. When we included the additional learners (*n* = 22) about whose pre versus post designation we were less confident (see Methods), the difference remained statistically significant (Wilcoxon signed rank test: median change in score = 0.25; 95% CI, 0.08–0.42; *V* = 376.0; *p* < .001), which was also robust to a paired *t* test (mean change in score = 0.42; 95% CI, 0.29–0.54; *t*(32) = −6.67; *p* < .001). While items in the assessment appeared to capture different domains of learning (e.g., basic knowledge, self-confidence, etc.), our team conceptualized the assessment as likely capturing a singular anti-racism-related construct, which was supported by high correlations between the items—assessed post hoc via Cronbach's alpha using the pretest from all learners who answered all items in either year (α = .88; bootstrap 95% CI, 0.84–0.91).

**Figure 3.**
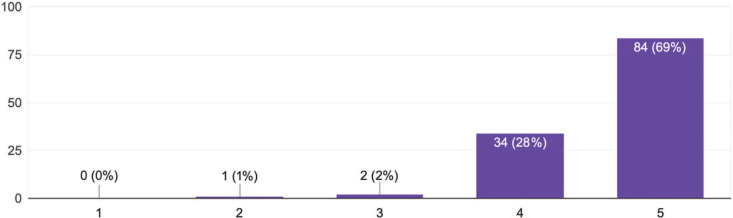
Results of an anonymous feedback survey from medical students (*n* = 121) rating overall course effectiveness on a 5-point Likert scale (1 = *not effective,* 5 = *highly effective*).

The postcourse anonymous feedback survey (Appendix G) had a response rate of 88% and 75% in 2021 and 2022, respectively. The Likert item assessing overall course effectiveness (1 = *not effective,* 5 = *highly effective*) showed that 69% of students rated the course as highly effective and 28% rated the course as somewhat effective; combined, 97% rated the course as at least somewhat effective (Figure 3). Our team iteratively reviewed the positive comments within the constructed responses from the exit ticket, and two broad themes emerged. We selected certain student quotations descriptive of the corresponding theme:

1. Theme: The course helped students understand and recognize different examples of racism.
  • “The way that myths about race and racial prejudices have been passed down through history and continue to impact the way that minorities, especially Blacks, are mistreated in our healthcare system. I understand better how systemic racism manifests itself within a clinical setting and how Black patients and providers are affected. I better understand how I can intervene and promote anti-racist thought and behavior.”
  • “It really taught me to question a lot of the race-based practices that we have learned in our education and wonder what other myths are out there that we naively accept as truths.”
2. Theme: The course taught students actionable strategies for confronting racism.
  • “How to combat racism, how to deal with anti-Blackness, what IS anti-Blackness. I also learned about the certain ‘racial correction factors’ (actually had never heard of these) and how to be an ally to Black people as well as people who are subject to any sort of racism/stereotyping in the medical setting.”
  • “This session helped me contextualize the forms that anti-Black racism and bias could take in the healthcare setting, and I appreciated the exposure to the different ways of response. Although I'm by no means well-versed on the topic, I feel like I would be less of a deer in headlights if I were to encounter similar situations during clerkships.”

In regard to the constructive feedback (i.e., in response to “How could this course be improved?”), five primary themes emerged: course timing, case complexity, learner differentiation, direct instruction, and access to resources. The Table shows the most descriptive comments that align with each theme and how we plan to change the course in response to these comments.

**Table.**
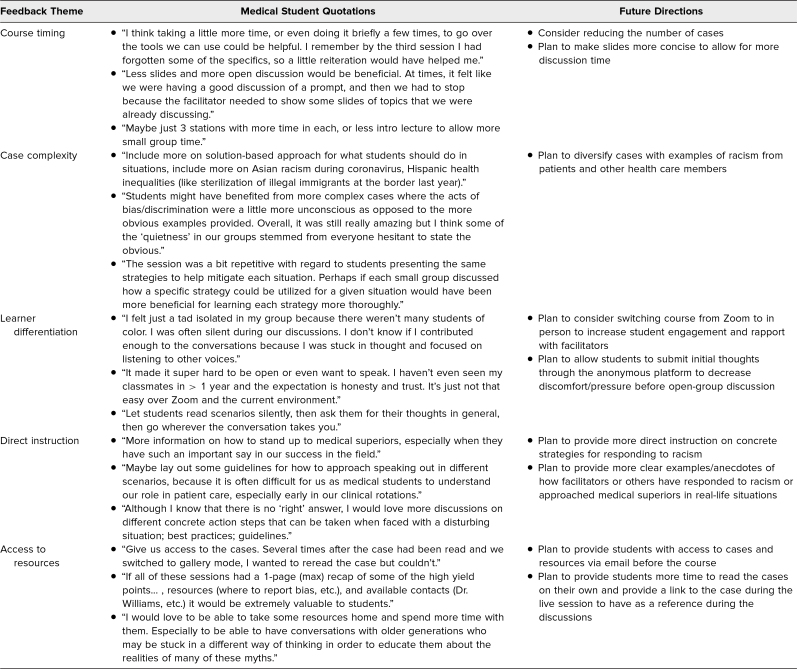
Qualitative Constructive Feedback in Response to the Prompt “How Could the Session Be Improved?”

## Discussion

This course sought to build upon the existing curricular resources that utilize case-based vignettes to facilitate deep reflection and dialogue among medical students, in order to advance the anti-racist competencies of undergraduate medical students. Specifically, the course sought to advance students’ skills in recognizing how anti-Blackness may influence a clinical encounter and being able to explicitly name the entrenched nature of anti-Blackness in the culture and history of medicine writ large. With these learning goals articulated and reinforced throughout the course, we hoped that learners would be more prepared to redirect, reframe, and interrupt processes influenced by racism as incidents arose in the clinical context. We implemented the course with second-year preclinical students who were soon to transition into their clinical rotations, which presented a valuable opportunity to prepare them to engage in advocacy on behalf of Black-identifying patients and colleagues.

Overall, students received the course positively, with 97% rating it as effective (sum of somewhat effective and highly effective responses), as measured by the anonymous postcourse survey or exit ticket. With regard to self-assessed learning, two core themes repeatedly emerged from the exit ticket responses: (1) The course helped students understand and recognize different examples of racism, and (2) the course taught students actionable strategies for confronting racism. A review of the constructive student comments resulted in five core themes: course timing, case complexity, learner differentiation, direct instruction, and access to resources. Of note, we received mixed comments about the course timing (some wanted more time, others advocated for less). While we intentionally designed the cases to represent blatant forms of racism, several students suggested varying their complexity to include more subtle or nuanced forms of bias.

Our team observed that the case discussion-based format proved a significant challenge for student engagement, particularly at the start of the live discussions. The facilitators noticed that several students in the groups were reluctant to share, which was consistent with some student comments in the postcourse survey where students cited discomfort with the format. Given the sensitivity of the content, this course requires skilled facilitators trained in anti-oppressive facilitation techniques—such skills likely go beyond what can be covered in the 2 hours of training we offered. We attempted to overcome this limitation by recruiting faculty who we believed already had a sufficient threshold of content expertise and facilitation experience, which significantly shrunk the pool of potential faculty instructors. The potential for other institutions, especially for small ones (who may have smaller pools of faculty expertise), to reproduce this course may be limited by the need for expert facilitation given the sensitivity of the topics.

The elective nature of the course represents one of the major limitations; meaning that students who participated likely self-selected into the course based on their preexisting interests, which may have resulted in a particular cohort already engaged in anti-racism learning. Another key limitation extended from the evaluation strategy, which relied heavily upon Kirkpatrick's level 1 framework, where students self-reported acquisition of knowledge and confidence (as opposed to direct or observational assessments).

Given the observed student discomfort with the discussion-based format, we employed several techniques to encourage engagement (with mixed results), such as beginning with community agreements, providing skilled facilitation, and offering reassurance to students that the discussions were nonevaluative and nonjudgmental. We also encouraged students to turn off Zoom cameras if they felt overwhelmed by the material in order to prioritize wellness during the sessions. Overall, we noticed that as each group rotated through the progression of the four cases, dialogues seemed to flow more naturally with richer student engagement.

Some key lessons learned from the student feedback included that students appeared to want access to course resources (cases, slides, references) during and after the course, as well as more concrete guidelines for how to approach speaking out in different scenarios, and that some students expressed discomfort with speaking in an unfamiliar group.

A core challenge of this course was striking a balance between students’ need to learn from real cases involving racism while not inducing undue stress on BIPOC (Black, Indigenous, and people of color) students who may have personal histories of race-based stress and/or trauma. On the other hand, BIPOC students may find these explicit discussions about the realities of racism in medicine to be validating and empowering, so student reactions and receptivity are unpredictable. Based on some postcourse survey comments, we suspect there was a differential impact of the case content and discussion on BPIOC students, as some found the case content activating and expressed discomfort with talking about the cases or their own lived experience of racism (although we did not formally collect racial demographic information). We recommend that, beginning at the early stages of student enrollment, future course directors be clear and explicit that course material directly deals with incidents of racism, which can be distressing to discuss. With this clear messaging before the course, as well as the course's nature as an elective, students can opt out if they are uncertain about their ability to tolerate the material. Of note, future course directors should favor an in-person format (pending institutional in-person restrictions), which may facilitate deeper engagement.

Also, course directors should consider learner-centered differentiation strategies, whereby learners self-report lived experience (affinity group membership or racial demographic data); then, students can be grouped based on their responses, and course material can be adjusted based on the groupings. Furthermore, the sensitive nature of the content increases the need for expert anti-oppressive facilitation skills. Faculty facilitators should be trained and prepared to redirect or challenge oppressive perspectives and find ways to express solidarity with minoritized students, for example. At the course's conclusion, we recommend that facilitators remind students where they can access mental health resources at their local institutions.

Future curriculum reform efforts should expand beyond electives to embed the content in the fabric of the curriculum. Similar to other basic competencies in medicine (e.g., physical exam, clinical interviewing, etc.), students must have multiple longitudinal opportunities to practice anti-racism knowledge and skills. Future efforts should expand on the evaluation to incorporate clinical assessments to ascertain whether students use the upstander skills or have increased awareness of potential biases in real-life situations. Furthermore, future courses should consider incorporating student demographic information into the analysis to assess if the course is more effective for specific groups. Similarly, faculty must also have parallel longitudinal opportunities for ongoing professional development in anti-racism and anti-oppression if they are to lead curricular discussions and scholarly research in this arena. We hope that a course like this one can serve as a building block and help catalyze a wider movement of curriculum reform toward anti-racist perspectives. Ultimately, these reforms represent a broad shift in medical education, practice, and research that will ultimately necessitate an institution-wide effort and firm commitments from academic leadership that also inherently involve faculty development and accountability.

## Appendices


Disorienting Dilemmas.docxFacilitator Guidelines.docxPrework Module.docxOpening Slides.pptxFacilitator Slides.pptxClosing Remarks Slides.pptxExit Ticket.docxPre- and Postassessment.docx
*All appendices are peer reviewed as integral parts of the Original Publication.*
