# Correction: Core Competencies of an Anti-racist Physician: Elective Course for Undergraduate Medical Students

**DOI:** 10.15766/mep_2374-8265.11486

**Published:** 2024-11-06

## Correction

In the publication “Core Competencies of an Anti-racist Physician: Elective Course for Undergraduate Medical Students,” Aaron Alexander-Bloch's surname was misspelled Alexandar-Bloch. The original publication has been corrected.
