# Supplementary material for: Core Competencies of an Anti-racist Physician: Elective Course for Undergraduate Medical Students
Source: MedEdPORTAL. 2024 May 14;20:11395. doi: 10.15766/mep_2374-8265.11395 (PMC11219086; doi:10.15766/mep_2374-8265.11395)
Supplement: Supplementary file 1 — Disorienting Dilemmas.docxFacilitator Guidelines.docxPrework Module.docxOpening Slides.pptxFacilitator Slides.pptxClosing Remarks Slides.pptxExit Ticket.docxPre- and Postassessment.docx [file mep_2374-8265.11395-s001.zip › A. Disorienting Dilemmas.docx]

**Appendix A**

**Case 1: Patient Care**

You are a medical student rotating on a psychiatry consult-liaison service. You are called to see a 16-year-old African American female who presented to the ED accompanied by her biological parents after a suicide attempt via overdose. After you obtain a history, you leave the room to go present it to the attending, who is an older white male. You and the attending decide that the patient is in need of acute psychiatric hospitalization.

You go back to the patient’s room along with the attending to speak with the patient and her parents. You notice that as the attending began the conversation, he was verbally dominant and gave no opportunity for the parents to express their concerns or to ask questions. You notice that he was speaking in a decisive and assertive tone describing that the patient “will need to go to an inpatient facility” and “here’s what’s going to happen.” He went on to explain the process of being admitted to an inpatient psychiatric facility. It is your impression that the attending was being condescending to the parents and explaining things in an overly simplistic manner as if he were expecting them to not understand. He asserts that the parents need to explain this decision to their daughter, so she will sign for a voluntary inpatient admission. The family appears speechless. After he finishes speaking, the attending suddenly stands up and walks out of the room. You follow him out of the room.

Several hours later, you return to the patient’s room to check in with the family. The father, appearing upset, signals to you that he would like to speak outside the room. He states, “We do not want to deal with that attending ever again. We will only deal with you from now on. There is a certain way that you speak to people. I mean, I understand that we are new to this process, but there is a certain way that you talk to people. If we had been a white family, I doubt he would have talked to us like that. Do you know what I mean?”

**Case 2: Interpersonal Communication**

You and another medical student are rotating on a Gastroenterology unit together. You and your medical student colleague (who identifies as African American) are rounding with your attending physician. The attending is concerned that one of the patients had an episode of bloody stool overnight. You, your colleague, and the attending are at the bedside, and the attending is trying to get the patient to clarify the color of the stools. The patient is struggling to remember the color of the stool. The physician grabs the right forearm of your colleague and pulls it towards the patient. The physician asks the patient, “Does it [the patient’s diarrhea color] look like her arm or is it lighter?”…The physician and patient laugh. The patient states, “a little lighter than that.” The attending says to your colleague, “See, your skin is the perfect color for this job”.

**Case 3: Structural and Historical Competency**

*Part 1:*

You are a medical student rotating on a labor and delivery floor during your OB clerkship. You have just admitted an African American-identifying mother for a planned C-section. As a routine part of the pre-operative assessment, the team (you, a resident, and a OB nurse) is speaking to the mother about her potential options for birth control or contraception after the procedure. The attending asks the patient if she would like to have her Fallopian tubes tied after the C-section is completed. The patient responds that she would like to have more children in the future, so she is not interested in preventing future pregnancies. The team then leaves the room and starts to walk toward the next patient’s room.

The resident and the nurse are chatting on the way to the next room. You overhear the resident say, “I figured she would refuse. A lot of these types of patients are just having babies out of control and not being able to afford them. What happens is, we end up delivering their babies and costing the system a ton of money.”

*Part 2:*

While admitted, the fetus has an episode of bradycardia, and the planned C-section switches to an emergency C-section. You and the resident rush the mother to the OR. After a successful procedure, you scrub out and begin working on your note when you notice the OB nurse approaching you.

The OB nurse taps you on the shoulder and says, “Let me give you some advice since you are so early in your training. I used to be a nurse in the NICU for many years, and I noticed differences in how White and Black babies typically respond to their illnesses. I noticed that the Black girls were particularly resilient; they would fight through whatever was going on with them and pull through. And, I noticed the White baby boys were especially sensitive and fragile; they would always succumb to whatever the sickness was. The Black babies overall were much more resilient than the White babies; therefore, the White babies need to be cared for and watched much more closely, and the Black babies do not need to be treated as urgently.”

**Case 4: Systems-based Practice**

*Part 1:*

You are on a surgery rotation and are doing an overnight trauma call shift. You are called down to the critical care unit to assist the resident on a level one trauma case. Lying in room 4 is a 26-year-old African-American man with a bone fracture to the left humerus that resulted from a physical altercation in the community. His vitals are stable, but he is crying out in pain. You examine the patient with the resident physician, and you are taking notes as the resident voices the results of the primary and secondary trauma assessment. Upon completion of the exam, the resident determines that the patient does not need surgical intervention. When you ask what the patient will receive for pain control, the resident responds, “He is probably exaggerating. A lot of times these patients are trying to get opiates while they are in the hospital.” You feel confused at that moment. The previous patient you saw with this resident was a 56-year-old woman with acute abdominal pain, and the resident wanted to give hydromorphone (an opiate) for the pain she was experiencing.

*Part 2:*

After 24 hours of observation, the patient’s blood pressure increases, and his kidney function begins to decline (i.e. increase in creatinine levels). You are working with the resident before morning rounds to come up with a plan. You watch the resident calculate the current eGFR using a racial correction factor. The resident reports the eGFR during rounds and suggests Lisinopril because “the literature” suggests that Black patients respond better to ACE inhibitors.
