# Supplementary material for: Core Competencies of an Anti-racist Physician: Elective Course for Undergraduate Medical Students
Source: MedEdPORTAL. 2024 May 14;20:11395. doi: 10.15766/mep_2374-8265.11395 (PMC11219086; doi:10.15766/mep_2374-8265.11395)
Supplement: Supplementary file 1 — Disorienting Dilemmas.docxFacilitator Guidelines.docxPrework Module.docxOpening Slides.pptxFacilitator Slides.pptxClosing Remarks Slides.pptxExit Ticket.docxPre- and Postassessment.docx [file mep_2374-8265.11395-s001.zip › B. Facilitator Guidelines.docx]

# Case 1 Facilitator Guide: Patient Care Theme

Instructions:

Each break-out room (25 minutes each) is broken into two parts: case discussion and clinical advocacy discussion. About 15 minutes into the case discussion, facilitators should transition to the clinical advocacy discussion focused on responding to the incident. Break-out rooms should largely be student-centered small group dialogues. Encourage students to respond to one another. Please use the discussion questions to guide the dialogue but feel free to leave space for spontaneous discussion based on important issues that may emerge. The slides are available to use at your discretion to help you solidify and drive home key points of the case. Please avoid lecturing for extended periods of time.

Prep work:

1. Review the slides, key points, and discussion questions before live-session
2. Feel free to adapt the slides or add slides of your own

Course agenda for the live session (150 minutes total)

- - Opening & Introduction – 15 minutes (Appendix D)
  - Small group case-based discussions – 100 minutes total (25 minutes for each case discussion including a 10-minute break halfway through; Appendix E)
    - Case #1: Patient care
    - Case #2: Interpersonal Communication
    - Case #3: Structural & Historical Competency
    - Case #4: Systems-based Practice
  - Whole group debrief & reflection – 10 min
  - Closing remarks – 5 min (Appendix F)
  - Post-course feedback survey (Appendix G)

Live session:

1. Introduce yourself
2. Give the students time to read the case
3. For part 1 of the discussion, guide students through a small group dialogue using the following questions:
   1. How do implicit and explicit bias influence clinical communication (including verbal and non-verbal communication)?
   2. How could this physician's communication (and behavior) be an example of anti-B lack racism?
   3. How might these comments (and behavior) operate to impact the care of the patient?
4. For part 2 of the discussion, guide the students through clinical advocacy questions:
   1. How could you navigate the power dynamics in this situation to advocate for the patient?
   2. What strategies could be used to give feedback to the attending?
   3. How could this situation be used to foster systematic change?
   4. What are the potential barriers to addressing this situation?

**Key points to emphasize during the discussion:**

- Racism and other forms of bias can be enacted through poor communication with patients
- Physicians are more verbally dominant and engaged in less patient-centered communication with African American patients than with White patients. Physicians exhibit less positive affect during clinical encounters with African American patients when compared to White patients^1^.
- There is also evidence that physicians deliver less information to, spend less time and engage in less supportive talk with minoritized racial/ethnic groups as compared to non-minoritized patients^2-3^ and display differences in nonverbal communications^4-5^.
- **“Take Action” Framework**^6^ can be used to identify advocacy strategies:
  - **A**sk clarifying questions to assist with understanding intentions.
  - **C**ome from curiosity not judgment.
  - **T**ell what you observed as problematic in a factual manner.
  - **I**mpact exploration: ask for, and/or state, the potential impact of such a statement or action on others.
  - **O**wn your own thoughts and feelings around the impact.
  - **N**ext steps: Request appropriate action be taken.

**References**

1. Johnson RL, Roter D, Powe NR, Cooper LA. Patient race/ethnicity and quality of patient–physician communication during medical visits. American journal of public health. 2004 Dec;94(12):2084-90.
2. Smedley, B.D., Stith, A.Y. and Nelson, A.R., 2003. Patient-provider communication: the effect of race and ethnicity on process and outcomes of healthcare. In *Unequal Treatment: Confronting Racial and Ethnic Disparities in Health Care*. National Academies Press (US).
3. Blendon RJ, Buhr T, Cassidy EF, Pérez DJ, Sussman T, Benson JM, Herrmann MJ. Disparities in physician care: experiences and perceptions of a multi-ethnic America. Health Affairs. 2008 Mar;27(2):507-17.
4. Elliott AM, Alexander SC, Mescher CA, Mohan D, Barnato AE. Differences in physicians' verbal and nonverbal communication with B lack and white patients at the end of life. Journal of pain and symptom management. 2016 Jan 1;51(1):1-8.
5. Robinson JD, Jagsi R. Physician-patient communication—an actionable target for reducing overly aggressive care near the end of life. JAMA oncology. 2016 Nov 1;2(11):1407-8.
6. Cheung FL, Ganote C, Souza T. Microaggressions and microresistance: supporting and empowering students. Diversity and inclusion in the college classroom. 2016;15.

# Case 2 Facilitator Guide: Interpersonal Communication Theme

Instructions: Each break-out room (25 minutes each) is broken into two parts: case discussion and clinical advocacy discussion. About 15 minutes into the case discussion, facilitators should transition to the clinical advocacy discussion focused on responding to the incident. Break-out rooms should largely be student-centered small group dialogues. Encourage students to respond to one another. Please use the discussion questions to guide the dialogue but feel free to leave space for spontaneous discussion based on important issues that may emerge. The slides are available to use at your discretion to help you solidify and drive home key points of the case. Please avoid lecturing for extended periods of time.

Prep work:

1. Review the slides, key points, and discussion questions before live-session
2. Feel free to adapt the slides or add slides of your own

Course agenda for the live session (150 minutes total)

- - Opening & Introduction – 15 minutes (Appendix D)
  - Small group case-based discussions – 100 minutes total (25 minutes for each case discussion including a 10-minute break halfway through; Appendix E)
    - Case #1: Patient care
    - Case #2: Interpersonal Communication
    - Case #3: Structural & Historical Competency
    - Case #4: Systems-based Practice
  - Whole group debrief & reflection – 10 min
  - Closing remarks – 5 min (Appendix F)
  - Post-course feedback survey (Appendix G)

Live session:

1. Introduce yourself
2. Give the students time to read the case
3. For part 1 of the discussion, guide students through a small group dialogue using the following questions:

- How is this a manifestation of anti-B lack racism?
- What impact do you think this will have on your student colleague?
- What does it mean to be complicit? Who was complicit in this encounter?

1. For part 2 of the discussion, guide the students through clinical advocacy questions:

- How would you address what happened with your colleague?
- How could you navigate this situation to be anti-racist and advocate for your colleague? How do you address the power dynamics in this interaction?
- What are the barriers to addressing mistreatment and harassment by the clinical team?
- How do addressing mistreatment and harassment differ as experienced by the patient vs. when the target is a colleague?

**Key points to emphasize during the discussion:**

- In a JAMA study, researchers analyzed 27,500 graduation questionnaires, which represent 72.1% of medical school graduates in 2016 and 2017. More than one-third of survey respondents reported experiencing at least one type of mistreatment. The most common form of mistreatment was public humiliation, which was reported by 21.1% of survey respondents^1^.
- Among White students, 24.0% reported experiencing mistreatment. Reported rates of mistreatment were higher for Asian (31.9%), underrepresented (38.0%), and multiracial (32.9%) students. Women, minoritized racial and ethnic groups, and minoritized sexual identities bear a disproportionate share of medical student mistreatment^1^.
- Mistreatment of students includes a range of actions such as assault, discrimination, sexual harassment, and verbal abuse. Mistreatment has been linked to several negative consequences, including alcohol abuse, burnout, depression, and medical student attrition^2^.
- **“Take Action” Framework**^3^ can be used to identify advocacy strategies:
  - **A**sk clarifying questions to assist with understanding intentions.
  - **C**ome from curiosity not judgment.
  - **T**ell what you observed as problematic in a factual manner.
  - **I**mpact exploration: ask for, and/or state, the potential impact of such a statement or action on others
  - **O**wn your own thoughts and feelings around the impact.
  - **N**ext steps: Request appropriate action be taken.

**References:**

1. Hill KA, Samuels EA, Gross CP, Desai MM, Zelin NS, Latimore D, Huot SJ, Cramer LD, Wong AH, Boatright D. Assessment of the prevalence of medical student mistreatment by sex, race/ethnicity, and sexual orientation. JAMA internal medicine. 2020 May 1;180(5):653-65.
2. Anderson N, Lett E, Asabor EN, Hernandez AL, Nguemeni Tiako MJ, Johnson C, Montenegro RE, Rizzo TM, Latimore D, Nunez-Smith M, Boatright D. The association of microaggressions with depressive symptoms and institutional satisfaction among a national cohort of medical students. Journal of General Internal Medicine. 2022 Feb;37(2):298-307.
3. Cheung FL, Ganote C, Souza T. Microaggressions and microresistance: supporting and empowering students. Diversity and inclusion in the college classroom. 2016;15.

# Case 3 Facilitator Guide: Structural and Historical Competency Theme

Instructions: Each break-out room (25 minutes each) is broken into two parts: case discussion and clinical advocacy discussion. About 15 minutes into the case discussion, facilitators should transition to the clinical advocacy discussion focused on responding to the incident. Break-out rooms should largely be student-centered small group dialogues. Encourage students to respond to one another. Please use the discussion questions to guide the dialogue but feel free to leave space for spontaneous discussion based on important issues that may emerge. The slides are available to use at your discretion to help you solidify and drive home key points of the case. Please avoid lecturing for extended periods of time.

Prep work:

1. Review the slides, key points, and discussion questions before live-session
2. Feel free to adapt the slides or add slides of your own

Course agenda for the live session (150 minutes total)

- - Opening & Introduction – 15 minutes (Appendix D)
  - Small group case-based discussions – 100 minutes total (25 minutes for each case discussion including a 10-minute break halfway through; Appendix E)
    - Case #1: Patient care
    - Case #2: Interpersonal Communication
    - Case #3: Structural & Historical Competency
    - Case #4: Systems-based Practice
  - Whole group debrief & reflection – 10 min
  - Closing remarks – 5 min (Appendix F)
  - Post-course feedback survey (Appendix G)

Live session:

1. Introduce yourself
2. Give the students time to read the case
3. For part 1 of the discussion, guide students through a small group dialogue using the following questions:

Case section 1.

- What is the problem with these comments?
- How might these comments operate to impact the care of the patient?
- What anti-B lack history do these comments connect to?

Case section 2.

- Are these remarks true? Why or why not?
- How might she have come to this conclusion?
- How is this seemingly positive remark (i.e. resilient Black babies) a manifestation of anti-Blackness?
- How might these comments ultimately impact the care of the patient or the infant?

1. For part 2 of the discussion, guide the students through clinical advocacy questions:

- What strategies could you use to redirect and educate this individual?
- How could you navigate this situation to be an antiracist and advocate for the patient?

**Key points to emphasize in the discussion:**

- The stereotype of the so-called 'welfare queen' has been used to demonize those on public assistance for decades. It's historically been a politically potent image, depicting an undeserving aid recipient getting rich on the backs of taxpayers. The welfare queen stood in for the idea that Black women were too lazy to work, instead relying on public benefits to get by, paid for by the rest of us upstanding citizens^1^.
- The welfare queen stereotype depicted Black women as promiscuous, having as many children as possible in order to increase her benefit. It was always a myth—White people have always made up the majority of those receiving government assistance, and if anything, benefits are too miserly, not too lavish. Nevertheless, it was a potent stereotype, which helped fuel the political will to dismantle social safety nets^1^.
- This stereotype continues to operate in healthcare settings as certain healthcare workers may express explicit or implicit resentment towards patients they deem as undeserving of services.
- James Marion Sims — “father of modern gynecology” – from 1845 to 1849, conducted experimental vesicovaginal fistula surgery on 11 enslaved people several times and one woman (Anarcha) 30 times; n o anesthesia was used despite it being available.
- Enslaved Black women were forced to hold each other down. Dr. Sims k nowingly violated AMA Code of Ethics rules for multiple surgeries on the same patients^2^.
- Sims also experimented on enslaved infants, according to the medical journal Social History of Medicine. He tested surgical treatments on enslaved Black children in an effort to treat “trismus nascentium” (neonatal tetanus)—with little to no success. Sims also believed that African Americans were less intelligent than White people, and thought it was because their skulls grew too quickly around their brains. He would operate on African American children using a shoemaker’s tool to pry their bones apart and loosen their skulls. The procedure was fatal^2^.
- U.S. physicians have a long history of delegitimizing Black women’s claims to motherhood typified by the history of forced sterilizations^4^. Further, the health system has an entrenched history of objectifying Black women and disregarding their pain.
- Anti-B lack racism is built into the treatment of Black women and infants. This is exemplified by continued disparities in infant and maternal mortalities^3^.
- **“Take Action” Framework**^5^ can be used to identify advocacy strategies:
  - **A**sk clarifying questions to assist with understanding intentions.
  - **C**ome from curiosity not judgment.
  - **T**ell what you observed as problematic in a factual manner.
  - **I**mpact exploration: ask for, and/or state, the potential impact of such a statement or action on others
  - **O**wn your own thoughts and feelings around the impact.
  - **N**ext steps: Request appropriate action be taken.

**References:**

1. Gilman ME. The return of the welfare queen. Am. UJ Gender Soc. Pol'y & L. 2013;22:247.
2. Washington HA. Medical apartheid: The dark history of medical experimentation on Black Americans from colonial times to the present. Doubleday Books; 2006.
3. Bridges KM. Racial disparities in maternal mortality. NYUL Rev. 2020;95:1229.
4. Stern A. Forced sterilization policies in the US targeted minorities and those with disabilities-and lasted into the 21st century. The Conversation. https://theconversation. com/forced-sterilization-policies-in-the-us-targeted-minorities-and-those-with-disabilities-and-lasted-into-the-21st-century-143144. 2021.
5. Cheung FL, Ganote C, Souza T. Microaggressions and microresistance: supporting and empowering students. Diversity and inclusion in the college classroom. 2016;15.

# Case 4 Facilitator Guide: Systems-Based Practice Theme

Instructions: Each break-out room (25 minutes each) is broken into two parts: case discussion and clinical advocacy discussion. About 15 minutes into the case discussion, facilitators should transition to the clinical advocacy discussion focused on responding to the incident. Break-out rooms should largely be student-centered small group dialogues. Encourage students to respond to one another. Please use the discussion questions to guide the dialogue but feel free to leave space for spontaneous discussion based on important issues that may emerge. The slides are available to use at your discretion to help you solidify and drive home key points of the case. Please avoid lecturing for extended periods of time.

Prep work:

1. Review the slides, key points, and discussion questions before live-session
2. Feel free to adapt the slides or add slides of your own

Course agenda for the live session (150 minutes total)

- - Opening & Introduction – 15 minutes (Appendix D)
  - Small group case-based discussions – 100 minutes total (25 minutes for each case discussion including a 10-minute break halfway through; Appendix E)
    - Case #1: Patient care
    - Case #2: Interpersonal Communication
    - Case #3: Structural & Historical Competency
    - Case #4: Systems-based Practice
  - Whole group debrief & reflection – 10 min
  - Closing remarks – 5 min (Appendix F)
  - Post-course feedback survey (Appendix G)

Live session:

1. Introduce yourself
2. Feel free to adapt the slides or add slides of your own
3. For part 1 of the discussion, guide students through a small group dialogue using the following questions:

Case section 1.

- What role might stereotyping or bias be playing here?
- What history does this racist thinking connect to?

Case section 2.

- What is a racial correction factor? What are some examples of racial correction factors?
- What is wrong with racial correction factors? How are systematized racial corrections problematic and harmful?

1. For part 2 of the discussion, guide the students through clinical advocacy questions:

- What strategies could you use to redirect and educate this individual?
- How could you navigate this situation to be antiracist and advocate for the patient?

**Key points to emphasize in the discussion:**

- There is emerging literature documenting disparities in pain care among minoritized racial and ethnic groups across a wide variety of pain conditions and treatment settings. African Americans and Hispanics are more likely to be undertreated for pain than White patients across settings, types of pain (e.g., acute vs chronic), and populations (e.g., adults vs children)^1.^
- In one analysis of pediatric emergency room visits, minoritized children, when compared to non-Hispanic (NH) White children, were more likely to receive any analgesics, and achieve ≥2-point reduction in pain, but were less likely to receive opioids or achieve optimal pain reduction^2^.
- Longitudinal, national data on 156,729 pain-related emergency department visits found that even among patients presenting with the same condition, non-Hispanic White patients were significantly more likely to receive an opioid than all other minoritized ethnic groups examined^3^.
- Even subtle insertion of patient racial identity into a clinical vignette activates diagnostic algorithms and practice guidelines that adjust or “correct” their outputs on the basis of a patient’s race. Physicians use these algorithms to individualize risk assessment and guide clinical decisions. By embedding race into the basic data and decisions of health care, these algorithms propagate race-based medicine. Many of these race-adjusted algorithms guide decisions in ways that may direct more attention or resources to White patients than to members of minoritized racial and ethnic groups ^4^.
- **“Take Action” Framework**^5^ can be used to identify advocacy strategies:
  - **A**sk clarifying questions to assist with understanding intentions.
  - **C**ome from curiosity not judgment.
  - **T**ell what you observed as problematic in a factual manner.
  - **I**mpact exploration: ask for, and/or state, the potential impact of such a statement or action on others
  - **O**wn your own thoughts and feelings around the impact.
  - **N**ext steps: Request appropriate action be taken.

**References:**

1. Bonham VL. Race, ethnicity, and pain treatment: Striving to understand the causes and solutions to the disparities in pain treatment. Journal of Law, Medicine & Ethics. 2001;29(1):52-68.
2. Mills AM, Shofer FS, Boulis AK, Holena DN, Abbuhl SB. Racial disparity in analgesic treatment for ED patients with abdominal or back pain. The American journal of emergency medicine. 2011 Sep 1;29(7):752-6.
3. Pletcher MJ, Kertesz SG, Kohn MA, Gonzales R. Trends in opioid prescribing by race/ethnicity for patients seeking care in US emergency departments. Jama. 2008 Jan 2;299(1):70-8.
4. Roberts D. Fatal invention: How science, politics, and big business re-create race in the twenty-first century. New Press/ORIM; 2011 Jun 14.
5. Cheung FL, Ganote C, Souza T. Microaggressions and microresistance: supporting and empowering students. Diversity and inclusion in the college classroom. 2016;15.
