# Supplementary material for: Core Competencies of an Anti-racist Physician: Elective Course for Undergraduate Medical Students
Source: MedEdPORTAL. 2024 May 14;20:11395. doi: 10.15766/mep_2374-8265.11395 (PMC11219086; doi:10.15766/mep_2374-8265.11395)
Supplement: Supplementary file 1 — Disorienting Dilemmas.docxFacilitator Guidelines.docxPrework Module.docxOpening Slides.pptxFacilitator Slides.pptxClosing Remarks Slides.pptxExit Ticket.docxPre- and Postassessment.docx [file mep_2374-8265.11395-s001.zip › C. Prework Module.docx]

**Pre-course material:** Core Competencies of the Anti-Racist Physician

**Instructions:** Participation in this pre-course material will take approximately 20 to 30 minutes. Participation is completely voluntary; responses are kept strictly confidential.

**Reflection question:** Briefly in 3 to 5 sentences, please answer the following reflection question: Doyou believe that racism, specifically anti-Black racism, influences the clinical practice of medicine? Why or why not? Please provide specific examples.

**Self-assessment:** On a scale of 1-5, how easy or difficult was it to respond to the reflection question?

1. Very easy
2. Somewhat easy
3. Neutral
4. Somewhat difficult
5. Very difficult

**Learning resource:** Please read the following article – Bailey ZD, Feldman JM, Bassett MT. How Structural Racism Works — Racist Policies as a Root Cause of U.S. Racial Health Inequities. New England Journal of Medicine [Internet]. 2021 Feb 25 [cited 2022 Jul 6];384(8):768–73. Available from: https://doi.org/10.1056/NEJMms2025396
