# Supplementary material for: Core Competencies of an Anti-racist Physician: Elective Course for Undergraduate Medical Students
Source: MedEdPORTAL. 2024 May 14;20:11395. doi: 10.15766/mep_2374-8265.11395 (PMC11219086; doi:10.15766/mep_2374-8265.11395)
Supplement: Supplementary file 1 — Disorienting Dilemmas.docxFacilitator Guidelines.docxPrework Module.docxOpening Slides.pptxFacilitator Slides.pptxClosing Remarks Slides.pptxExit Ticket.docxPre- and Postassessment.docx [file mep_2374-8265.11395-s001.zip › D. Opening Slides.pptx]

## Slide 1
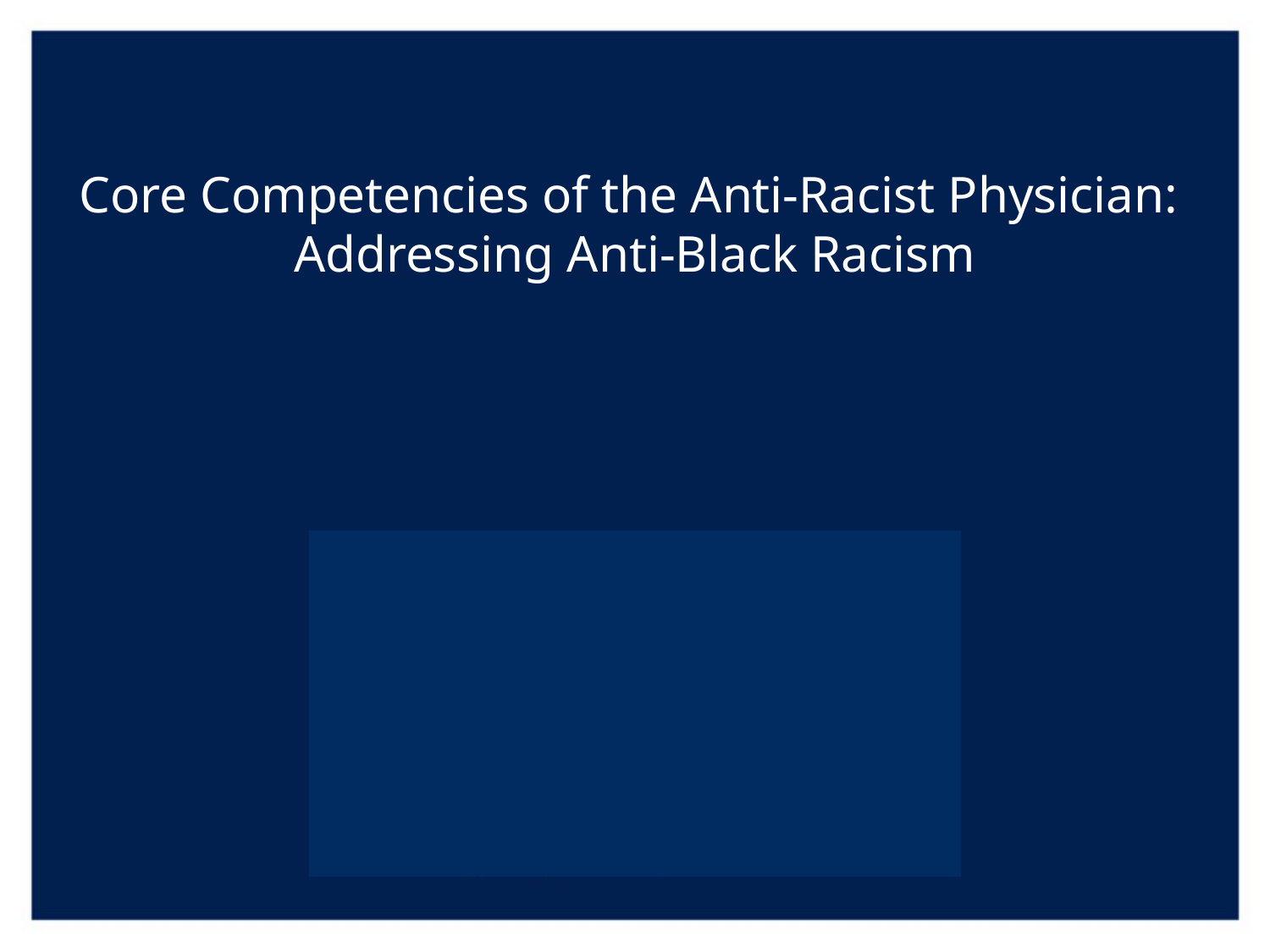

# Core Competencies of the Anti-Racist Physician: Addressing Anti-Black Racism

## Slide 2
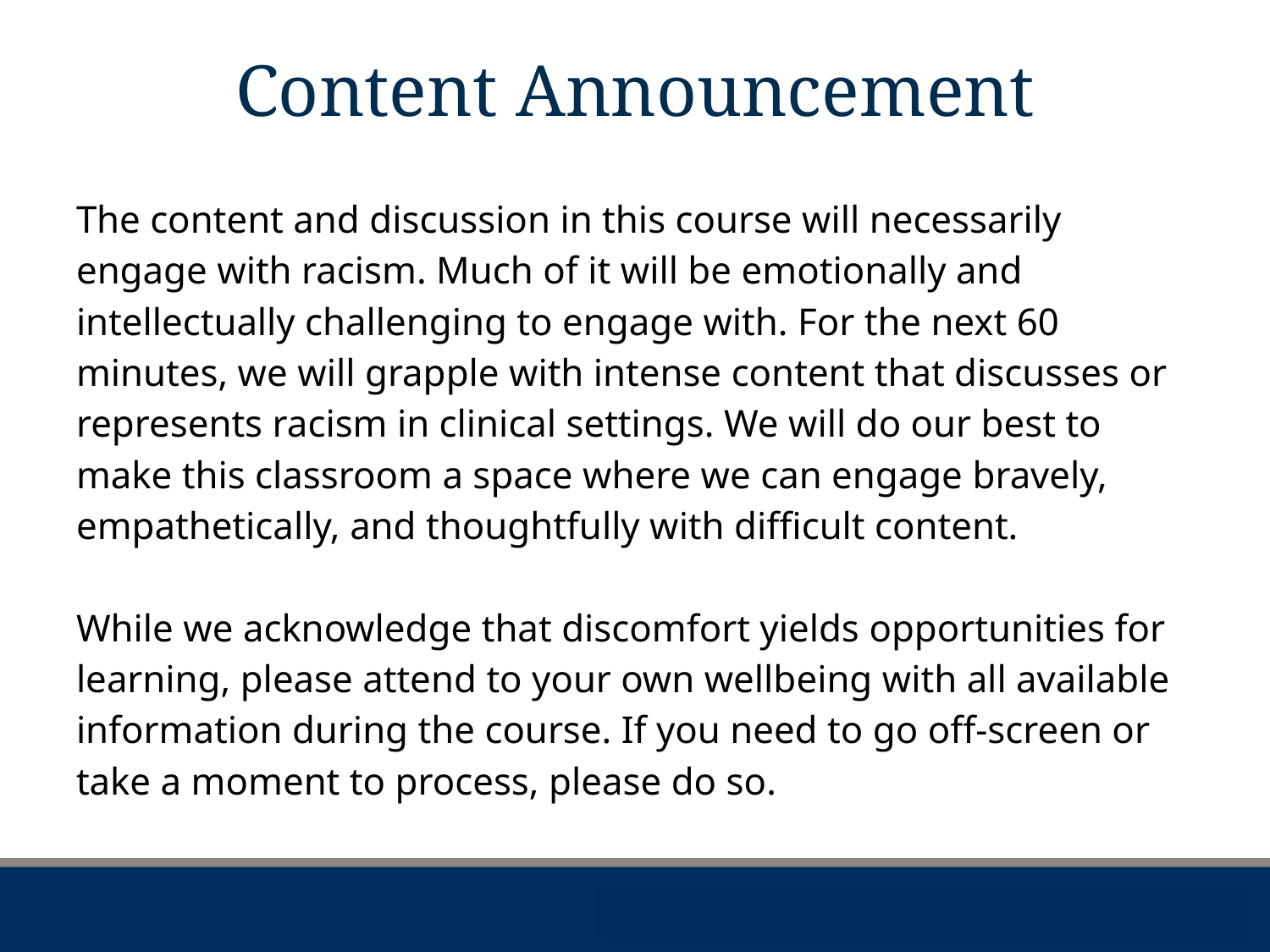

# Content Announcement
The content and discussion in this course will necessarily engage with racism. Much of it will be emotionally and intellectually challenging to engage with. For the next 60 minutes, we will grapple with intense content that discusses or represents racism in clinical settings. We will do our best to make this classroom a space where we can engage bravely, empathetically, and thoughtfully with difficult content.
While we acknowledge that discomfort yields opportunities for learning, please attend to your own wellbeing with all available information during the course. If you need to go off-screen or take a moment to process, please do so.

## Slide 3
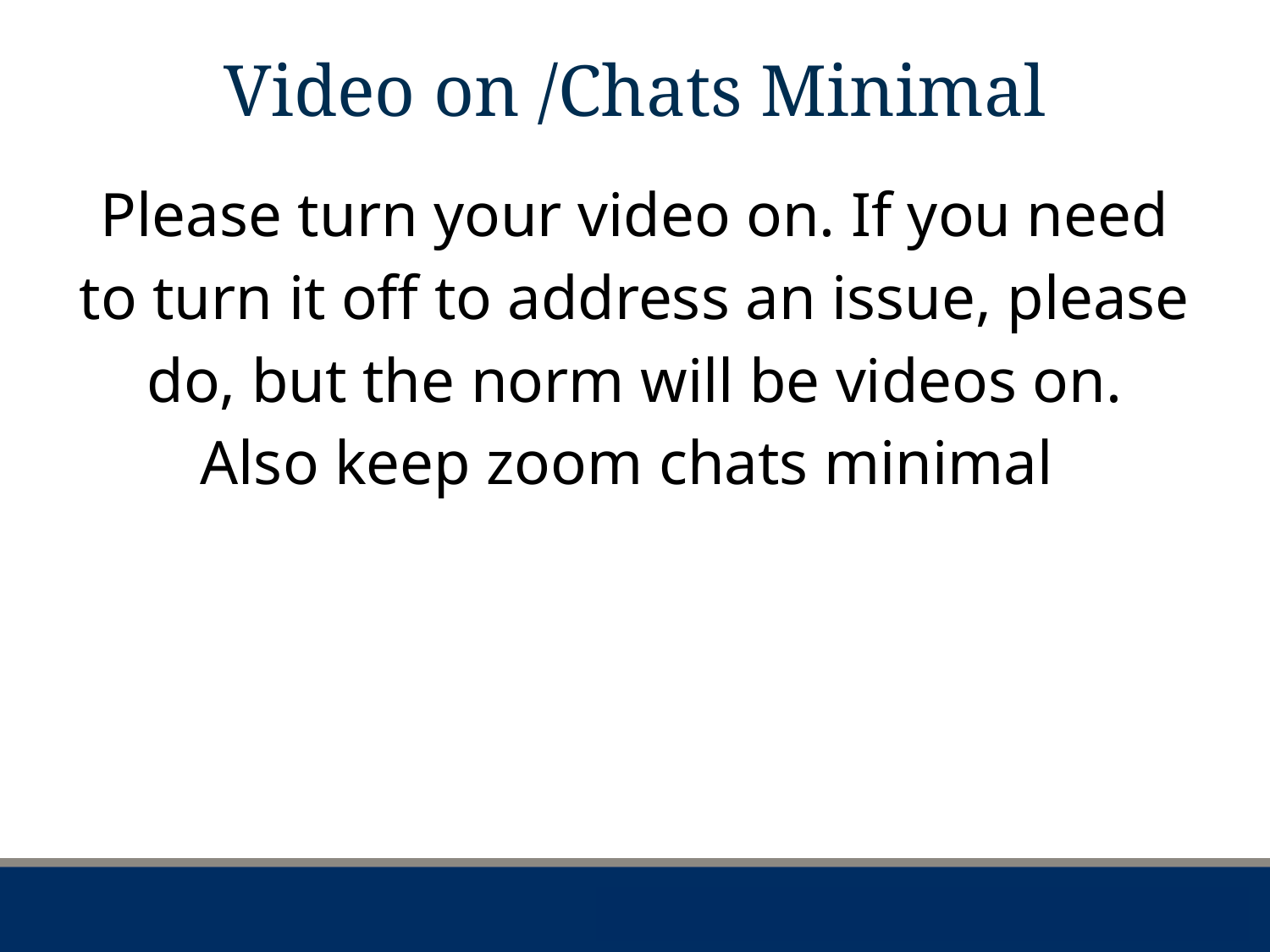

# Video on /Chats Minimal
Please turn your video on. If you need to turn it off to address an issue, please do, but the norm will be videos on.
Also keep zoom chats minimal

## Slide 4
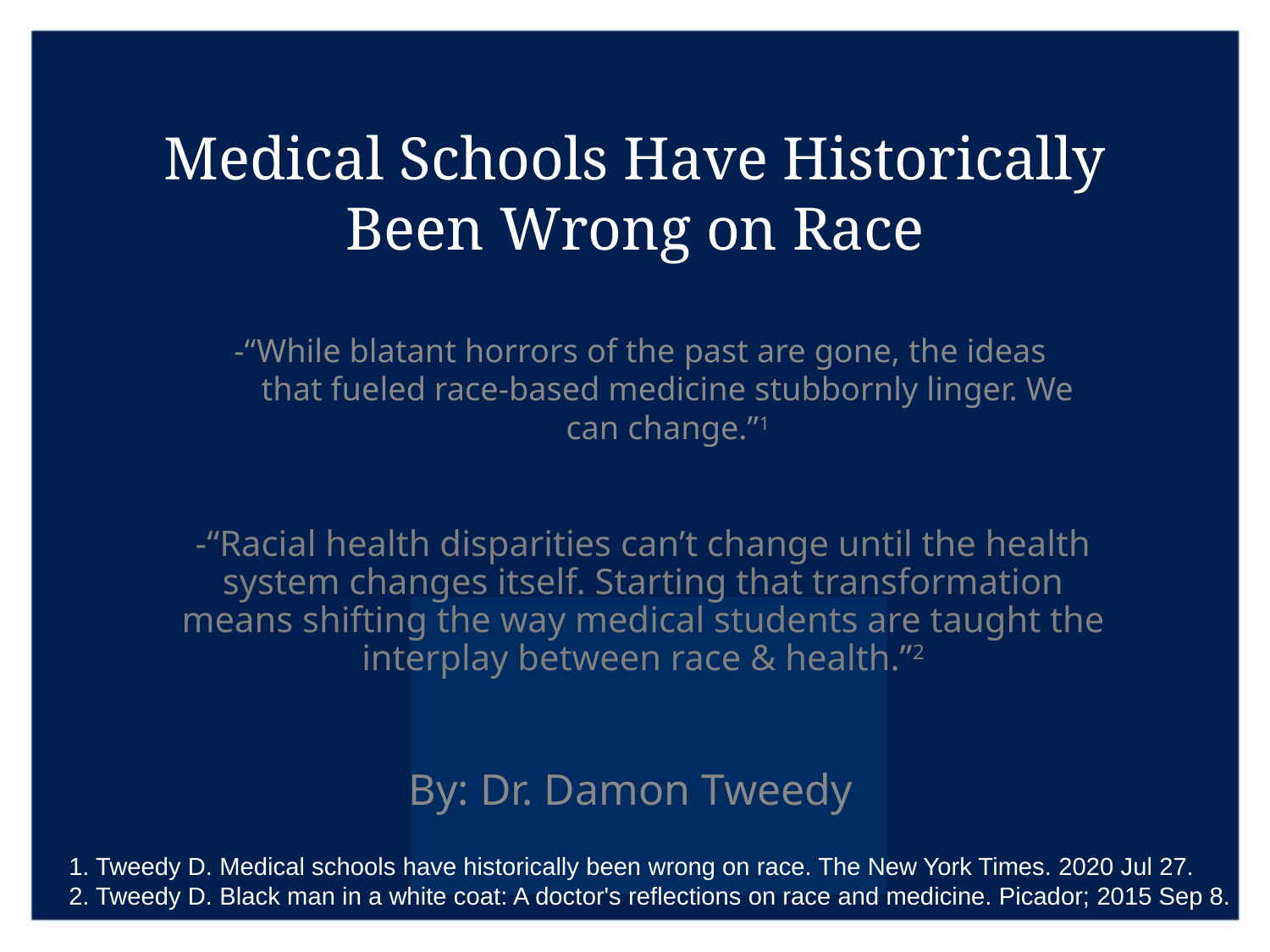

# Medical Schools Have Historically Been Wrong on Race
-“While blatant horrors of the past are gone, the ideas that fueled race-based medicine stubbornly linger. We can change.”1
-“Racial health disparities can’t change until the health system changes itself. Starting that transformation means shifting the way medical students are taught the interplay between race & health.”2
By: Dr. Damon Tweedy
1. Tweedy D. Medical schools have historically been wrong on race. The New York Times. 2020 Jul 27.
2. Tweedy D. Black man in a white coat: A doctor's reflections on race and medicine. Picador; 2015 Sep 8.

## Slide 5
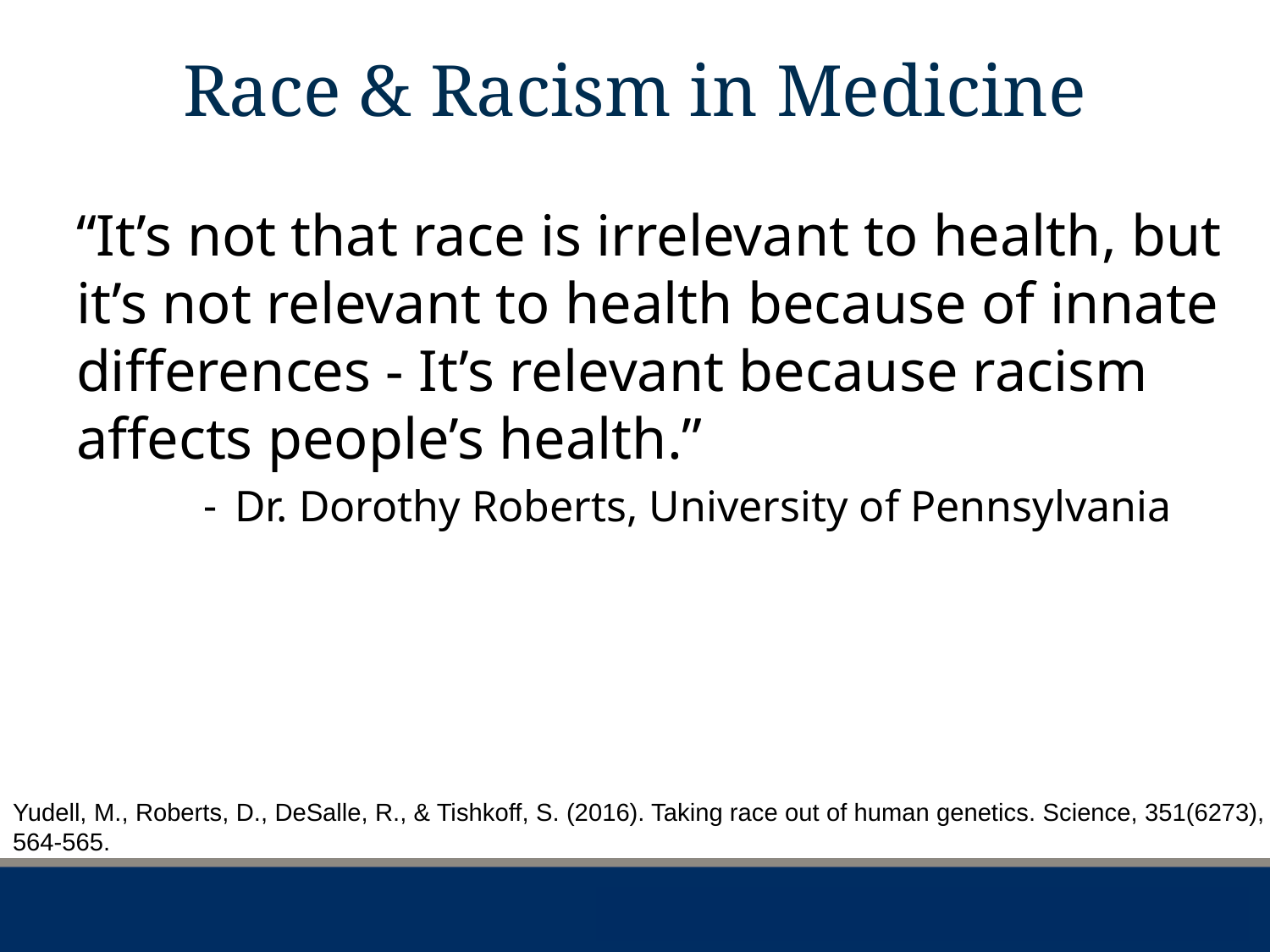

# Race & Racism in Medicine
“It’s not that race is irrelevant to health, but it’s not relevant to health because of innate differences - It’s relevant because racism affects people’s health.”
Dr. Dorothy Roberts, University of Pennsylvania
Yudell, M., Roberts, D., DeSalle, R., & Tishkoff, S. (2016). Taking race out of human genetics. Science, 351(6273), 564-565.

## Slide 6
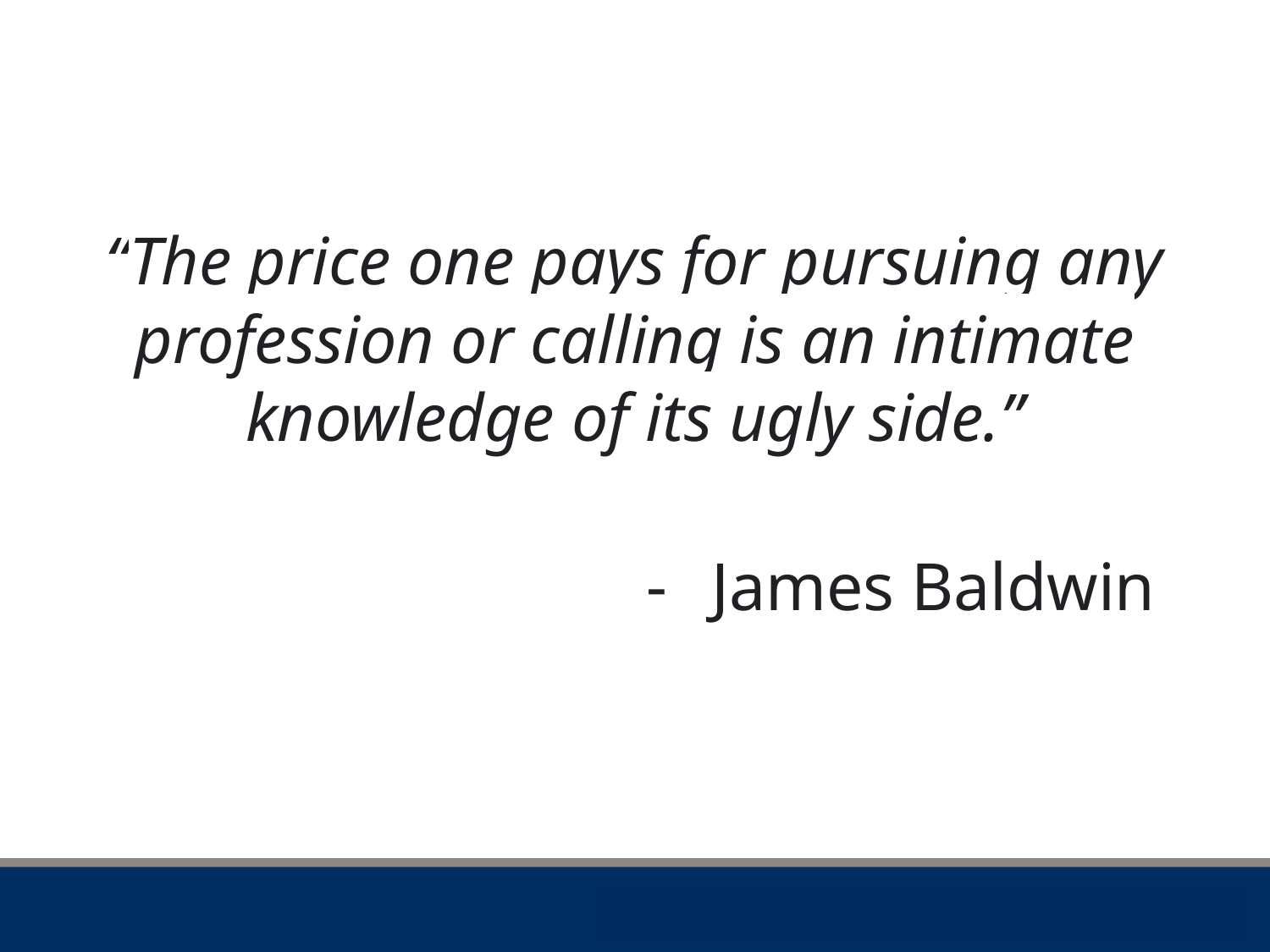

“The price one pays for pursuing any profession or calling is an intimate knowledge of its ugly side.”
James Baldwin

## Slide 7
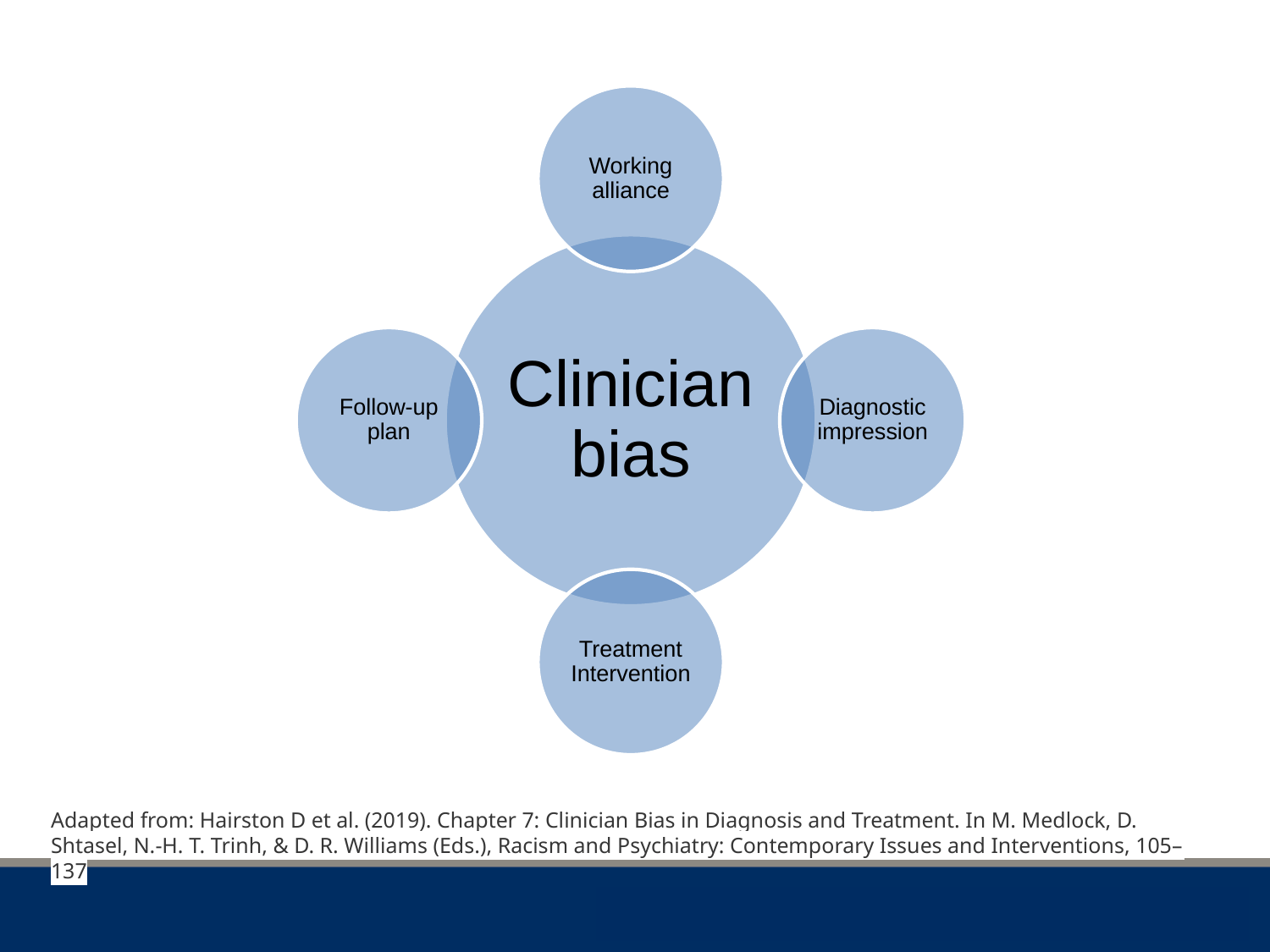

Working alliance
Clinician bias
Follow-up plan
Diagnostic impression
Treatment Intervention
Adapted from: Hairston D et al. (2019). Chapter 7: Clinician Bias in Diagnosis and Treatment. In M. Medlock, D. Shtasel, N.-H. T. Trinh, & D. R. Williams (Eds.), Racism and Psychiatry: Contemporary Issues and Interventions, 105–137

## Slide 8
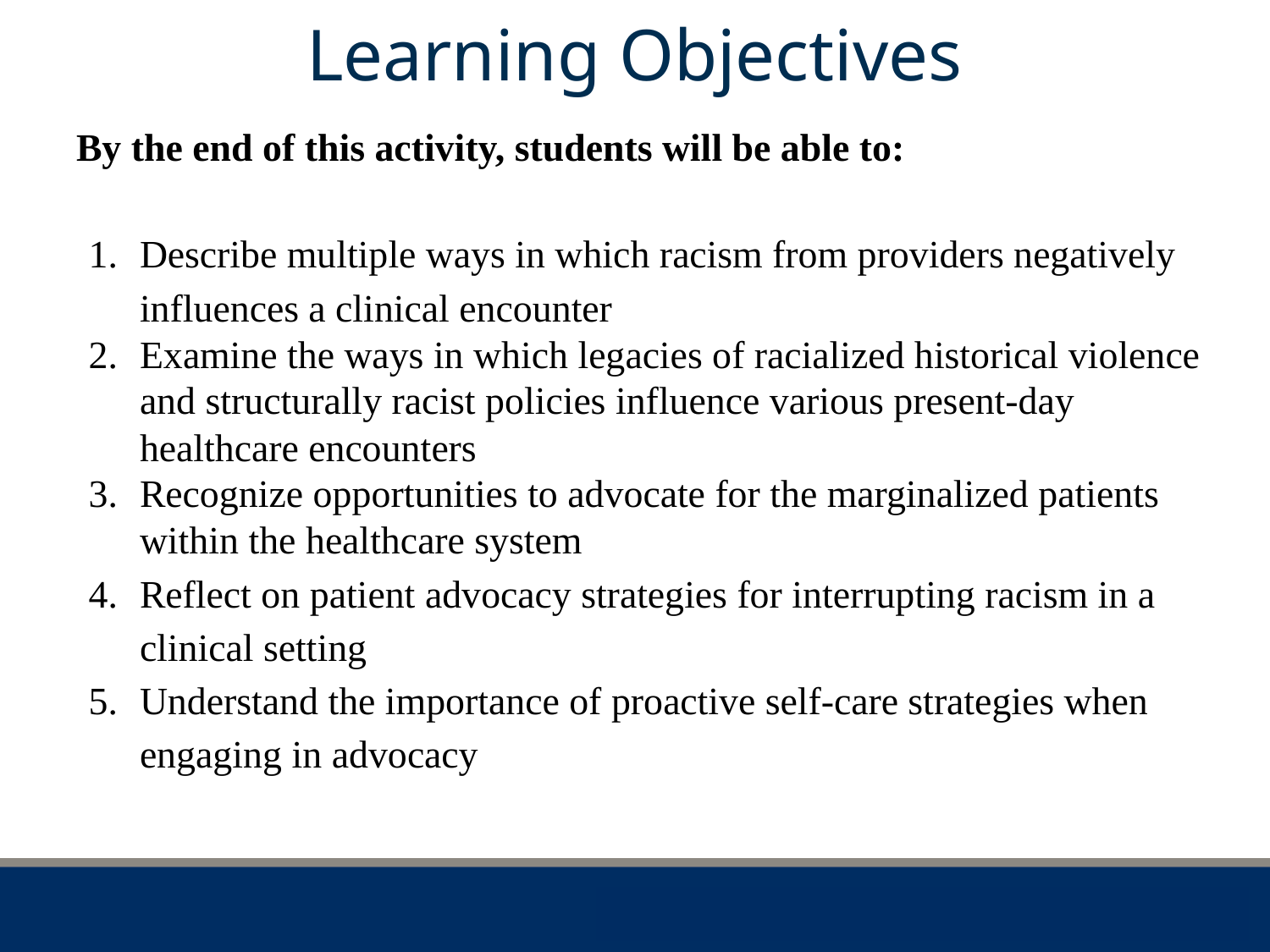

# Learning Objectives
By the end of this activity, students will be able to:
Describe multiple ways in which racism from providers negatively influences a clinical encounter
Examine the ways in which legacies of racialized historical violence and structurally racist policies influence various present-day healthcare encounters
Recognize opportunities to advocate for the marginalized patients within the healthcare system
Reflect on patient advocacy strategies for interrupting racism in a clinical setting
Understand the importance of proactive self-care strategies when engaging in advocacy

## Slide 9
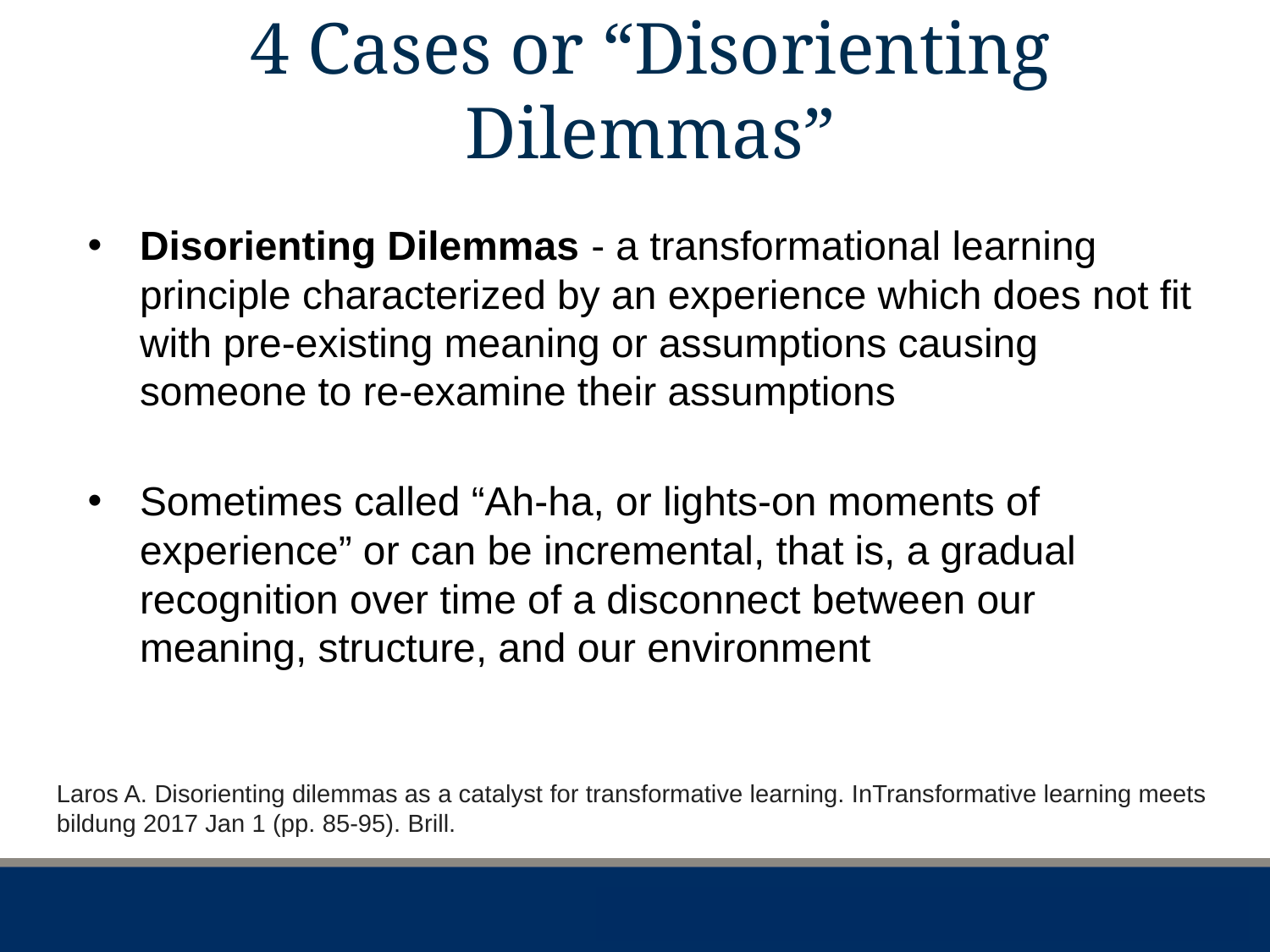

# 4 Cases or “Disorienting Dilemmas”
Disorienting Dilemmas - a transformational learning principle characterized by an experience which does not fit with pre-existing meaning or assumptions causing someone to re-examine their assumptions
Sometimes called “Ah-ha, or lights-on moments of experience” or can be incremental, that is, a gradual recognition over time of a disconnect between our meaning, structure, and our environment
Laros A. Disorienting dilemmas as a catalyst for transformative learning. InTransformative learning meets bildung 2017 Jan 1 (pp. 85-95). Brill.

## Slide 10
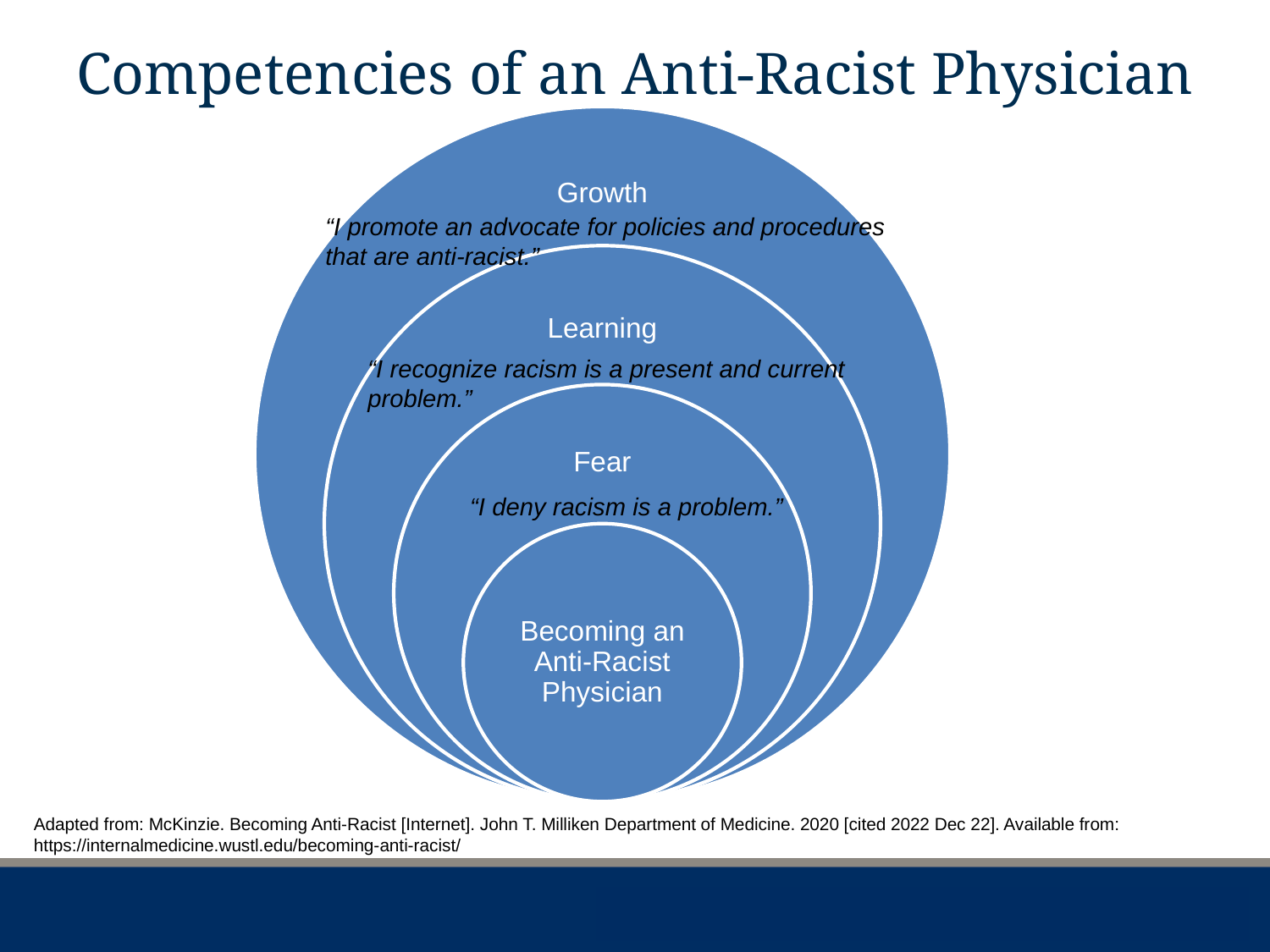

# Competencies of an Anti-Racist Physician
Growth
Learning
Fear
Becoming an Anti-Racist Physician
“I promote an advocate for policies and procedures that are anti-racist.”
“I recognize racism is a present and current problem.”
“I deny racism is a problem.”
Adapted from: McKinzie. Becoming Anti-Racist [Internet]. John T. Milliken Department of Medicine. 2020 [cited 2022 Dec 22]. Available from: https://internalmedicine.wustl.edu/becoming-anti-racist/

## Slide 11
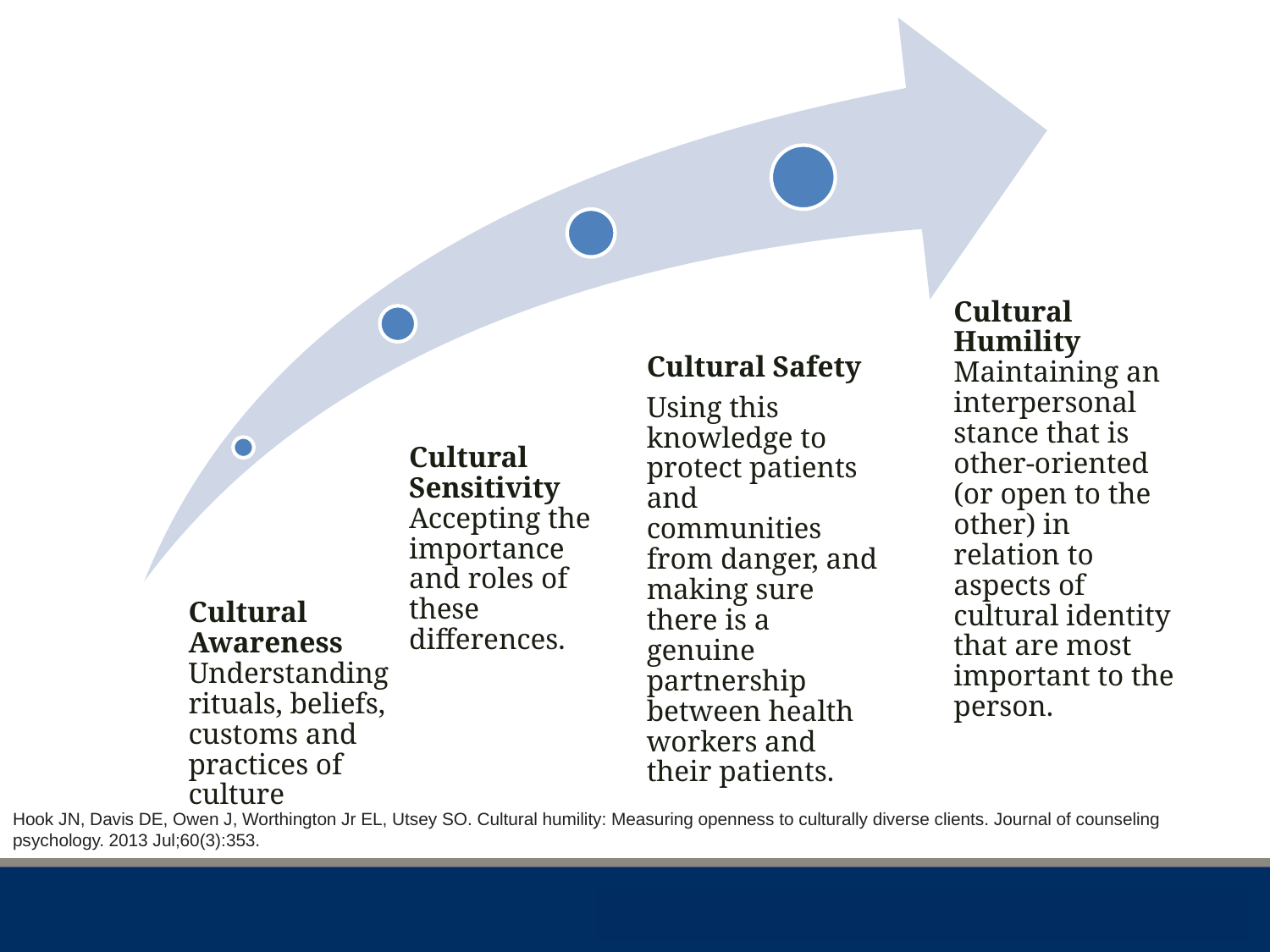

Cultural Humility Maintaining an interpersonal stance that is other-oriented (or open to the other) in relation to aspects of cultural identity that are most important to the person.
Cultural Safety
Using this knowledge to protect patients and communities from danger, and making sure there is a genuine partnership between health workers and their patients.
Cultural Sensitivity Accepting the importance and roles of these differences.
Cultural Awareness Understanding rituals, beliefs, customs and practices of culture
Hook JN, Davis DE, Owen J, Worthington Jr EL, Utsey SO. Cultural humility: Measuring openness to culturally diverse clients. Journal of counseling psychology. 2013 Jul;60(3):353.
‹#›

## Slide 12
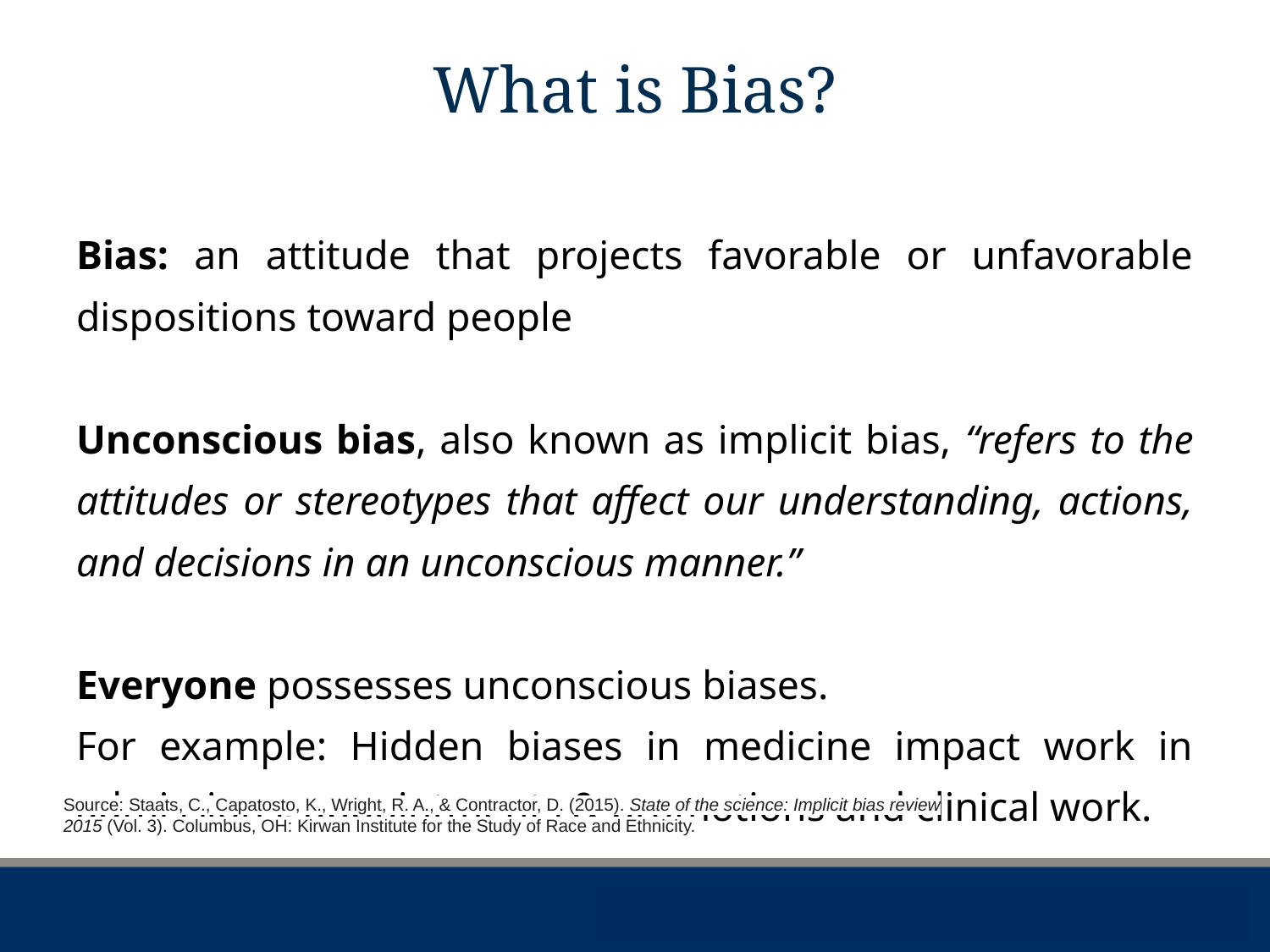

# What is Bias?
Bias: an attitude that projects favorable or unfavorable dispositions toward people
Unconscious bias, also known as implicit bias, “refers to the attitudes or stereotypes that affect our understanding, actions, and decisions in an unconscious manner.”
Everyone possesses unconscious biases.
For example: Hidden biases in medicine impact work in admissions, appointments & promotions and clinical work.
Source: Staats, C., Capatosto, K., Wright, R. A., & Contractor, D. (2015). State of the science: Implicit bias review 2015 (Vol. 3). Columbus, OH: Kirwan Institute for the Study of Race and Ethnicity.

## Slide 13
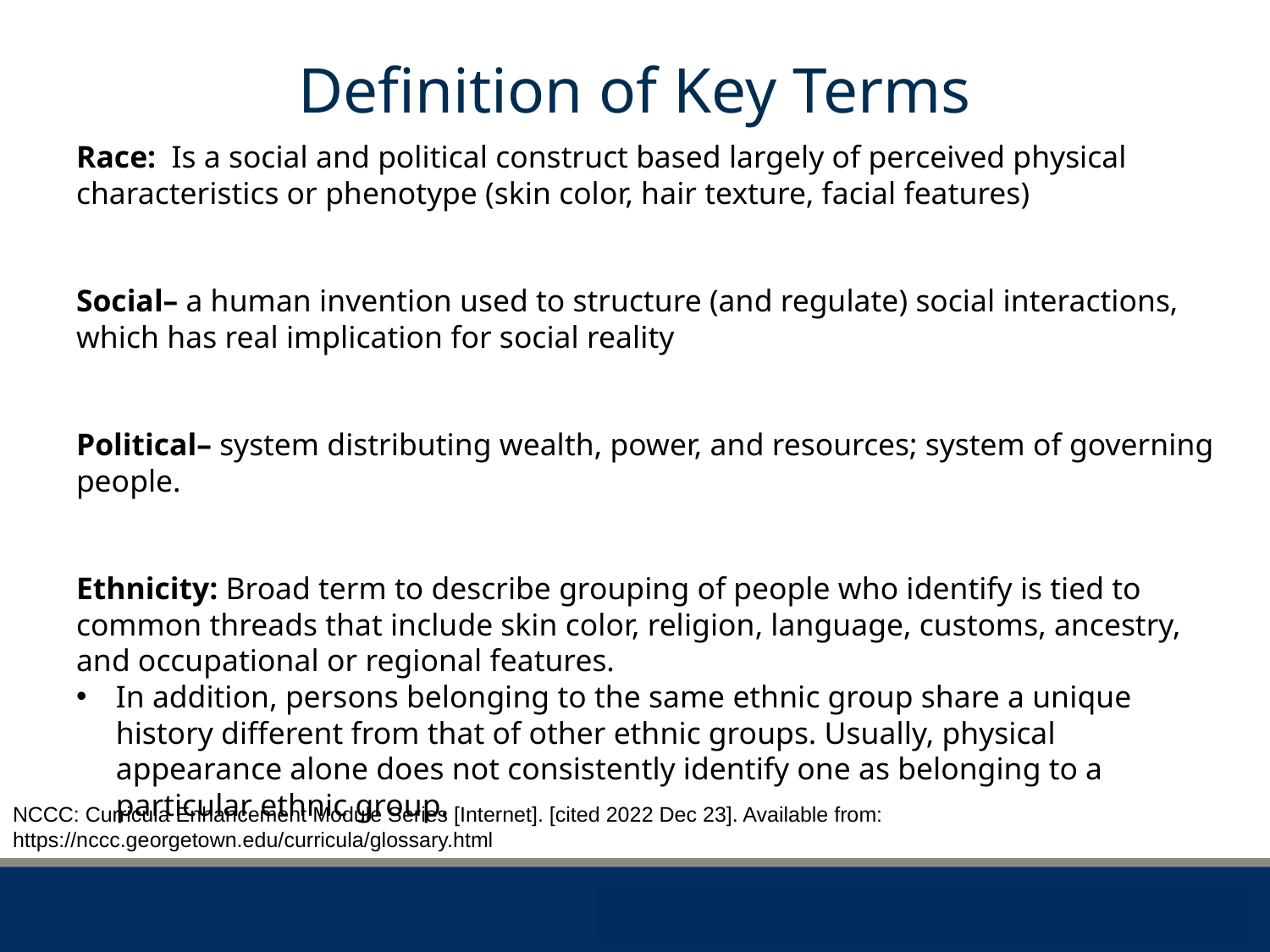

Definition of Key Terms
Race:  Is a social and political construct based largely of perceived physical characteristics or phenotype (skin color, hair texture, facial features)
Social– a human invention used to structure (and regulate) social interactions, which has real implication for social reality
Political– system distributing wealth, power, and resources; system of governing
people.
Ethnicity: Broad term to describe grouping of people who identify is tied to common threads that include skin color, religion, language, customs, ancestry, and occupational or regional features.
In addition, persons belonging to the same ethnic group share a unique history different from that of other ethnic groups. Usually, physical appearance alone does not consistently identify one as belonging to a particular ethnic group.
NCCC: Curricula Enhancement Module Series [Internet]. [cited 2022 Dec 23]. Available from: https://nccc.georgetown.edu/curricula/glossary.html

## Slide 14
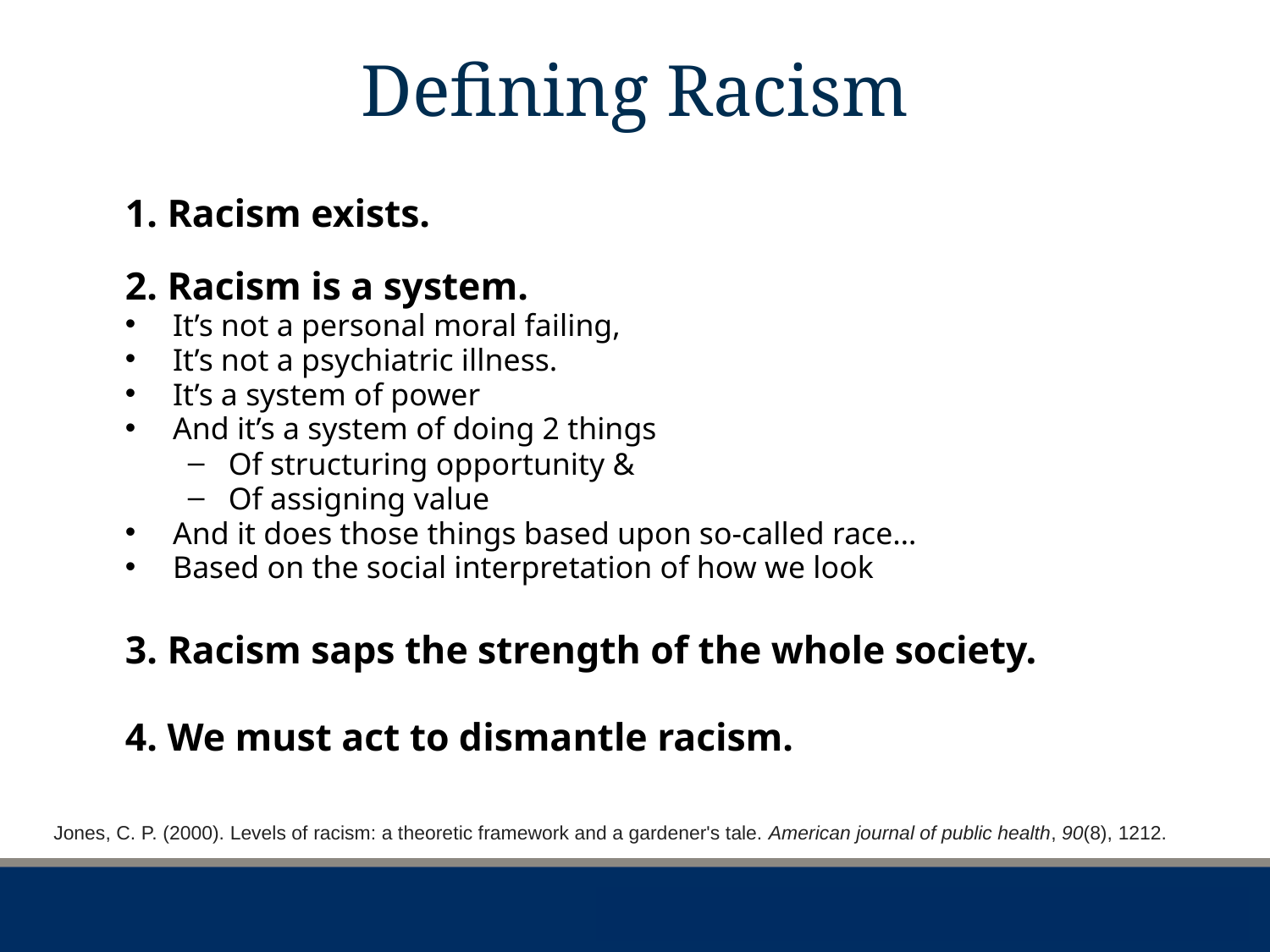

# Defining Racism
1. Racism exists.
2. Racism is a system.
It’s not a personal moral failing,
It’s not a psychiatric illness.
It’s a system of power
And it’s a system of doing 2 things
Of structuring opportunity &
Of assigning value
And it does those things based upon so-called race…
Based on the social interpretation of how we look
3. Racism saps the strength of the whole society.
4. We must act to dismantle racism.
Jones, C. P. (2000). Levels of racism: a theoretic framework and a gardener's tale. American journal of public health, 90(8), 1212.

## Slide 15
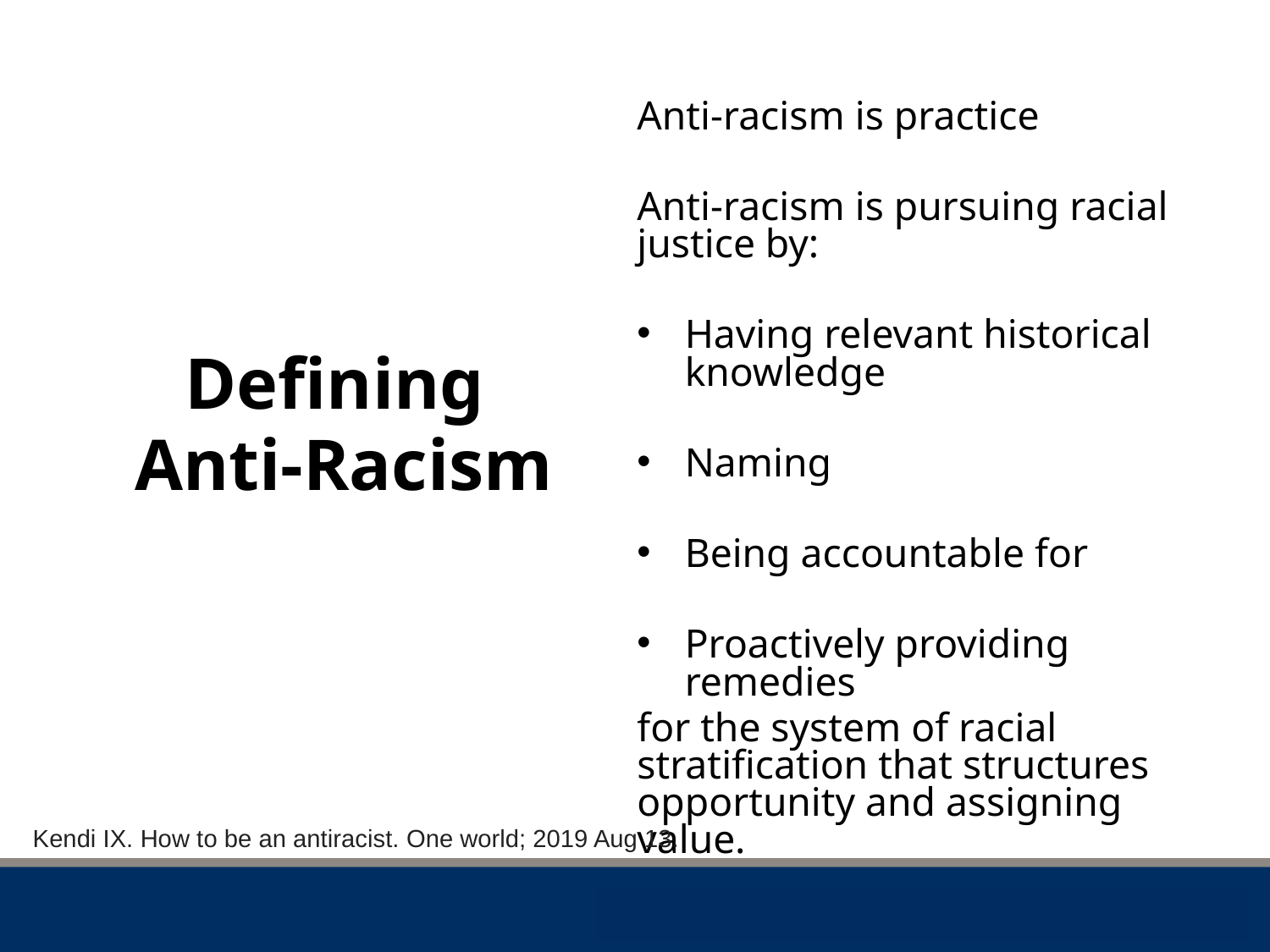

Anti-racism is practice
Anti-racism is pursuing racial justice by:
Having relevant historical knowledge
Naming
Being accountable for
Proactively providing remedies
for the system of racial stratification that structures opportunity and assigning value.
Defining
Anti-Racism
Kendi IX. How to be an antiracist. One world; 2019 Aug 13.

## Slide 16
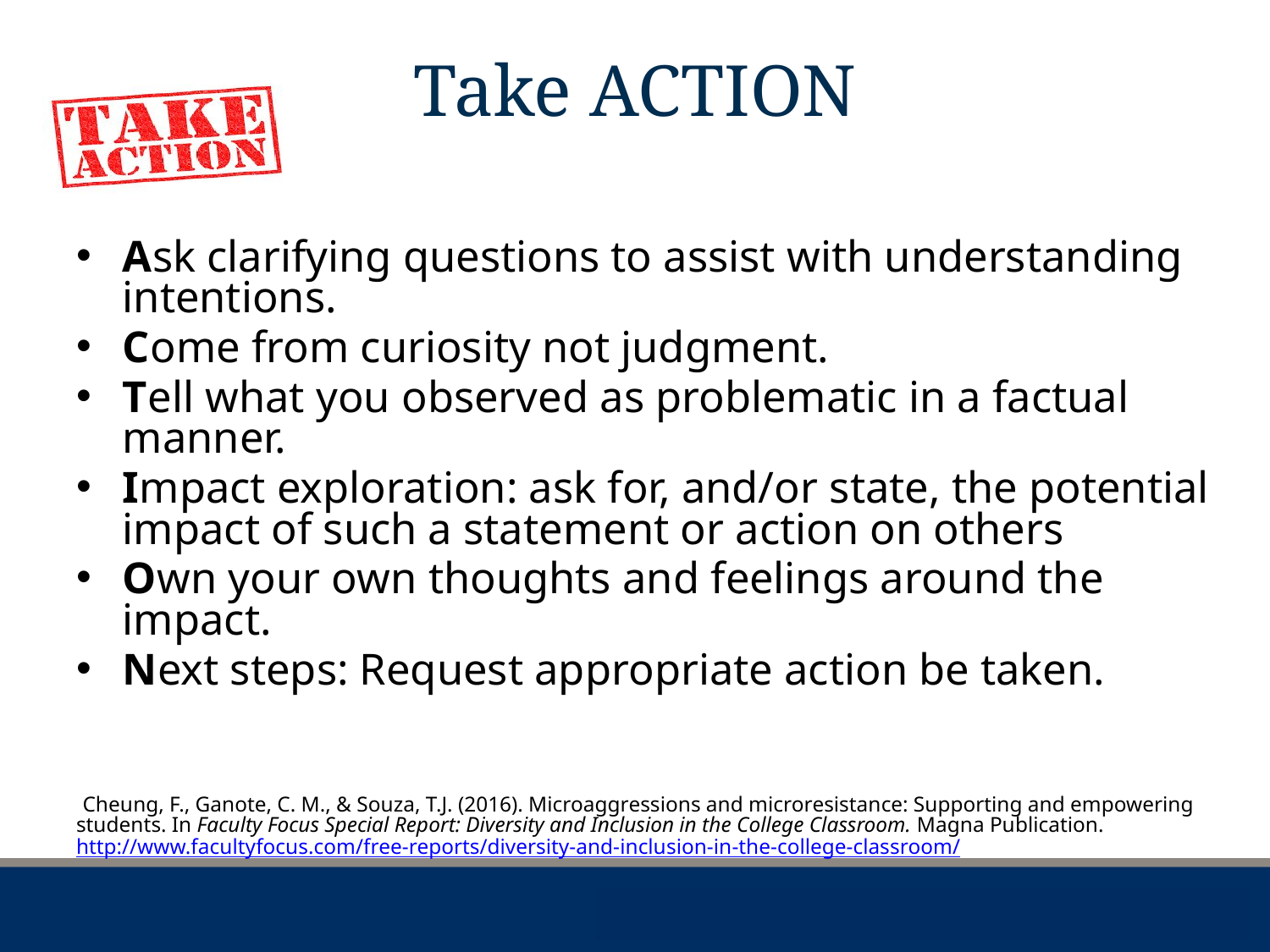

# Take ACTION
Ask clarifying questions to assist with understanding intentions.
Come from curiosity not judgment.
Tell what you observed as problematic in a factual manner.
Impact exploration: ask for, and/or state, the potential impact of such a statement or action on others
Own your own thoughts and feelings around the impact.
Next steps: Request appropriate action be taken.
 Cheung, F., Ganote, C. M., & Souza, T.J. (2016). Microaggressions and microresistance: Supporting and empowering students. In Faculty Focus Special Report: Diversity and Inclusion in the College Classroom. Magna Publication. http://www.facultyfocus.com/free-reports/diversity-and-inclusion-in-the-college-classroom/

## Slide 17
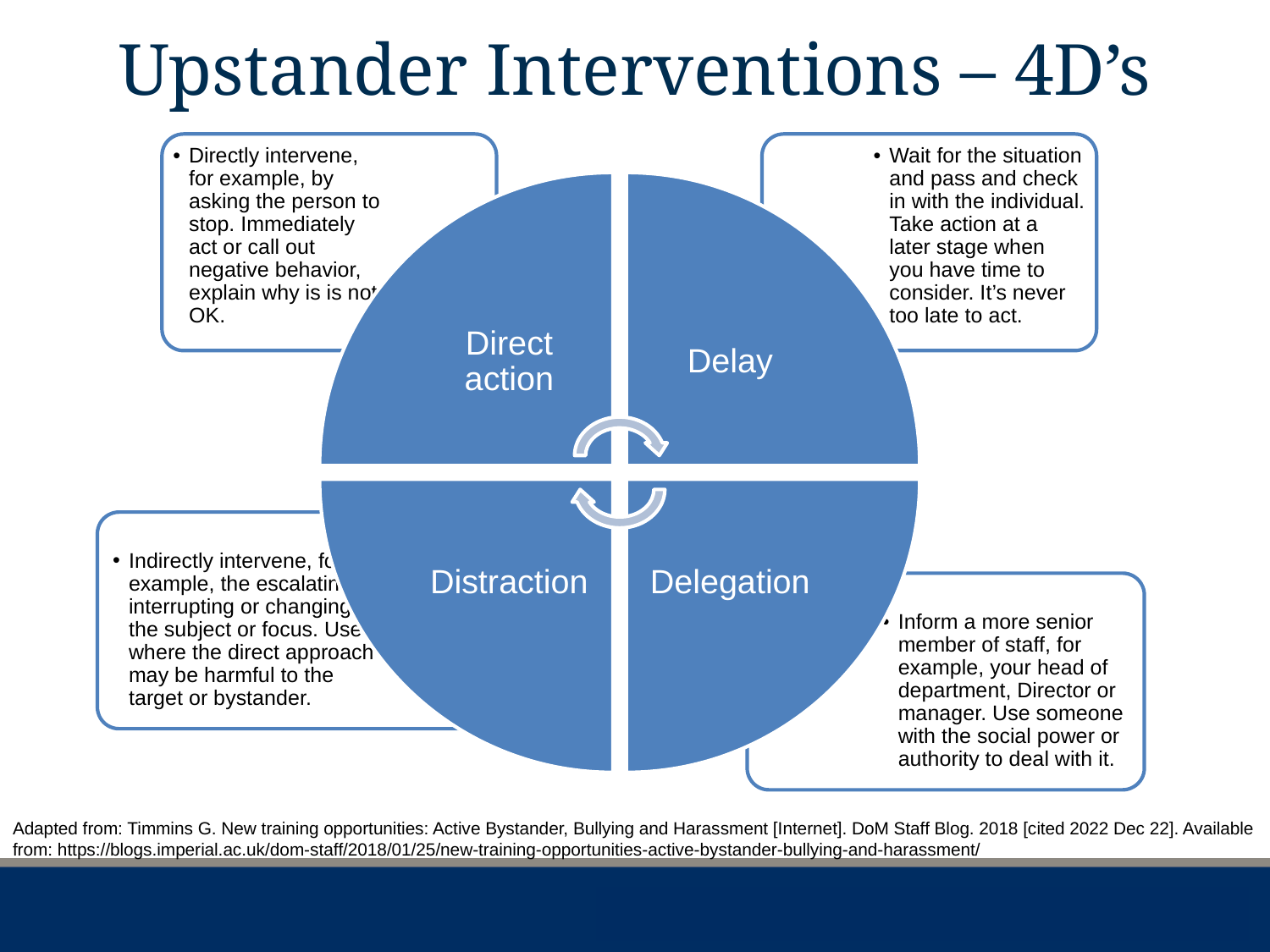

# Upstander Interventions – 4D’s
Directly intervene, for example, by asking the person to stop. Immediately act or call out negative behavior, explain why is is not OK.
Wait for the situation and pass and check in with the individual. Take action at a later stage when you have time to consider. It’s never too late to act.
Direct action
Delay
Distraction
Delegation
Indirectly intervene, for example, the escalating by interrupting or changing the subject or focus. Useful where the direct approach may be harmful to the target or bystander.
Inform a more senior member of staff, for example, your head of department, Director or manager. Use someone with the social power or authority to deal with it.
Adapted from: Timmins G. New training opportunities: Active Bystander, Bullying and Harassment [Internet]. DoM Staff Blog. 2018 [cited 2022 Dec 22]. Available from: https://blogs.imperial.ac.uk/dom-staff/2018/01/25/new-training-opportunities-active-bystander-bullying-and-harassment/

## Slide 18
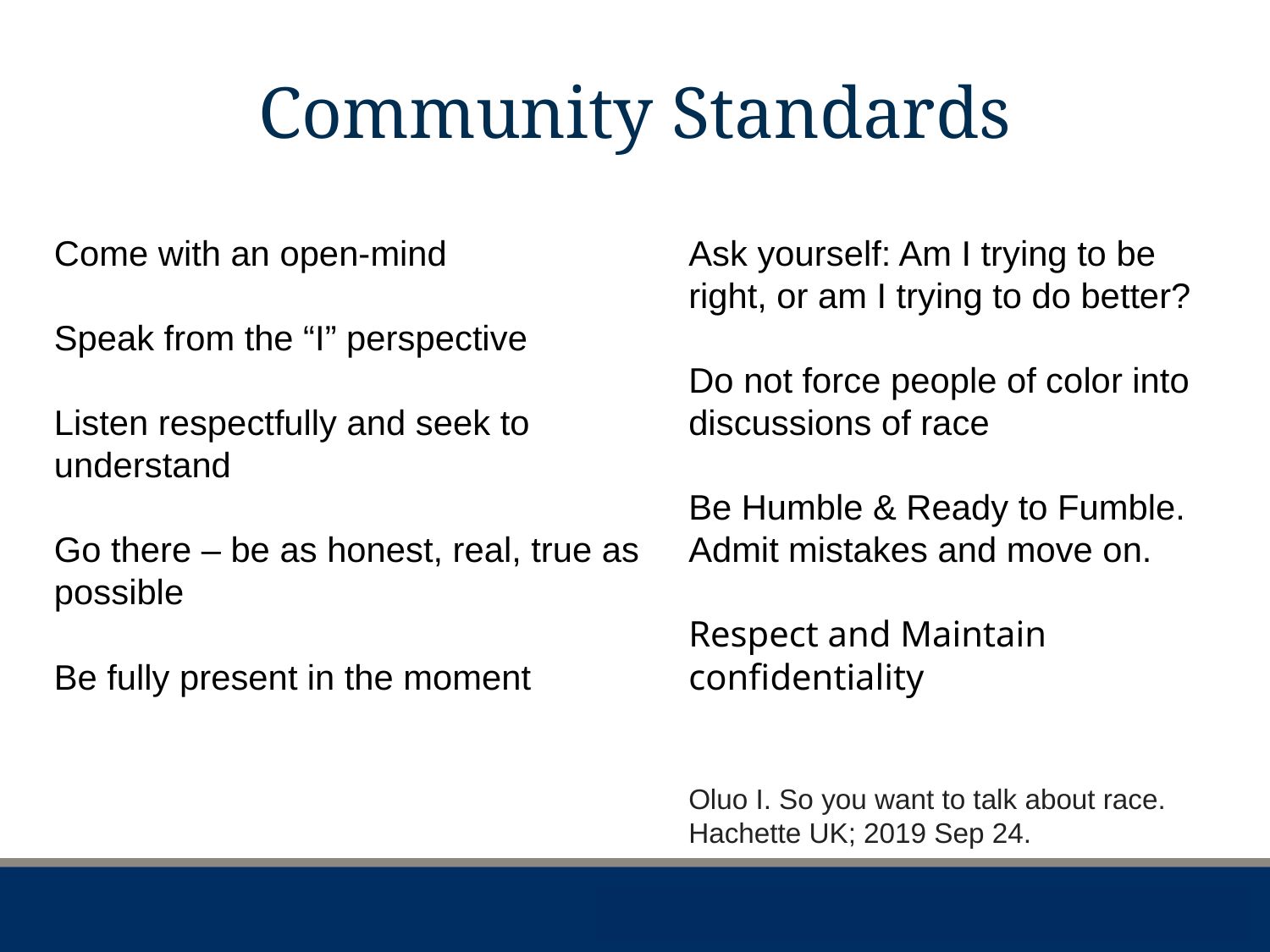

# Community Standards
Ask yourself: Am I trying to be right, or am I trying to do better?
Do not force people of color into discussions of race
Be Humble & Ready to Fumble. Admit mistakes and move on.
Respect and Maintain confidentiality
Oluo I. So you want to talk about race. Hachette UK; 2019 Sep 24.
Come with an open-mind
Speak from the “I” perspective
Listen respectfully and seek to understand
Go there – be as honest, real, true as possible
Be fully present in the moment
