# Supplementary material for: Core Competencies of an Anti-racist Physician: Elective Course for Undergraduate Medical Students
Source: MedEdPORTAL. 2024 May 14;20:11395. doi: 10.15766/mep_2374-8265.11395 (PMC11219086; doi:10.15766/mep_2374-8265.11395)
Supplement: Supplementary file 1 — Disorienting Dilemmas.docxFacilitator Guidelines.docxPrework Module.docxOpening Slides.pptxFacilitator Slides.pptxClosing Remarks Slides.pptxExit Ticket.docxPre- and Postassessment.docx [file mep_2374-8265.11395-s001.zip › E. Facilitator Slides.pptx]

## Slide 1
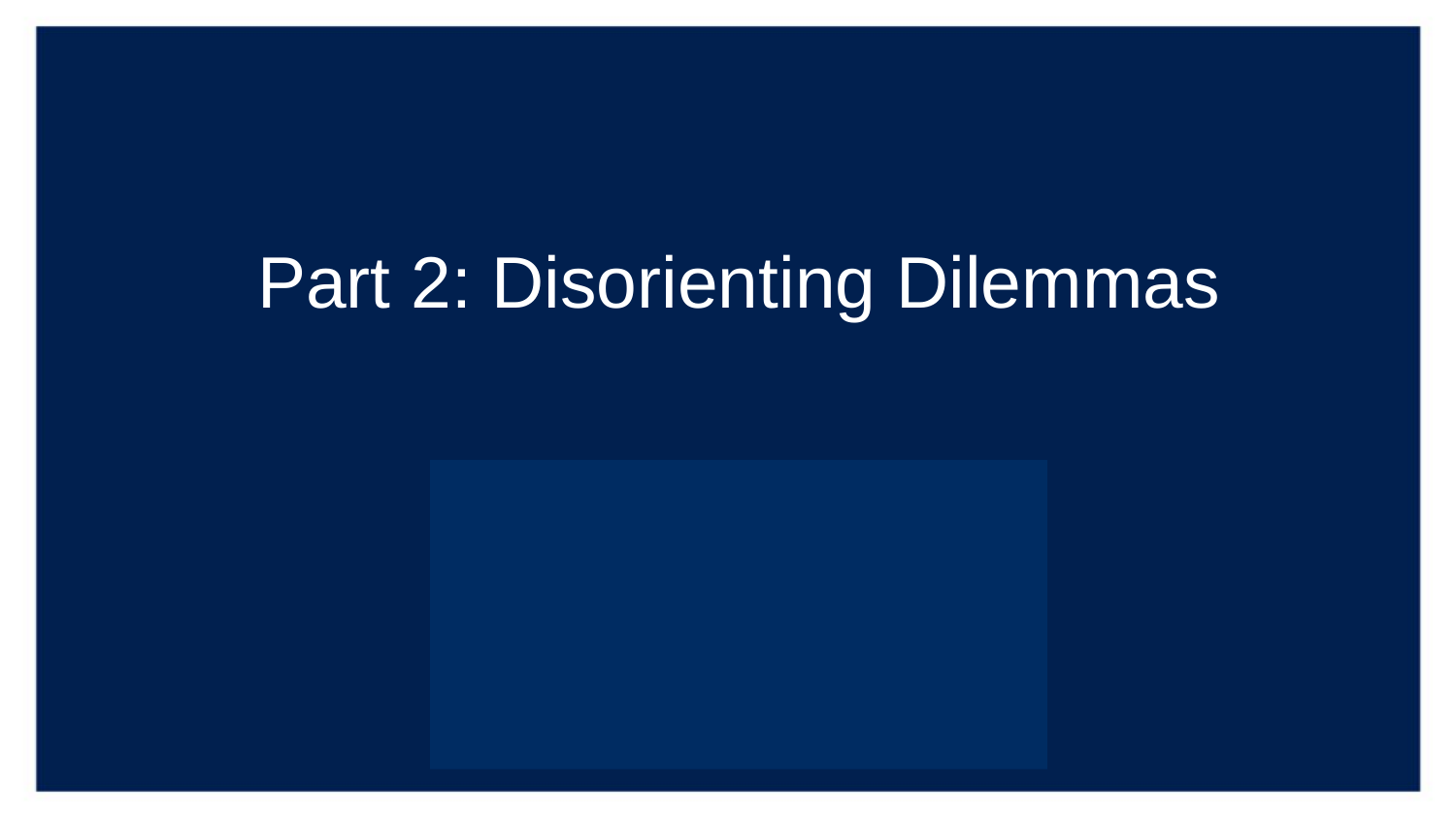

# Part 2: Disorienting Dilemmas

## Slide 2
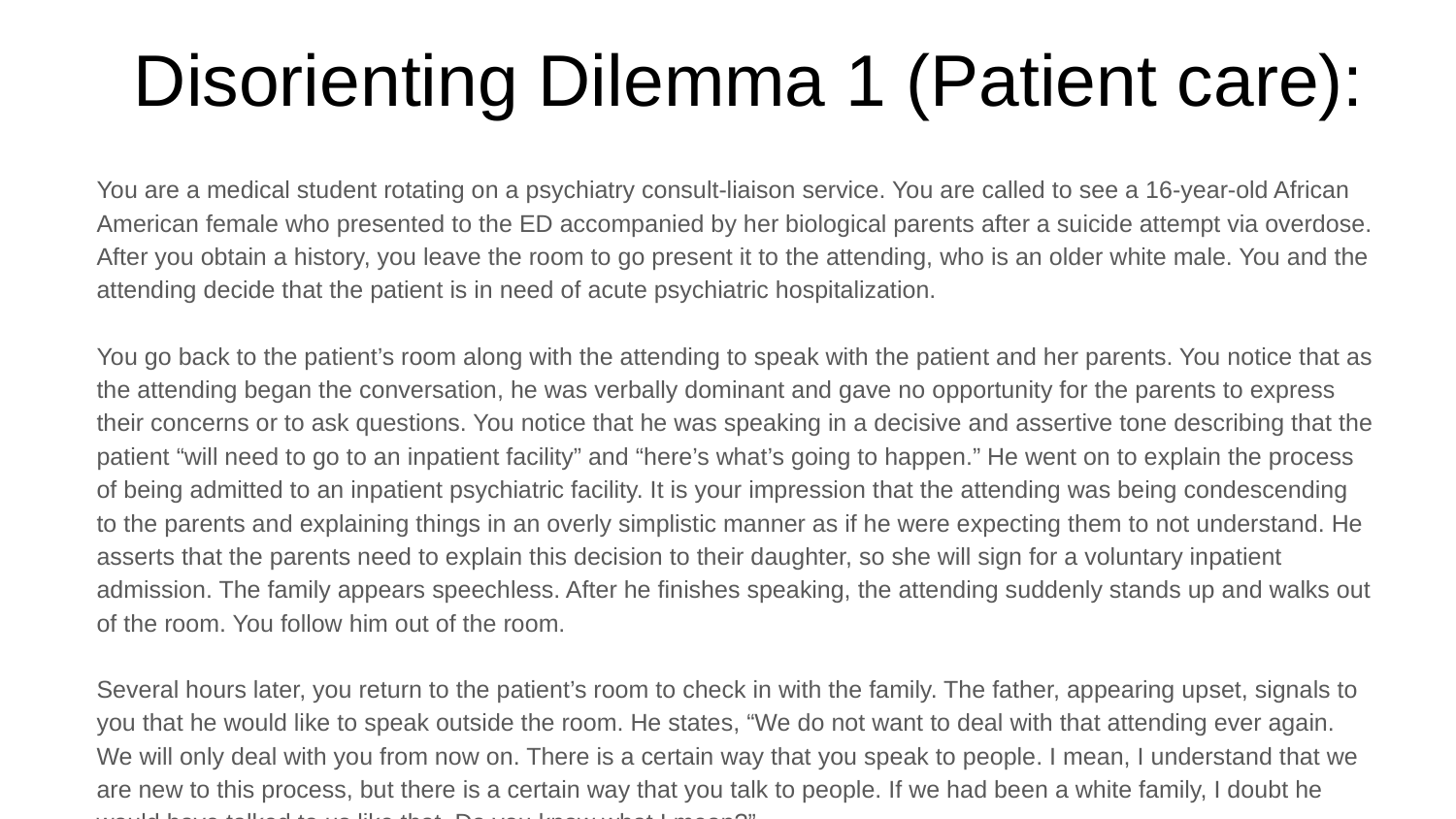

# Disorienting Dilemma 1 (Patient care):
You are a medical student rotating on a psychiatry consult-liaison service. You are called to see a 16-year-old African American female who presented to the ED accompanied by her biological parents after a suicide attempt via overdose. After you obtain a history, you leave the room to go present it to the attending, who is an older white male. You and the attending decide that the patient is in need of acute psychiatric hospitalization.
You go back to the patient’s room along with the attending to speak with the patient and her parents. You notice that as the attending began the conversation, he was verbally dominant and gave no opportunity for the parents to express their concerns or to ask questions. You notice that he was speaking in a decisive and assertive tone describing that the patient “will need to go to an inpatient facility” and “here’s what’s going to happen.” He went on to explain the process of being admitted to an inpatient psychiatric facility. It is your impression that the attending was being condescending to the parents and explaining things in an overly simplistic manner as if he were expecting them to not understand. He asserts that the parents need to explain this decision to their daughter, so she will sign for a voluntary inpatient admission. The family appears speechless. After he finishes speaking, the attending suddenly stands up and walks out of the room. You follow him out of the room.
Several hours later, you return to the patient’s room to check in with the family. The father, appearing upset, signals to you that he would like to speak outside the room. He states, “We do not want to deal with that attending ever again. We will only deal with you from now on. There is a certain way that you speak to people. I mean, I understand that we are new to this process, but there is a certain way that you talk to people. If we had been a white family, I doubt he would have talked to us like that. Do you know what I mean?”

## Slide 3
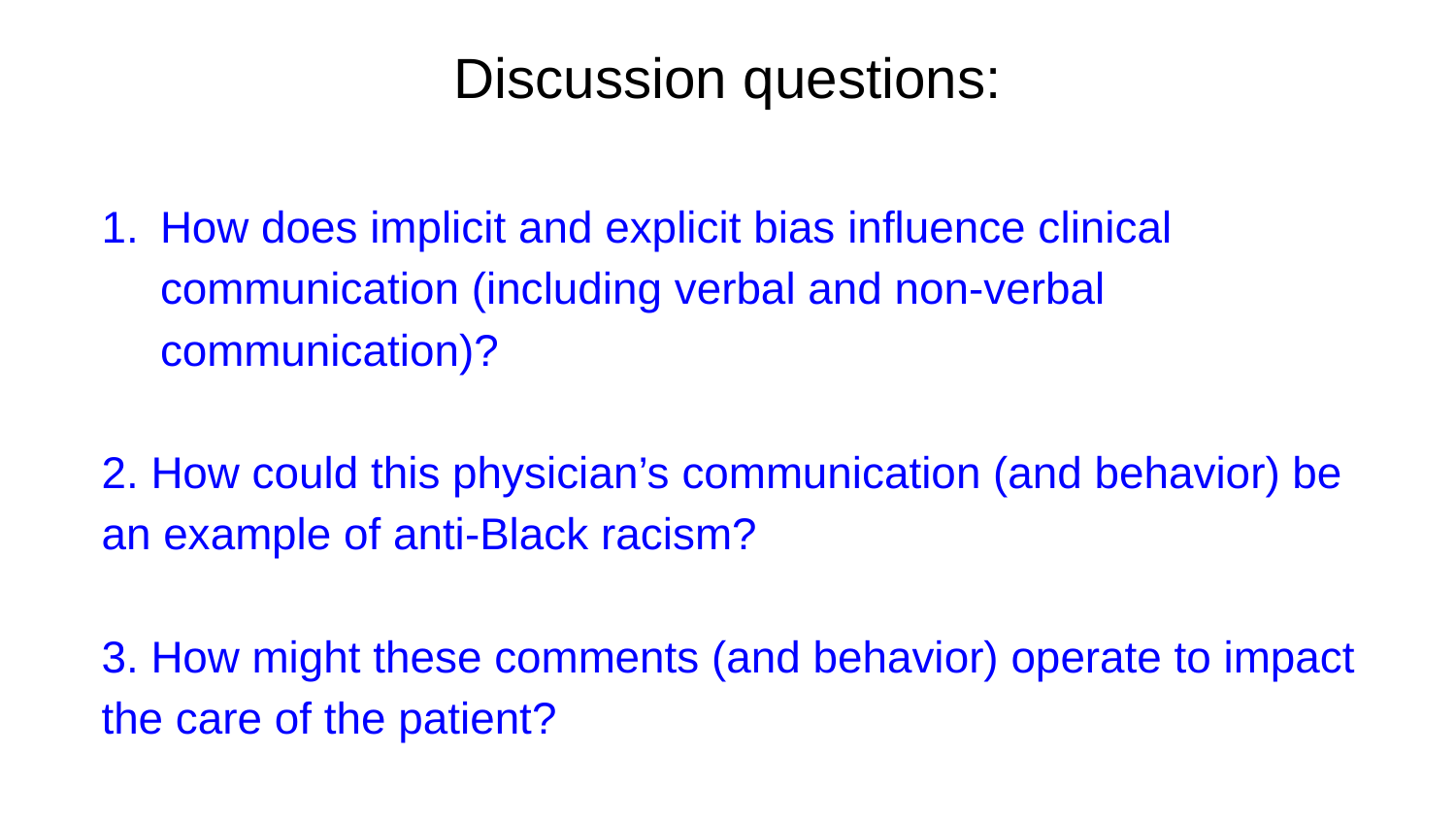

# Discussion questions:
How does implicit and explicit bias influence clinical communication (including verbal and non-verbal communication)?
2. How could this physician’s communication (and behavior) be an example of anti-Black racism?
3. How might these comments (and behavior) operate to impact the care of the patient?

## Slide 4
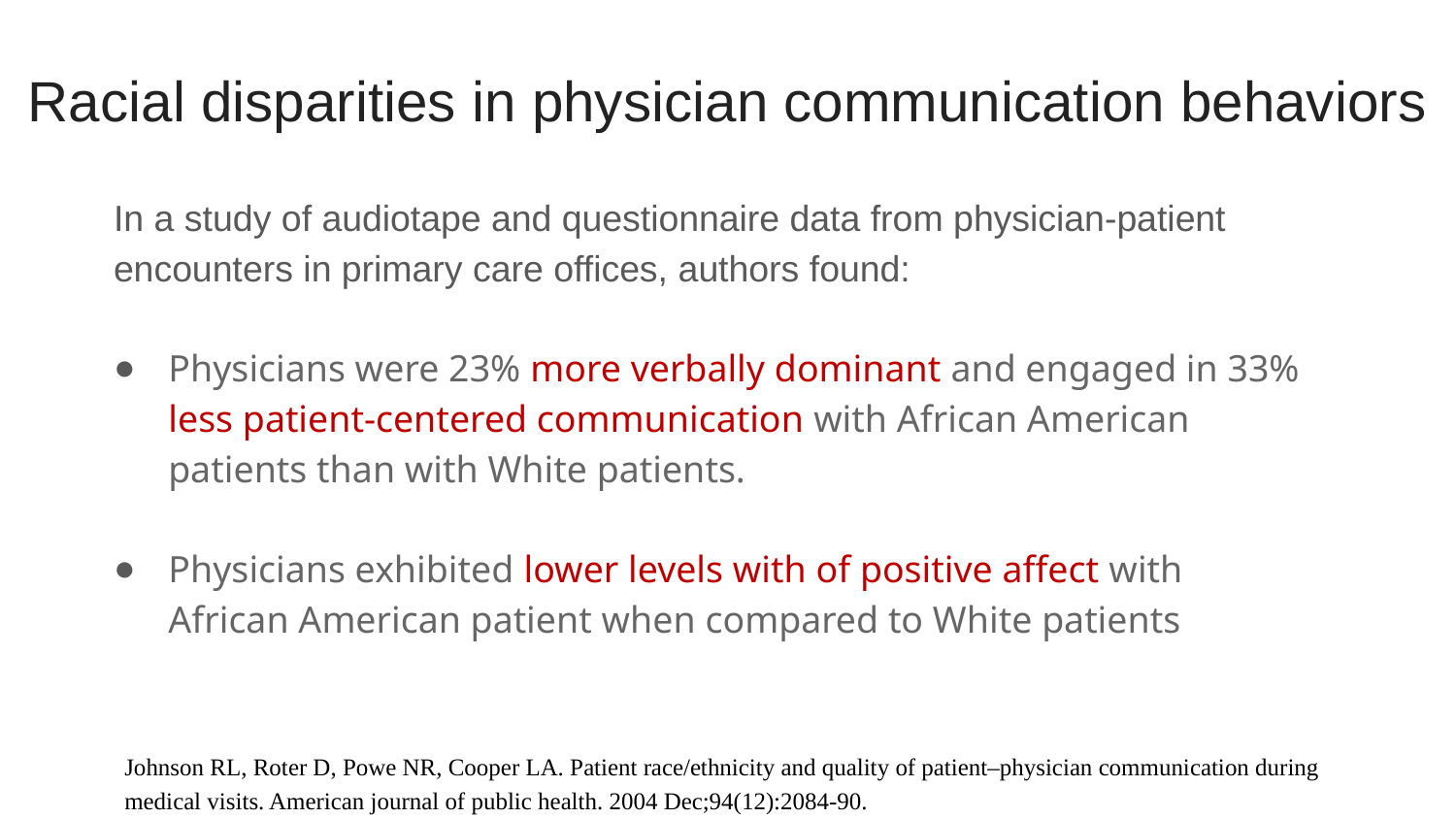

# Racial disparities in physician communication behaviors
In a study of audiotape and questionnaire data from physician-patient encounters in primary care offices, authors found:
Physicians were 23% more verbally dominant and engaged in 33% less patient-centered communication with African American patients than with White patients.
Physicians exhibited lower levels with of positive affect with African American patient when compared to White patients
Johnson RL, Roter D, Powe NR, Cooper LA. Patient race/ethnicity and quality of patient–physician communication during medical visits. American journal of public health. 2004 Dec;94(12):2084-90.

## Slide 5
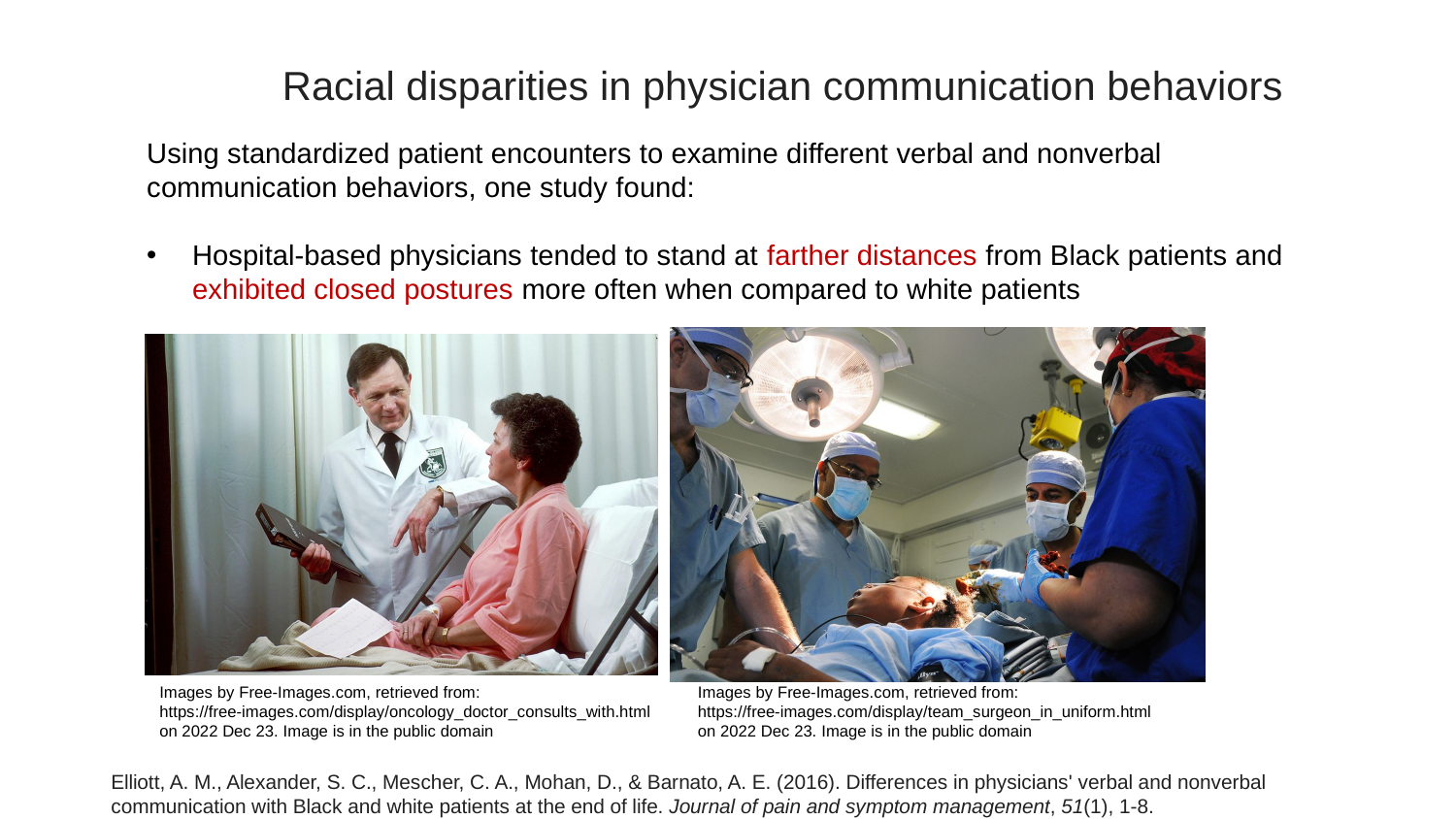

Racial disparities in physician communication behaviors
Using standardized patient encounters to examine different verbal and nonverbal communication behaviors, one study found:
Hospital-based physicians tended to stand at farther distances from Black patients and exhibited closed postures more often when compared to white patients
Images by Free-Images.com, retrieved from: https://free-images.com/display/oncology_doctor_consults_with.html
on 2022 Dec 23. Image is in the public domain
Images by Free-Images.com, retrieved from: https://free-images.com/display/team_surgeon_in_uniform.html
on 2022 Dec 23. Image is in the public domain
Elliott, A. M., Alexander, S. C., Mescher, C. A., Mohan, D., & Barnato, A. E. (2016). Differences in physicians' verbal and nonverbal communication with Black and white patients at the end of life. Journal of pain and symptom management, 51(1), 1-8.

## Slide 6
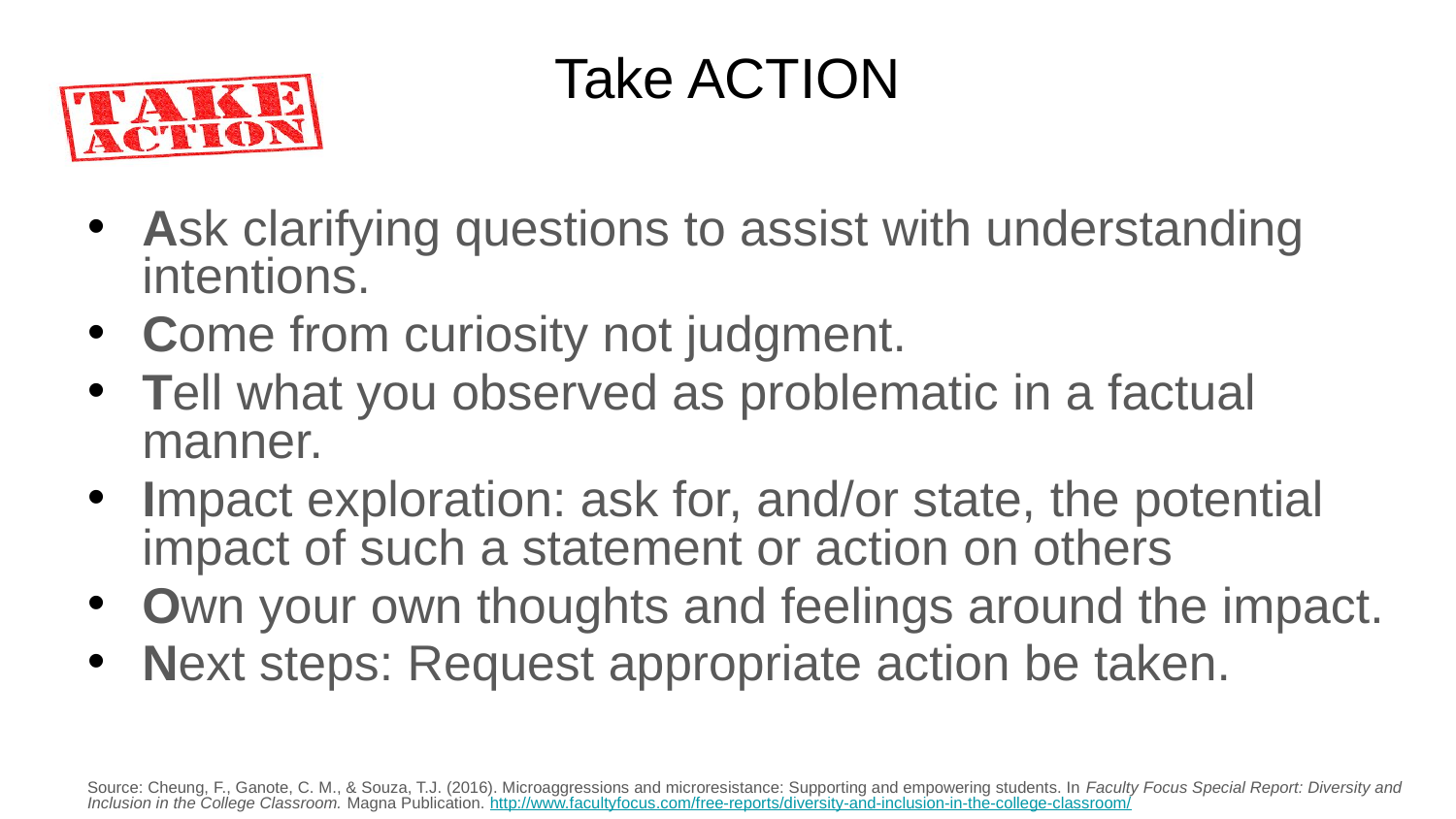

# Take ACTION
Ask clarifying questions to assist with understanding intentions.
Come from curiosity not judgment.
Tell what you observed as problematic in a factual manner.
Impact exploration: ask for, and/or state, the potential impact of such a statement or action on others
Own your own thoughts and feelings around the impact.
Next steps: Request appropriate action be taken.
Source: Cheung, F., Ganote, C. M., & Souza, T.J. (2016). Microaggressions and microresistance: Supporting and empowering students. In Faculty Focus Special Report: Diversity and Inclusion in the College Classroom. Magna Publication. http://www.facultyfocus.com/free-reports/diversity-and-inclusion-in-the-college-classroom/

## Slide 7
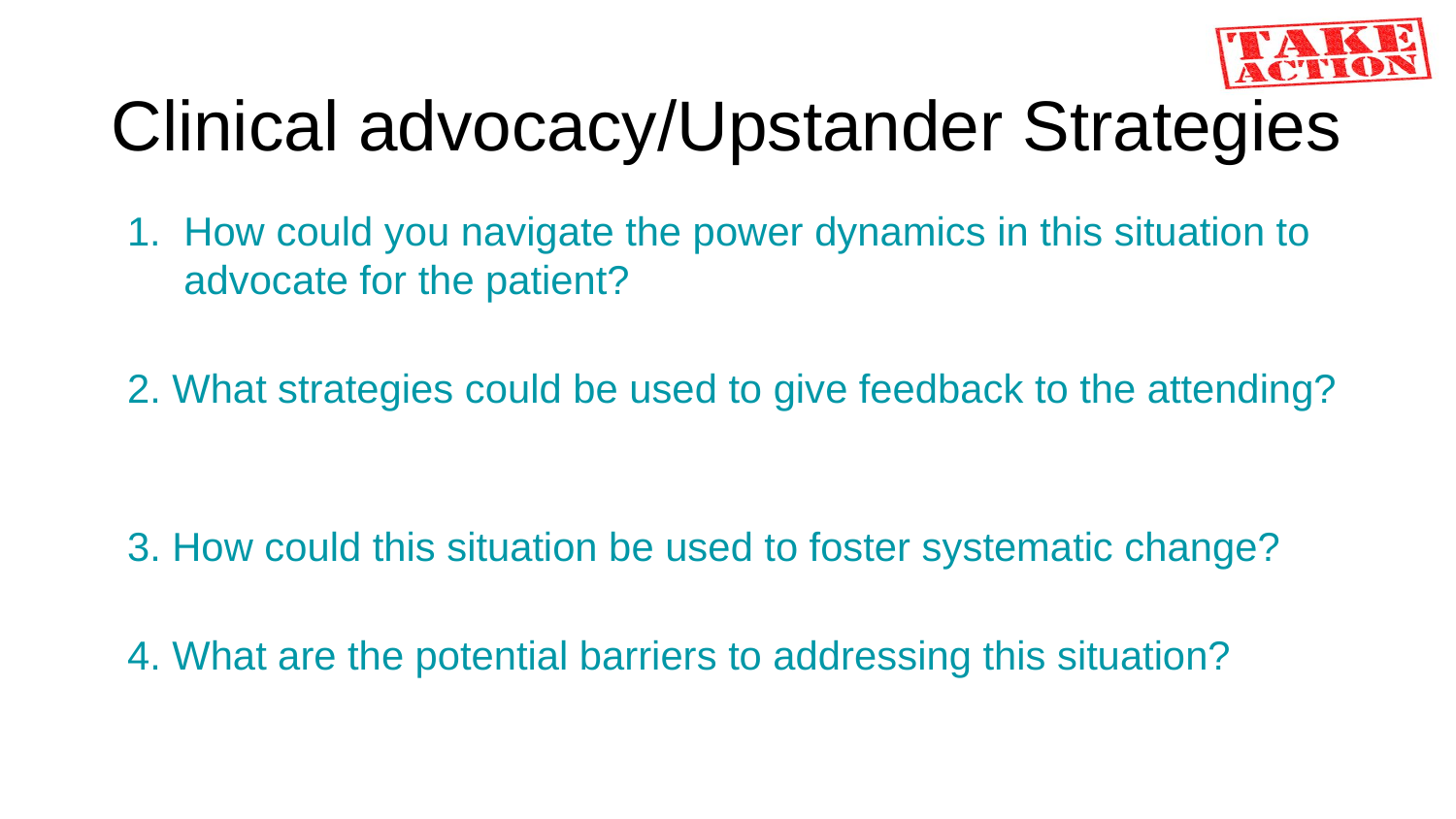

# Clinical advocacy/Upstander Strategies
How could you navigate the power dynamics in this situation to advocate for the patient?
2. What strategies could be used to give feedback to the attending?
3. How could this situation be used to foster systematic change?
4. What are the potential barriers to addressing this situation?

## Slide 8
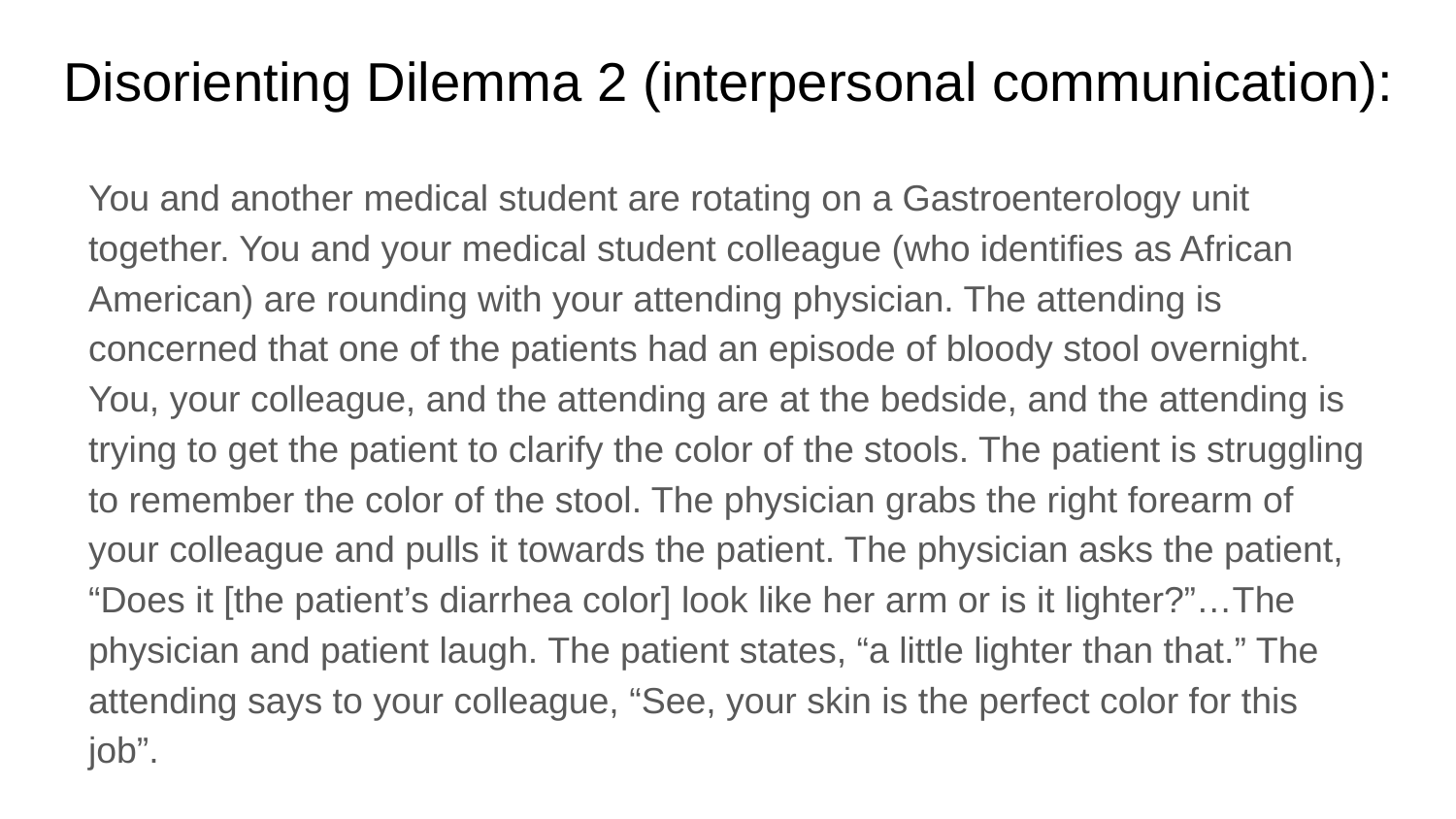

# Disorienting Dilemma 2 (interpersonal communication):
You and another medical student are rotating on a Gastroenterology unit together. You and your medical student colleague (who identifies as African American) are rounding with your attending physician. The attending is concerned that one of the patients had an episode of bloody stool overnight. You, your colleague, and the attending are at the bedside, and the attending is trying to get the patient to clarify the color of the stools. The patient is struggling to remember the color of the stool. The physician grabs the right forearm of your colleague and pulls it towards the patient. The physician asks the patient, “Does it [the patient’s diarrhea color] look like her arm or is it lighter?”…The physician and patient laugh. The patient states, “a little lighter than that.” The attending says to your colleague, “See, your skin is the perfect color for this job”.

## Slide 9
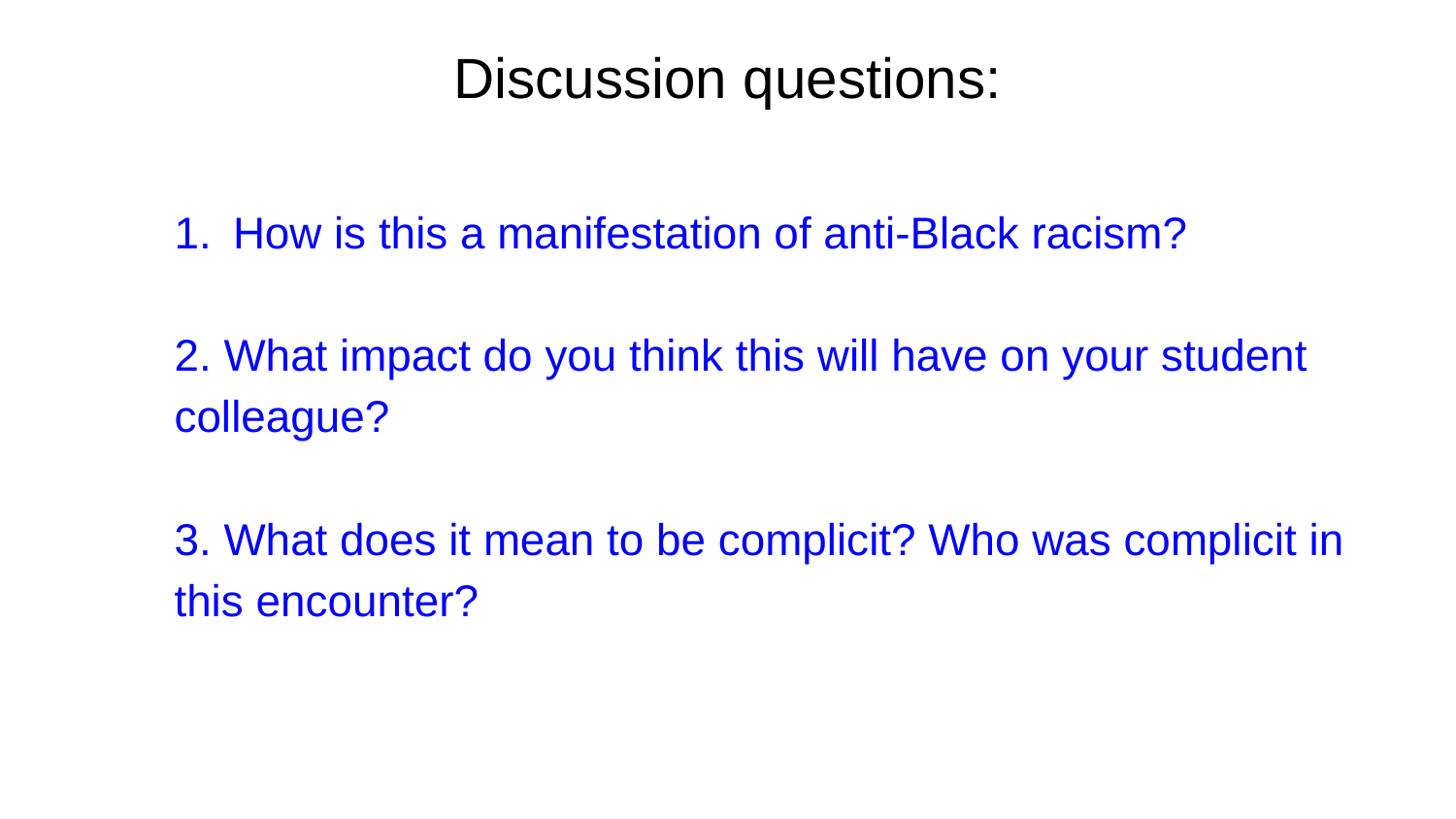

# Discussion questions:
How is this a manifestation of anti-Black racism?
2. What impact do you think this will have on your student colleague?
3. What does it mean to be complicit? Who was complicit in this encounter?

## Slide 10
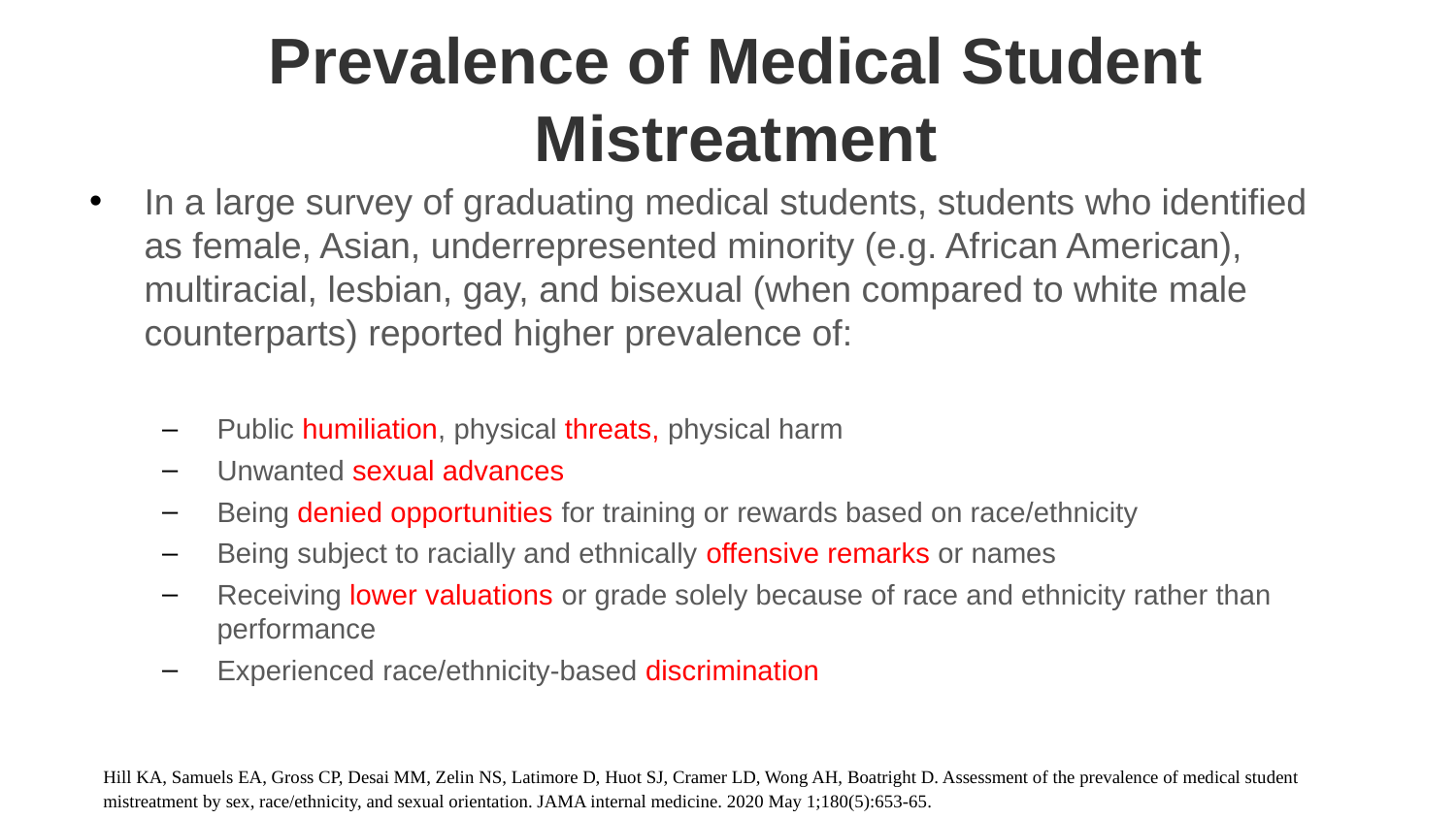

# Prevalence of Medical Student Mistreatment
In a large survey of graduating medical students, students who identified as female, Asian, underrepresented minority (e.g. African American), multiracial, lesbian, gay, and bisexual (when compared to white male counterparts) reported higher prevalence of:
Public humiliation, physical threats, physical harm
Unwanted sexual advances
Being denied opportunities for training or rewards based on race/ethnicity
Being subject to racially and ethnically offensive remarks or names
Receiving lower valuations or grade solely because of race and ethnicity rather than performance
Experienced race/ethnicity-based discrimination
Hill KA, Samuels EA, Gross CP, Desai MM, Zelin NS, Latimore D, Huot SJ, Cramer LD, Wong AH, Boatright D. Assessment of the prevalence of medical student mistreatment by sex, race/ethnicity, and sexual orientation. JAMA internal medicine. 2020 May 1;180(5):653-65.

## Slide 11
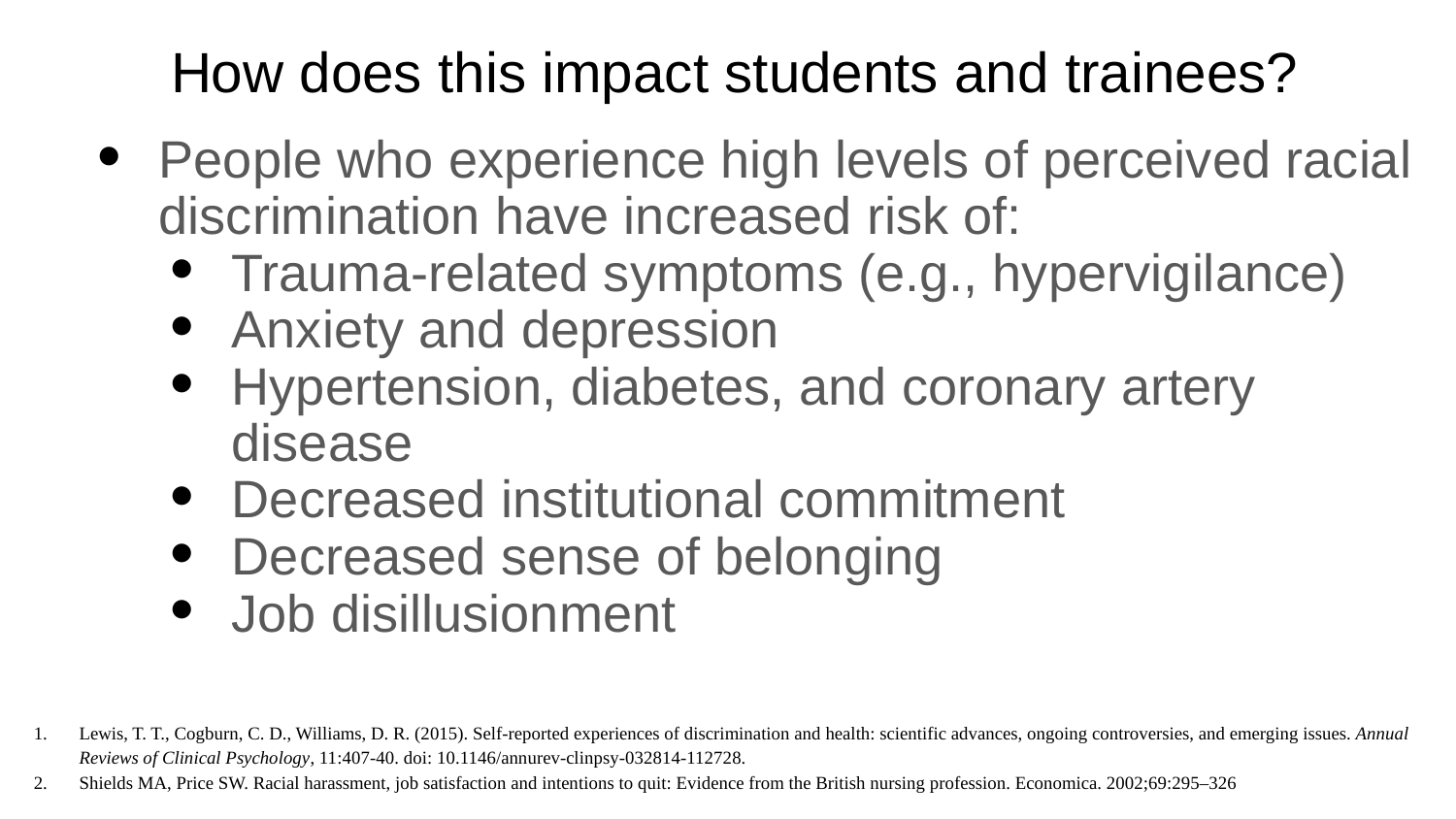

# How does this impact students and trainees?
People who experience high levels of perceived racial discrimination have increased risk of:
Trauma-related symptoms (e.g., hypervigilance)
Anxiety and depression
Hypertension, diabetes, and coronary artery disease
Decreased institutional commitment
Decreased sense of belonging
Job disillusionment
Lewis, T. T., Cogburn, C. D., Williams, D. R. (2015). Self-reported experiences of discrimination and health: scientific advances, ongoing controversies, and emerging issues. Annual Reviews of Clinical Psychology, 11:407-40. doi: 10.1146/annurev-clinpsy-032814-112728.
Shields MA, Price SW. Racial harassment, job satisfaction and intentions to quit: Evidence from the British nursing profession. Economica. 2002;69:295–326

## Slide 12
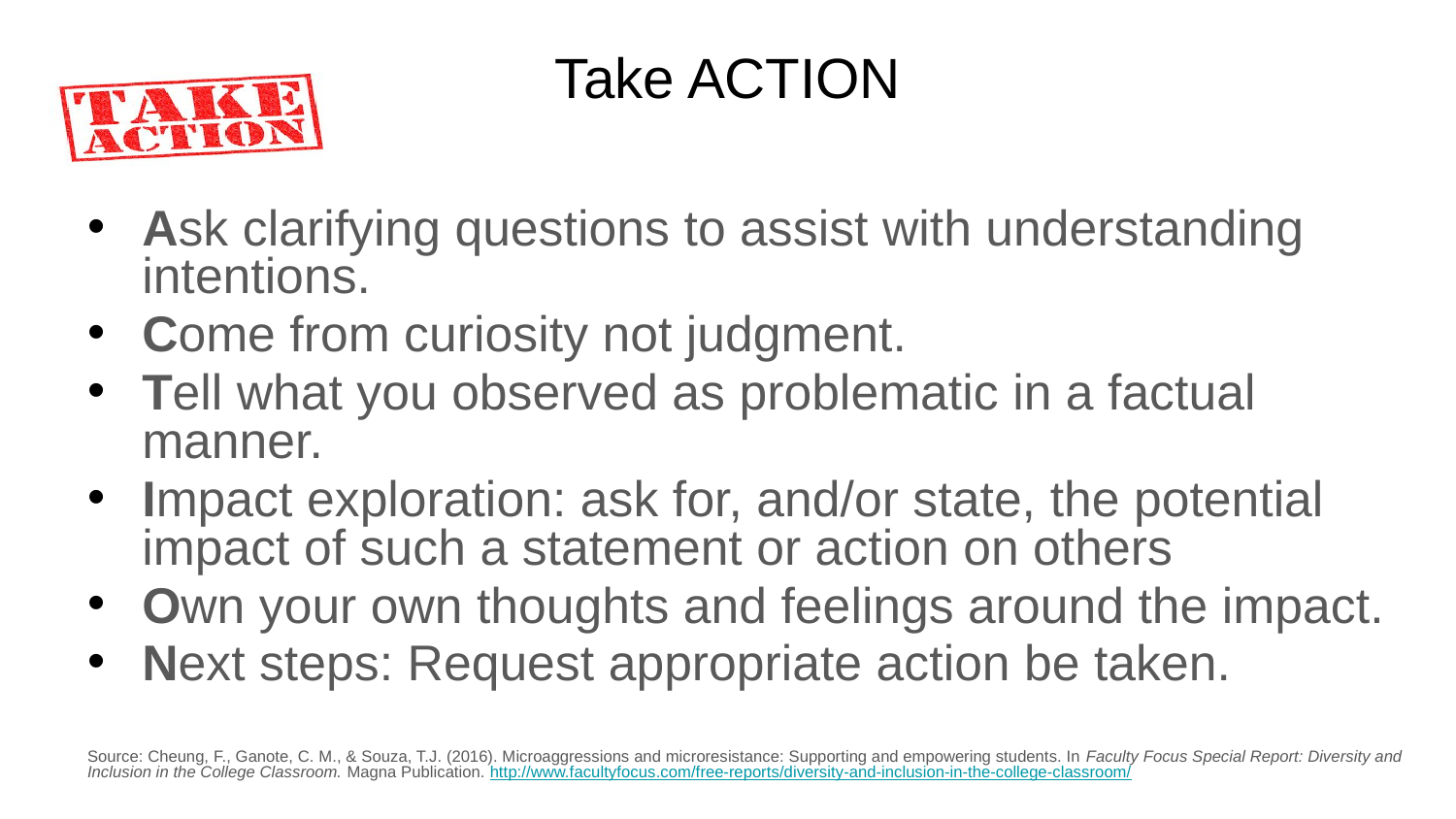

# Take ACTION
Ask clarifying questions to assist with understanding intentions.
Come from curiosity not judgment.
Tell what you observed as problematic in a factual manner.
Impact exploration: ask for, and/or state, the potential impact of such a statement or action on others
Own your own thoughts and feelings around the impact.
Next steps: Request appropriate action be taken.
Source: Cheung, F., Ganote, C. M., & Souza, T.J. (2016). Microaggressions and microresistance: Supporting and empowering students. In Faculty Focus Special Report: Diversity and Inclusion in the College Classroom. Magna Publication. http://www.facultyfocus.com/free-reports/diversity-and-inclusion-in-the-college-classroom/

## Slide 13
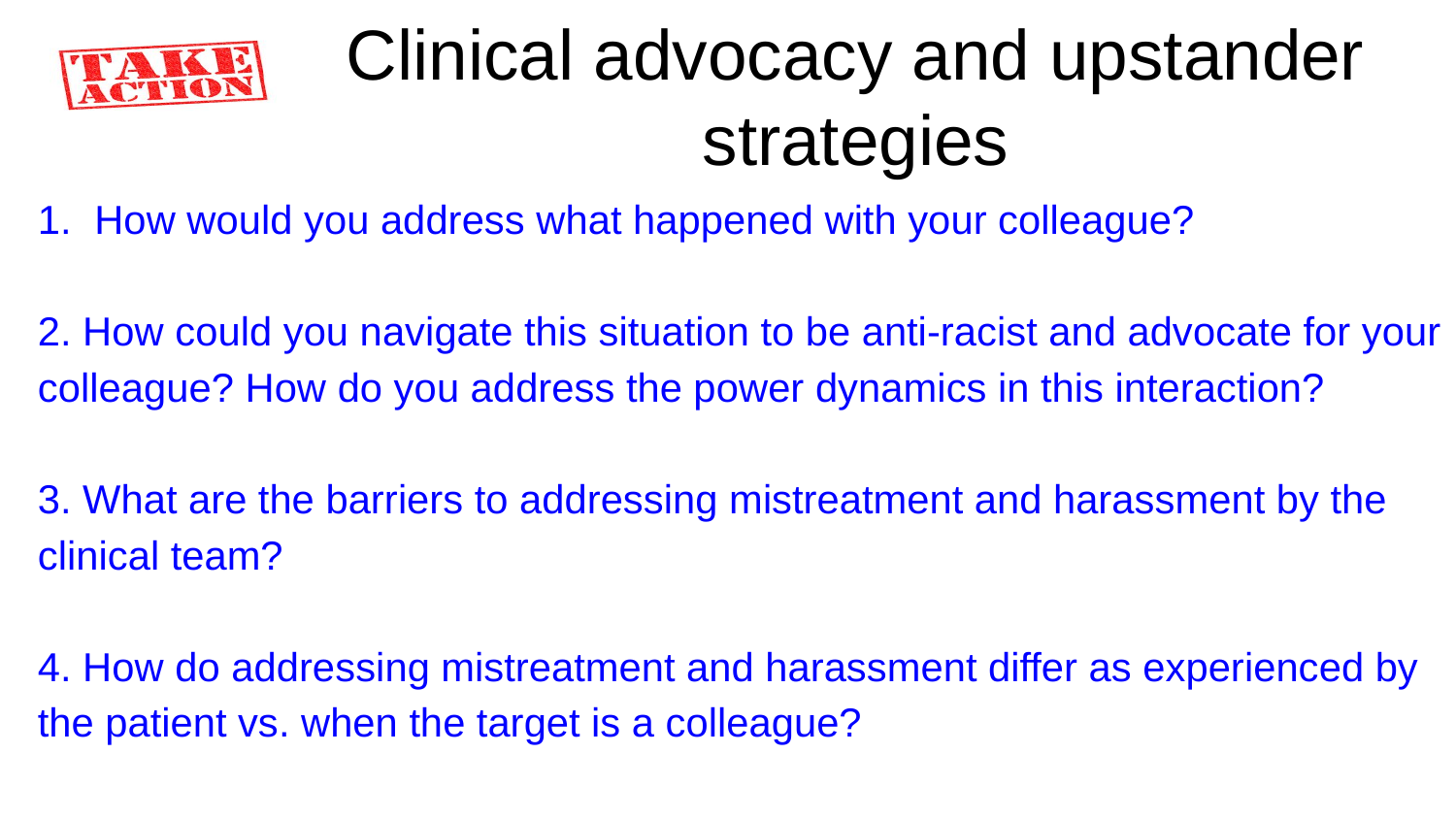

# Clinical advocacy and upstander strategies
How would you address what happened with your colleague?
2. How could you navigate this situation to be anti-racist and advocate for your colleague? How do you address the power dynamics in this interaction?
3. What are the barriers to addressing mistreatment and harassment by the clinical team?
4. How do addressing mistreatment and harassment differ as experienced by the patient vs. when the target is a colleague?

## Slide 14
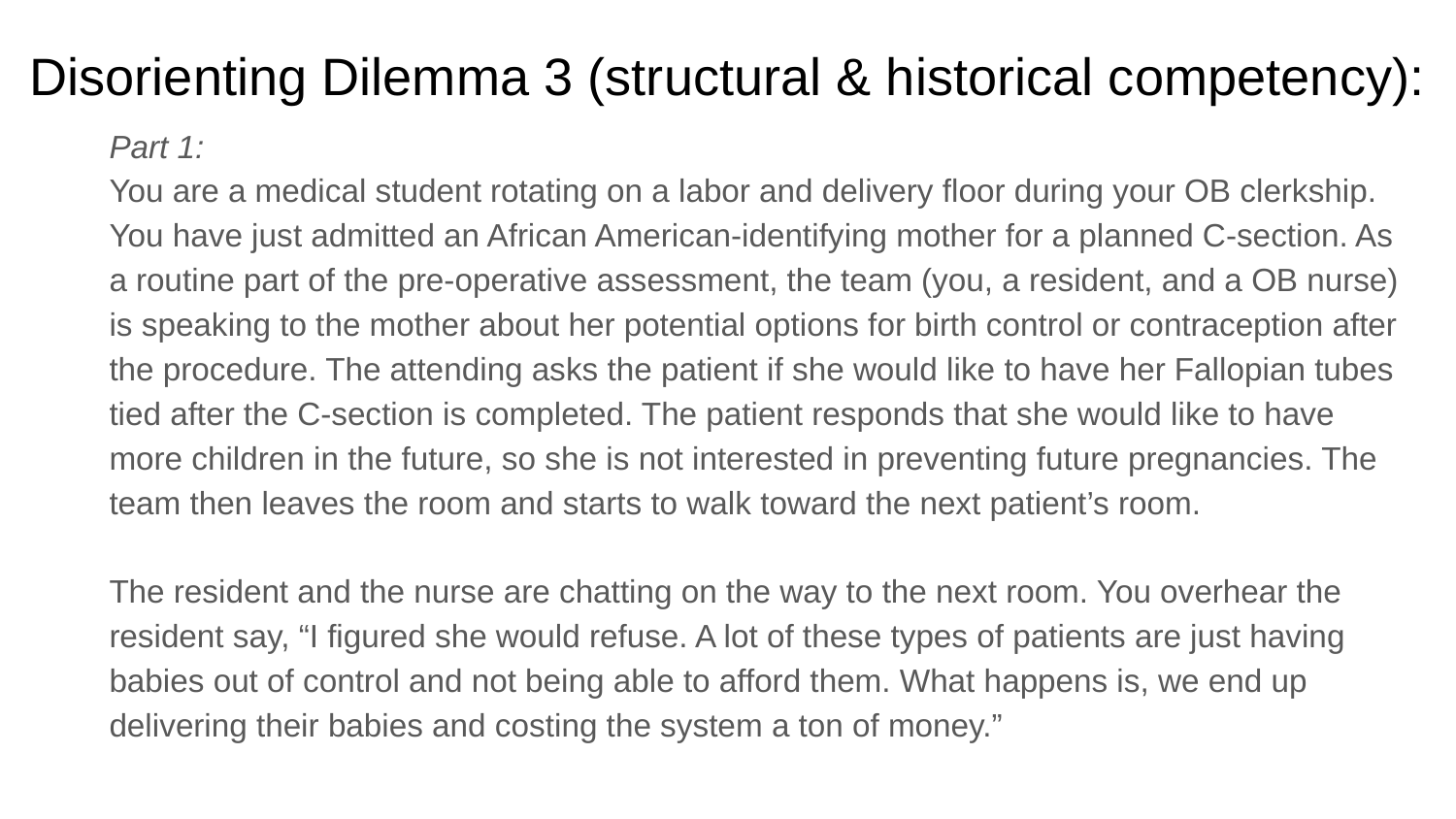

# Disorienting Dilemma 3 (structural & historical competency):
Part 1:
You are a medical student rotating on a labor and delivery floor during your OB clerkship. You have just admitted an African American-identifying mother for a planned C-section. As a routine part of the pre-operative assessment, the team (you, a resident, and a OB nurse) is speaking to the mother about her potential options for birth control or contraception after the procedure. The attending asks the patient if she would like to have her Fallopian tubes tied after the C-section is completed. The patient responds that she would like to have more children in the future, so she is not interested in preventing future pregnancies. The team then leaves the room and starts to walk toward the next patient’s room.
The resident and the nurse are chatting on the way to the next room. You overhear the resident say, “I figured she would refuse. A lot of these types of patients are just having babies out of control and not being able to afford them. What happens is, we end up delivering their babies and costing the system a ton of money.”

## Slide 15
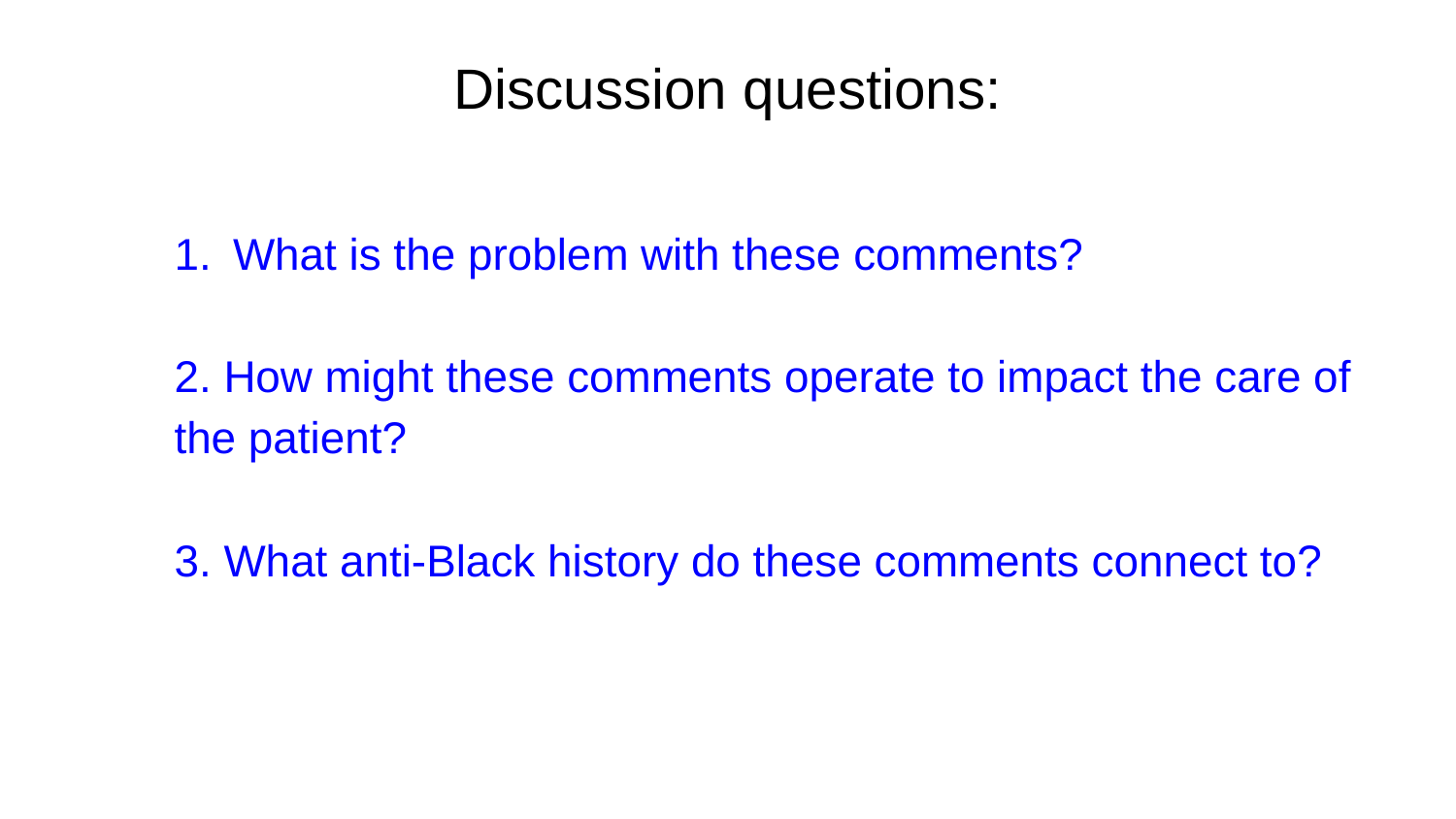

# Discussion questions:
What is the problem with these comments?
2. How might these comments operate to impact the care of the patient?
3. What anti-Black history do these comments connect to?

## Slide 16
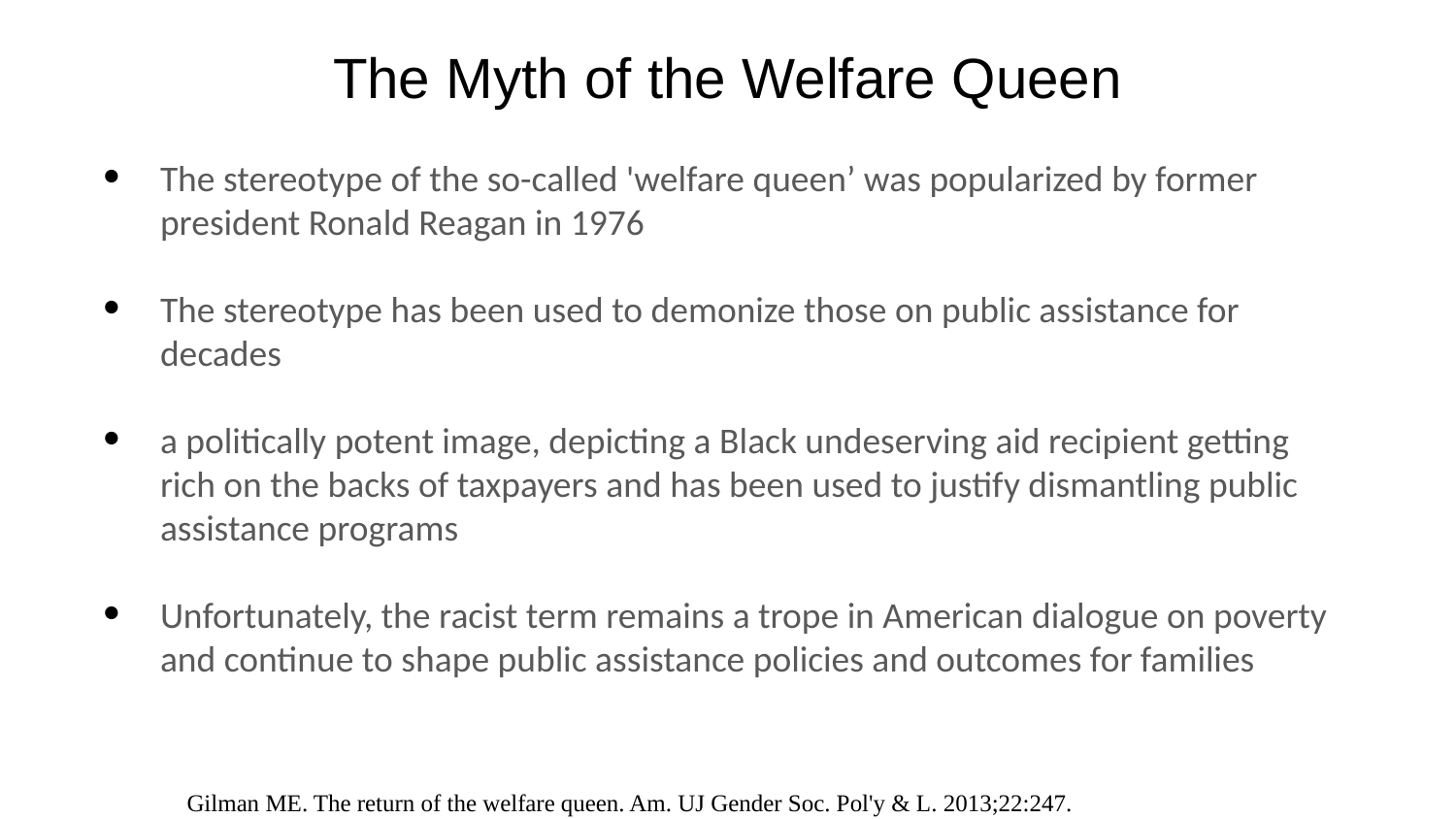

# The Myth of the Welfare Queen
The stereotype of the so-called 'welfare queen’ was popularized by former president Ronald Reagan in 1976
The stereotype has been used to demonize those on public assistance for decades
a politically potent image, depicting a Black undeserving aid recipient getting rich on the backs of taxpayers and has been used to justify dismantling public assistance programs
Unfortunately, the racist term remains a trope in American dialogue on poverty and continue to shape public assistance policies and outcomes for families
Gilman ME. The return of the welfare queen. Am. UJ Gender Soc. Pol'y & L. 2013;22:247.

## Slide 17
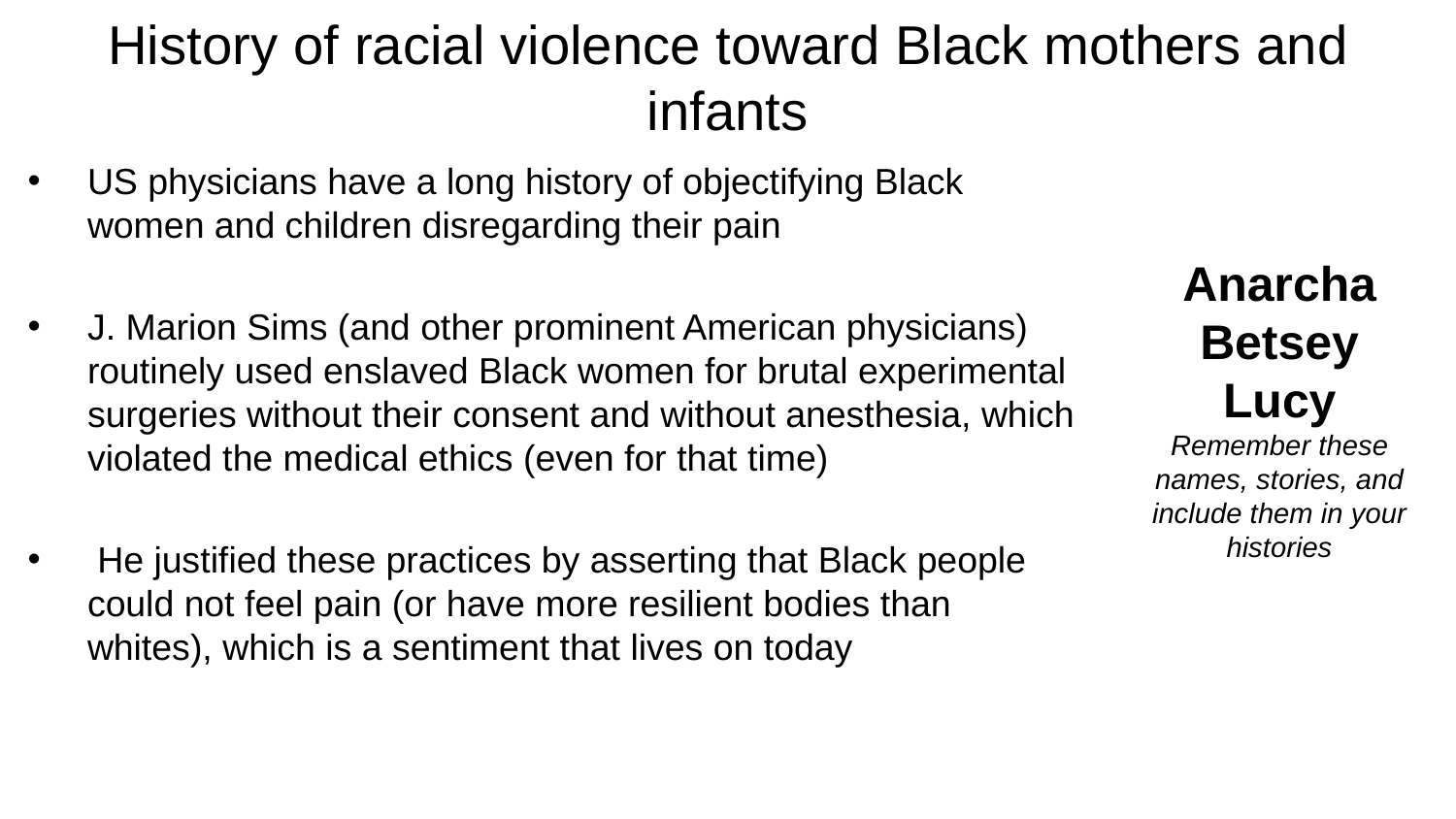

# History of racial violence toward Black mothers and infants
US physicians have a long history of objectifying Black women and children disregarding their pain
J. Marion Sims (and other prominent American physicians) routinely used enslaved Black women for brutal experimental surgeries without their consent and without anesthesia, which violated the medical ethics (even for that time)
 He justified these practices by asserting that Black people could not feel pain (or have more resilient bodies than whites), which is a sentiment that lives on today
Anarcha
Betsey
Lucy
Remember these names, stories, and include them in your histories

## Slide 18
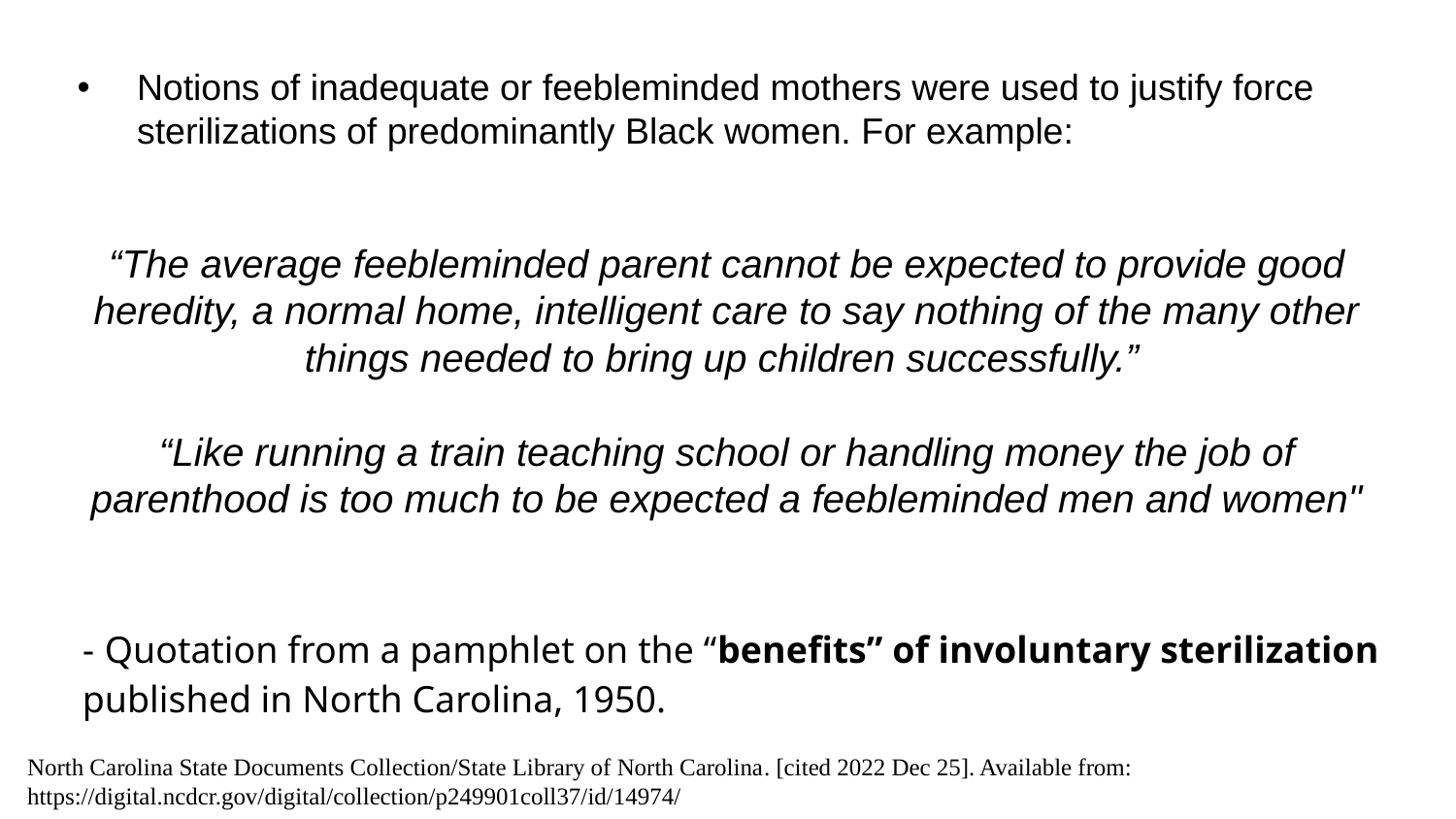

Notions of inadequate or feebleminded mothers were used to justify force sterilizations of predominantly Black women. For example:
# “The average feebleminded parent cannot be expected to provide good heredity, a normal home, intelligent care to say nothing of the many other things needed to bring up children successfully.” “Like running a train teaching school or handling money the job of parenthood is too much to be expected a feebleminded men and women"
- Quotation from a pamphlet on the “benefits” of involuntary sterilization published in North Carolina, 1950.
North Carolina State Documents Collection/State Library of North Carolina. [cited 2022 Dec 25]. Available from: https://digital.ncdcr.gov/digital/collection/p249901coll37/id/14974/

## Slide 19
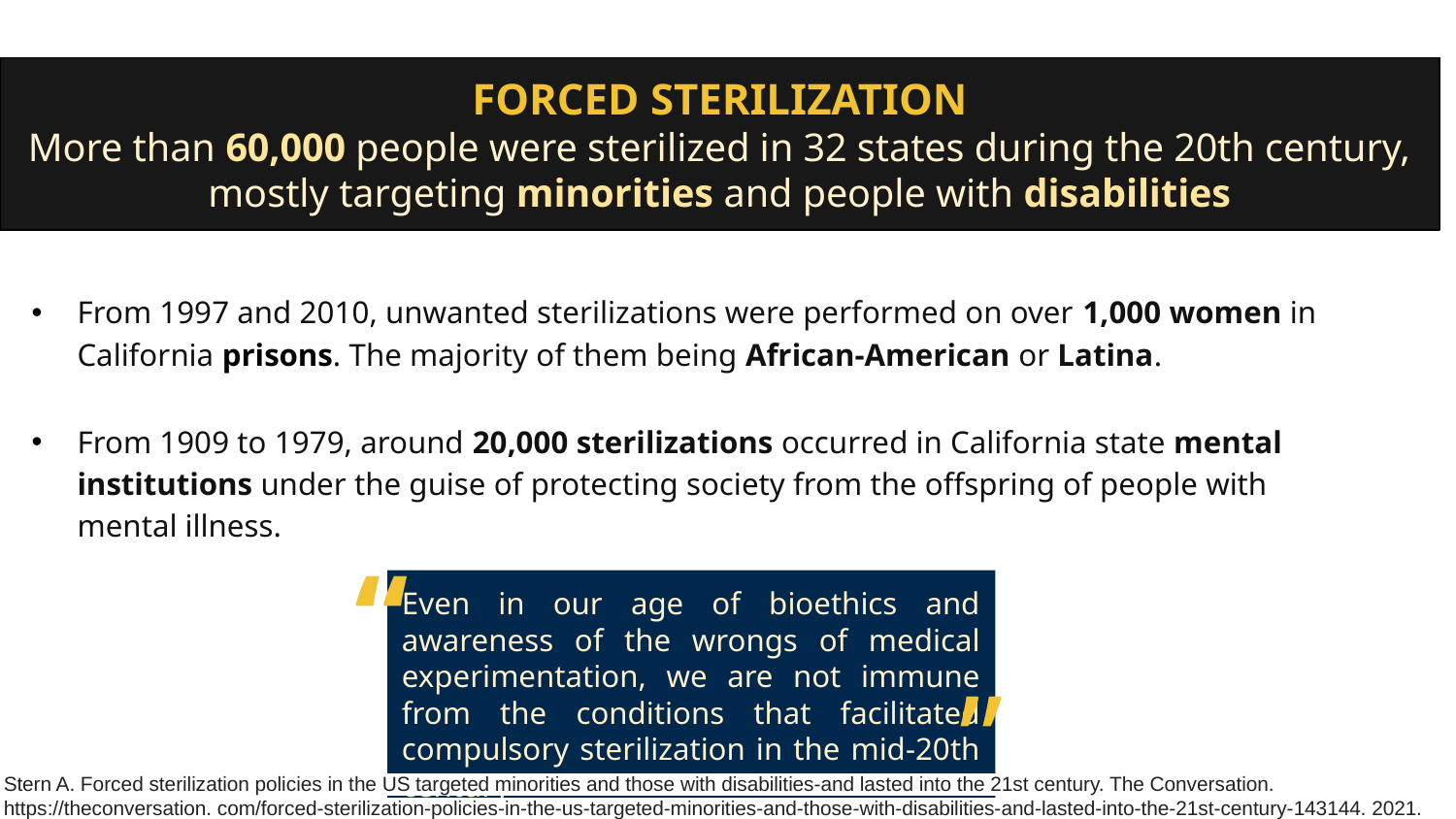

FORCED STERILIZATION
More than 60,000 people were sterilized in 32 states during the 20th century, mostly targeting minorities and people with disabilities
From 1997 and 2010, unwanted sterilizations were performed on over 1,000 women in California prisons. The majority of them being African-American or Latina.
From 1909 to 1979, around 20,000 sterilizations occurred in California state mental institutions under the guise of protecting society from the offspring of people with mental illness.
“
Even in our age of bioethics and awareness of the wrongs of medical experimentation, we are not immune from the conditions that facilitated compulsory sterilization in the mid-20th century
”
Stern A. Forced sterilization policies in the US targeted minorities and those with disabilities-and lasted into the 21st century. The Conversation. https://theconversation. com/forced-sterilization-policies-in-the-us-targeted-minorities-and-those-with-disabilities-and-lasted-into-the-21st-century-143144. 2021.

## Slide 20
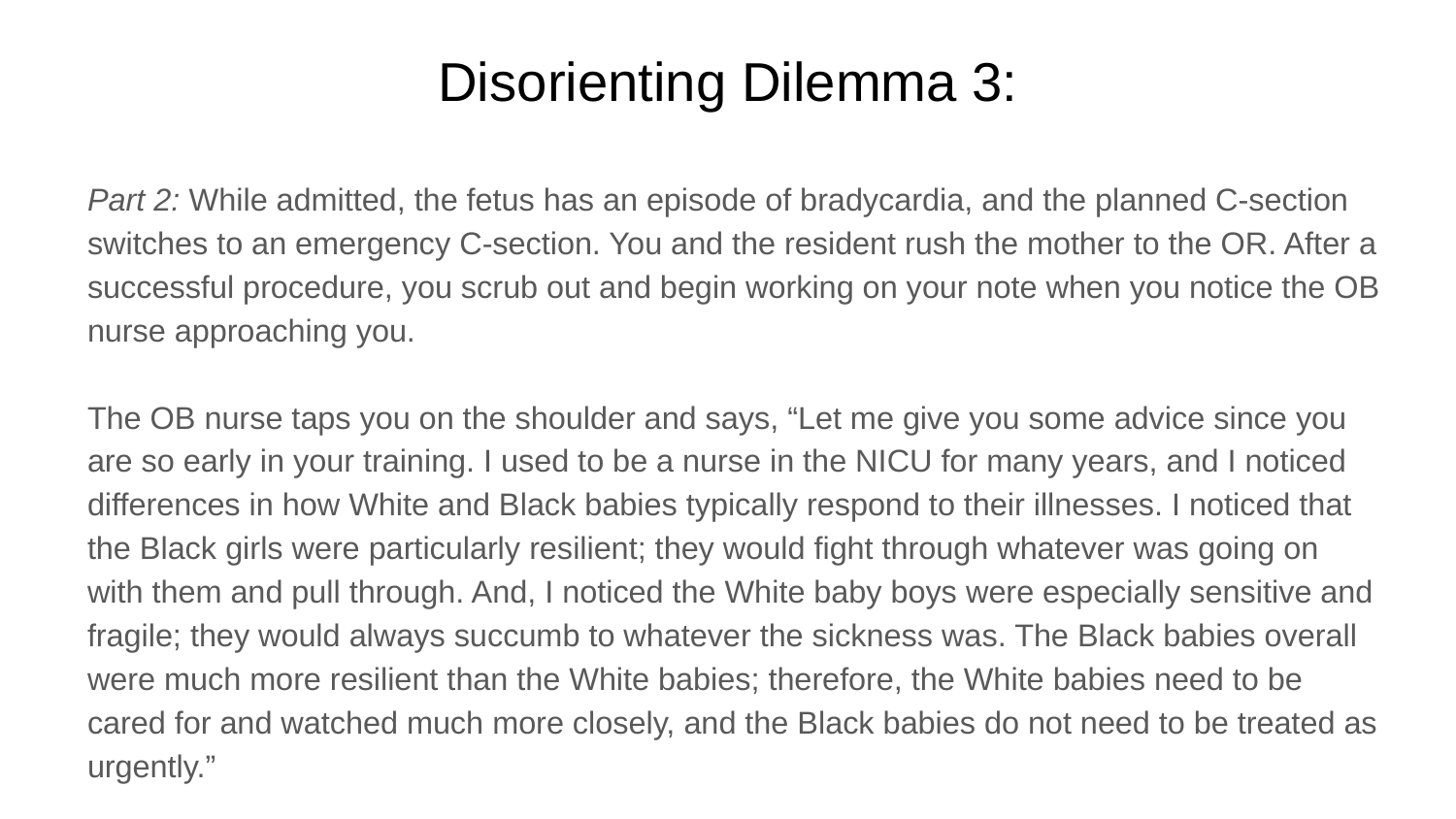

# Disorienting Dilemma 3:
Part 2: While admitted, the fetus has an episode of bradycardia, and the planned C-section switches to an emergency C-section. You and the resident rush the mother to the OR. After a successful procedure, you scrub out and begin working on your note when you notice the OB nurse approaching you.
The OB nurse taps you on the shoulder and says, “Let me give you some advice since you are so early in your training. I used to be a nurse in the NICU for many years, and I noticed differences in how White and Black babies typically respond to their illnesses. I noticed that the Black girls were particularly resilient; they would fight through whatever was going on with them and pull through. And, I noticed the White baby boys were especially sensitive and fragile; they would always succumb to whatever the sickness was. The Black babies overall were much more resilient than the White babies; therefore, the White babies need to be cared for and watched much more closely, and the Black babies do not need to be treated as urgently.”

## Slide 21
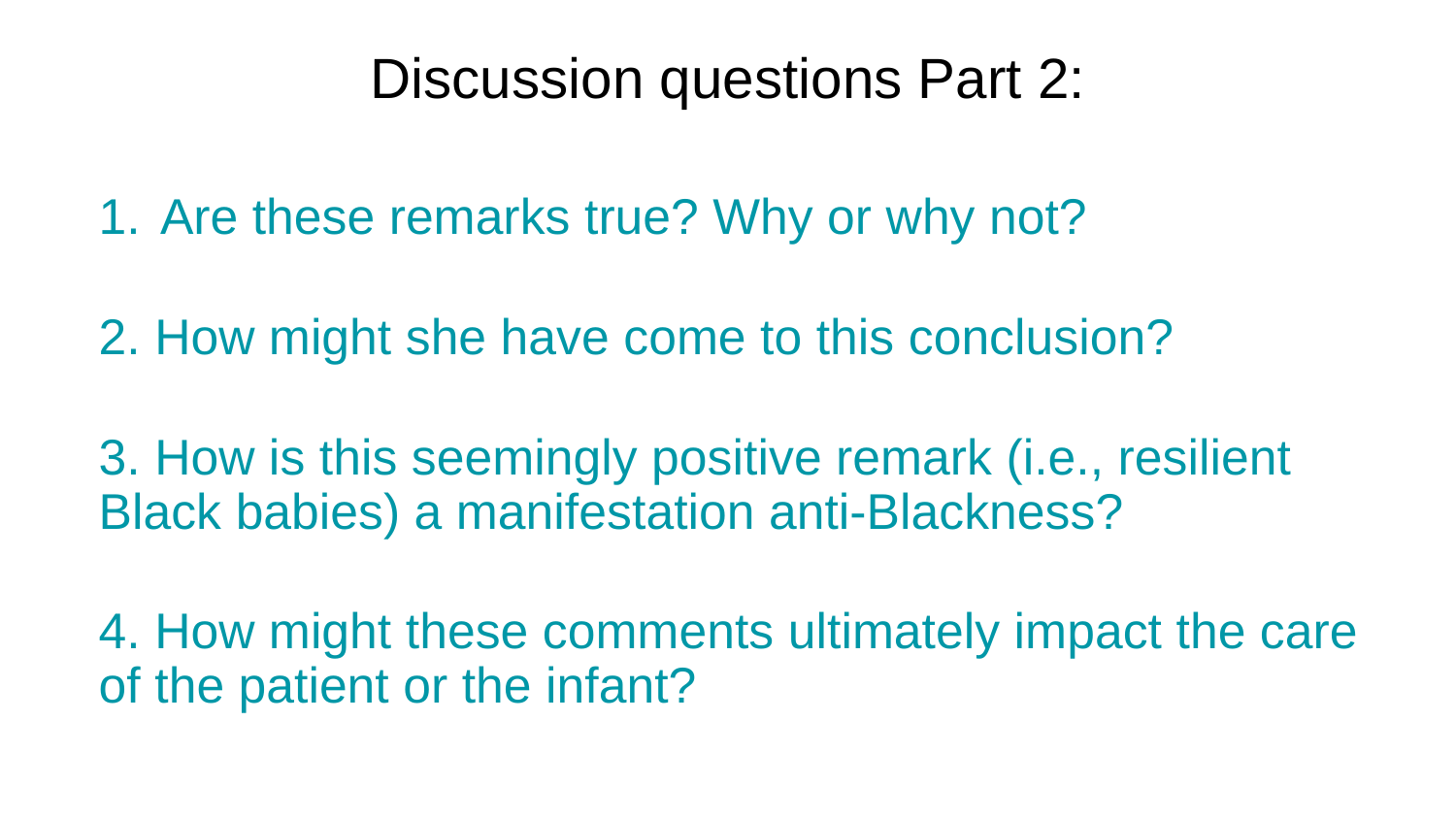

# Discussion questions Part 2:
Are these remarks true? Why or why not?
2. How might she have come to this conclusion?
3. How is this seemingly positive remark (i.e., resilient Black babies) a manifestation anti-Blackness?
4. How might these comments ultimately impact the care of the patient or the infant?

## Slide 22
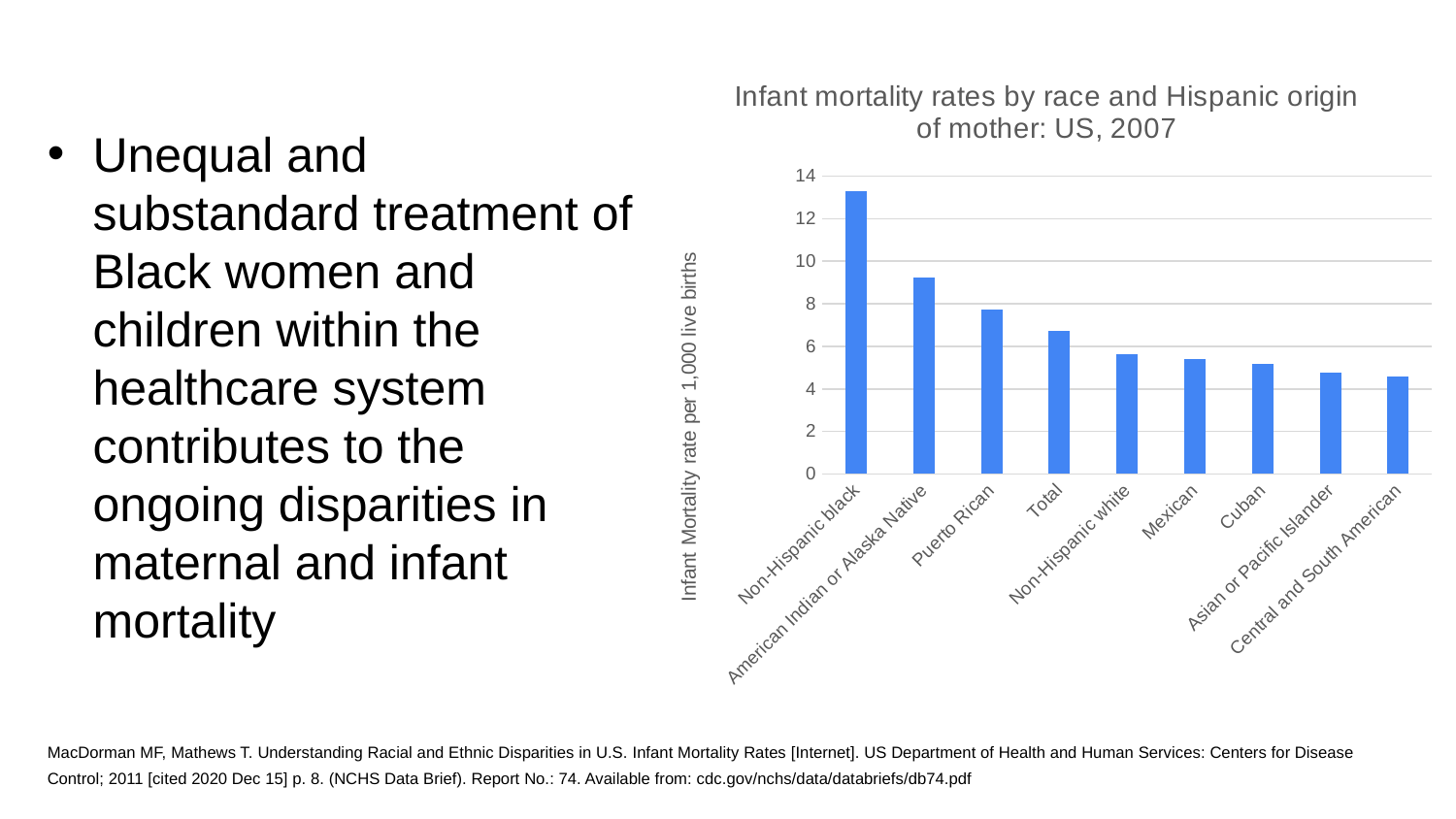

### Chart: Infant mortality rates by race and Hispanic origin of mother: US, 2007
| Category | |
|---|---|
| Non-Hispanic black | 13.31 |
| American Indian or Alaska Native | 9.22 |
| Puerto Rican | 7.71 |
| Total | 6.75 |
| Non-Hispanic white | 5.63 |
| Mexican | 5.42 |
| Cuban | 5.18 |
| Asian or Pacific Islander | 4.78 |
| Central and South American | 4.57 |Unequal and substandard treatment of Black women and children within the healthcare system contributes to the ongoing disparities in maternal and infant mortality
MacDorman MF, Mathews T. Understanding Racial and Ethnic Disparities in U.S. Infant Mortality Rates [Internet]. US Department of Health and Human Services: Centers for Disease Control; 2011 [cited 2020 Dec 15] p. 8. (NCHS Data Brief). Report No.: 74. Available from: cdc.gov/nchs/data/databriefs/db74.pdf

## Slide 23
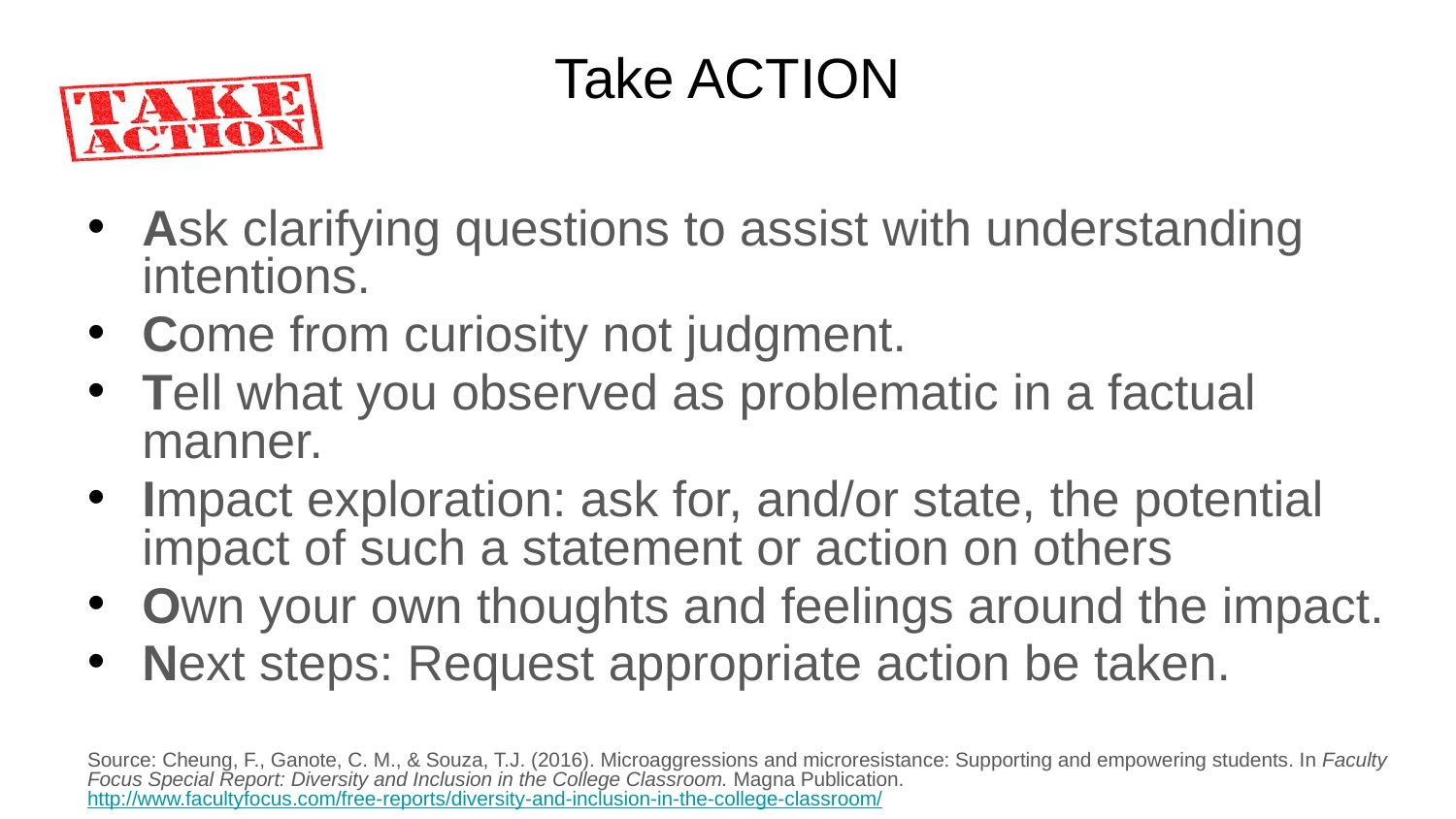

# Take ACTION
Ask clarifying questions to assist with understanding intentions.
Come from curiosity not judgment.
Tell what you observed as problematic in a factual manner.
Impact exploration: ask for, and/or state, the potential impact of such a statement or action on others
Own your own thoughts and feelings around the impact.
Next steps: Request appropriate action be taken.
Source: Cheung, F., Ganote, C. M., & Souza, T.J. (2016). Microaggressions and microresistance: Supporting and empowering students. In Faculty Focus Special Report: Diversity and Inclusion in the College Classroom. Magna Publication. http://www.facultyfocus.com/free-reports/diversity-and-inclusion-in-the-college-classroom/

## Slide 24
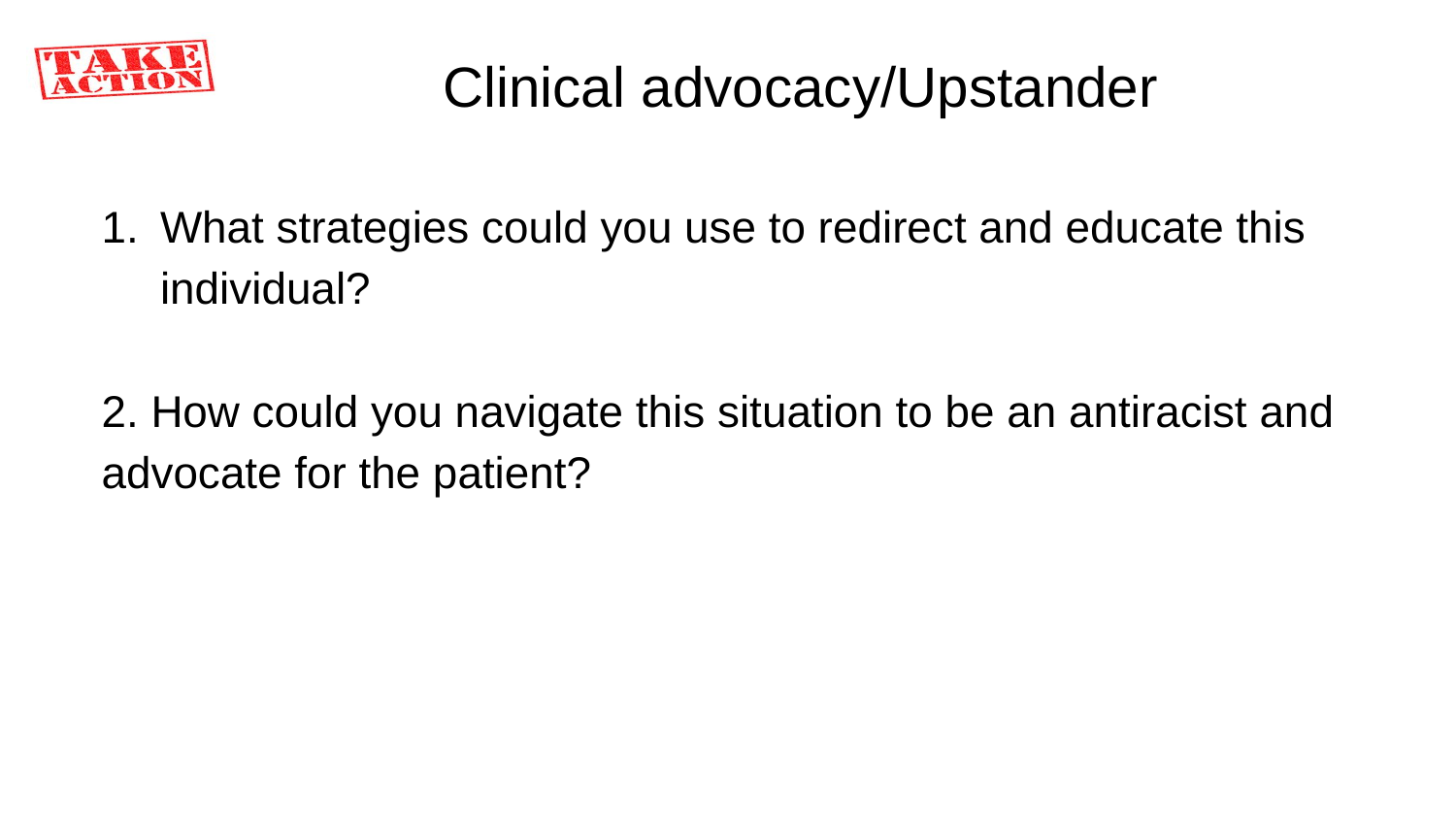

# Clinical advocacy/Upstander
What strategies could you use to redirect and educate this individual?
2. How could you navigate this situation to be an antiracist and advocate for the patient?

## Slide 25
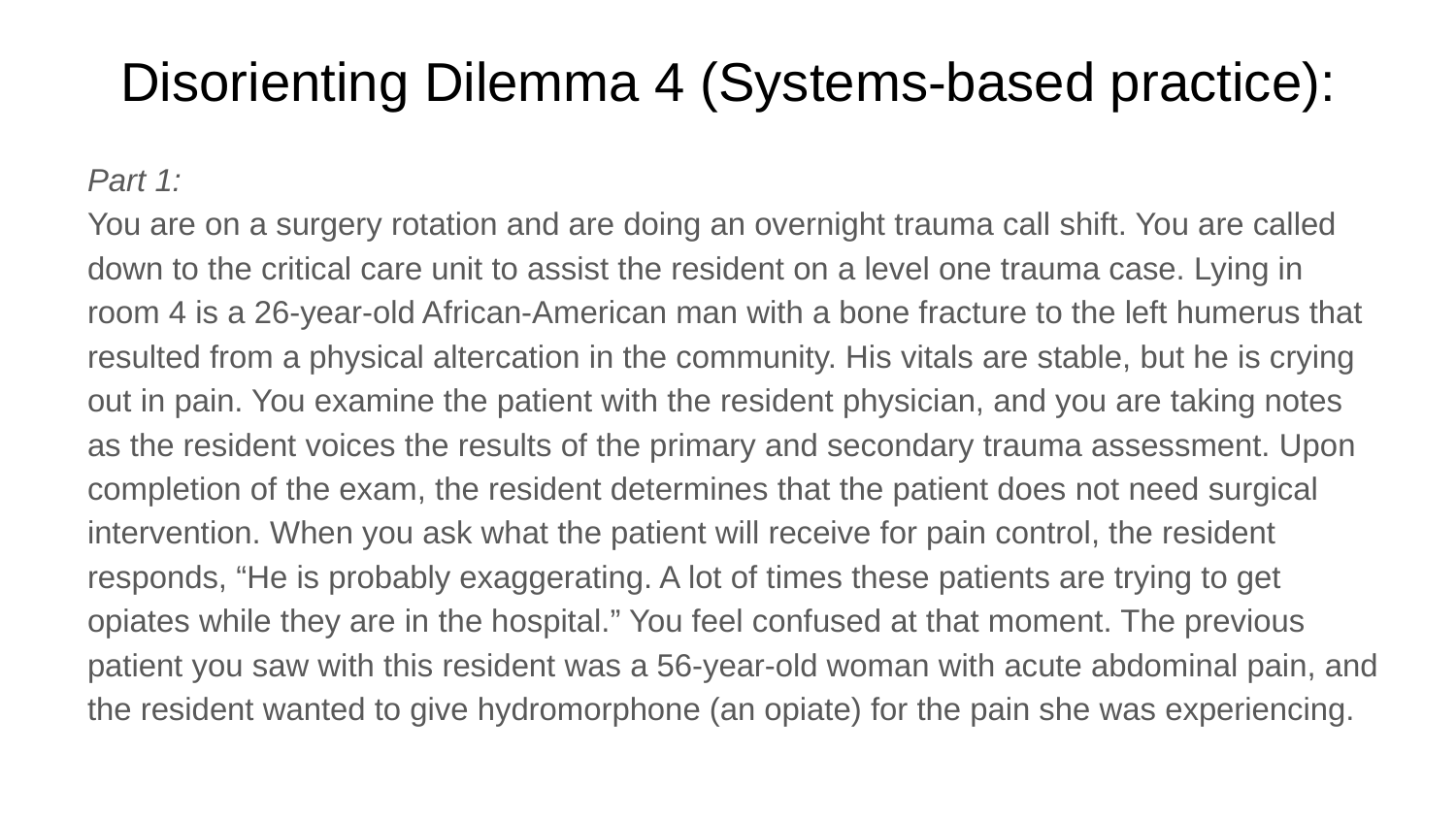

# Disorienting Dilemma 4 (Systems-based practice):
Part 1:
You are on a surgery rotation and are doing an overnight trauma call shift. You are called down to the critical care unit to assist the resident on a level one trauma case. Lying in room 4 is a 26-year-old African-American man with a bone fracture to the left humerus that resulted from a physical altercation in the community. His vitals are stable, but he is crying out in pain. You examine the patient with the resident physician, and you are taking notes as the resident voices the results of the primary and secondary trauma assessment. Upon completion of the exam, the resident determines that the patient does not need surgical intervention. When you ask what the patient will receive for pain control, the resident responds, “He is probably exaggerating. A lot of times these patients are trying to get opiates while they are in the hospital.” You feel confused at that moment. The previous patient you saw with this resident was a 56-year-old woman with acute abdominal pain, and the resident wanted to give hydromorphone (an opiate) for the pain she was experiencing.

## Slide 26
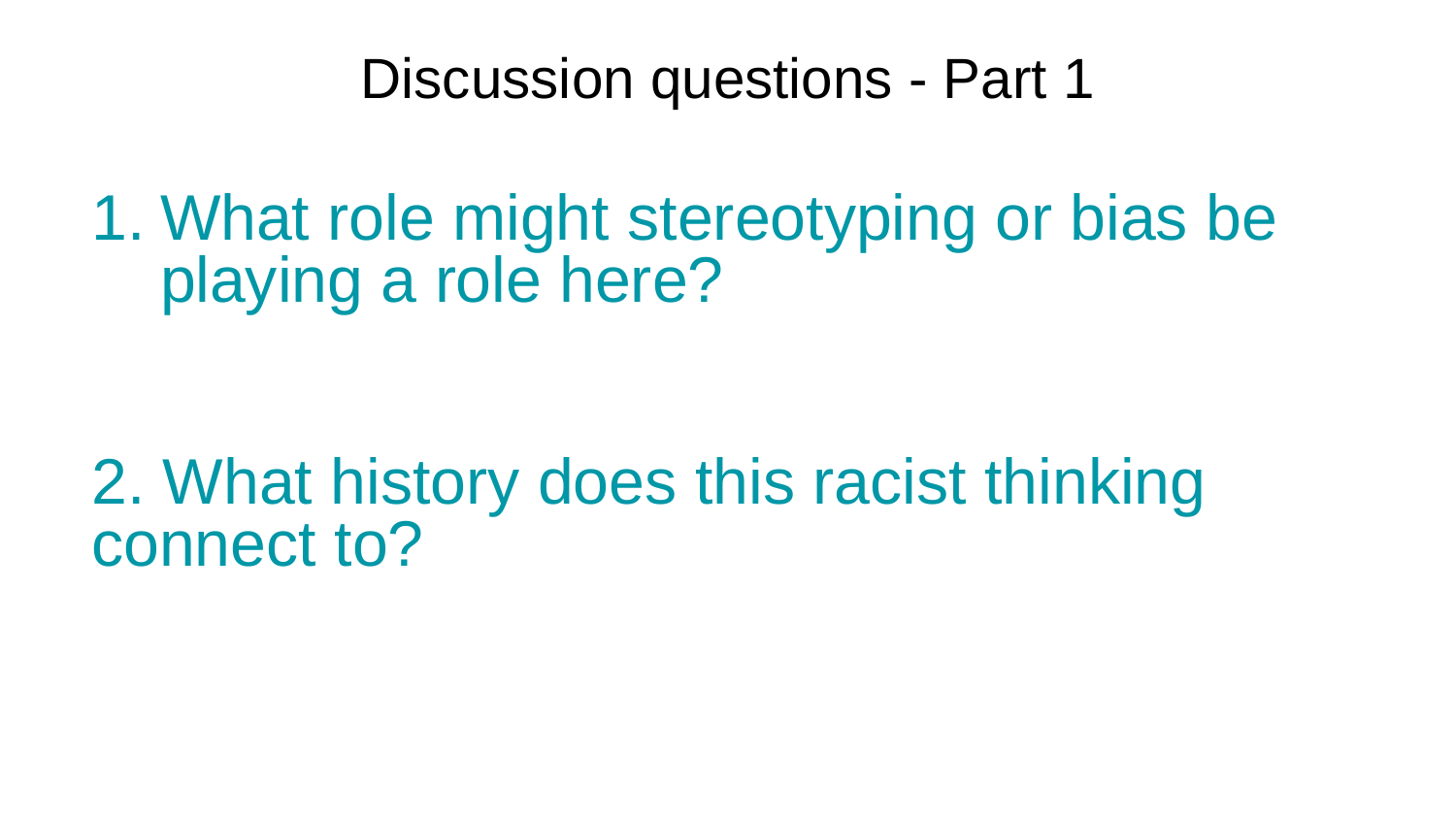

# Discussion questions - Part 1
What role might stereotyping or bias be playing a role here?
2. What history does this racist thinking connect to?

## Slide 27
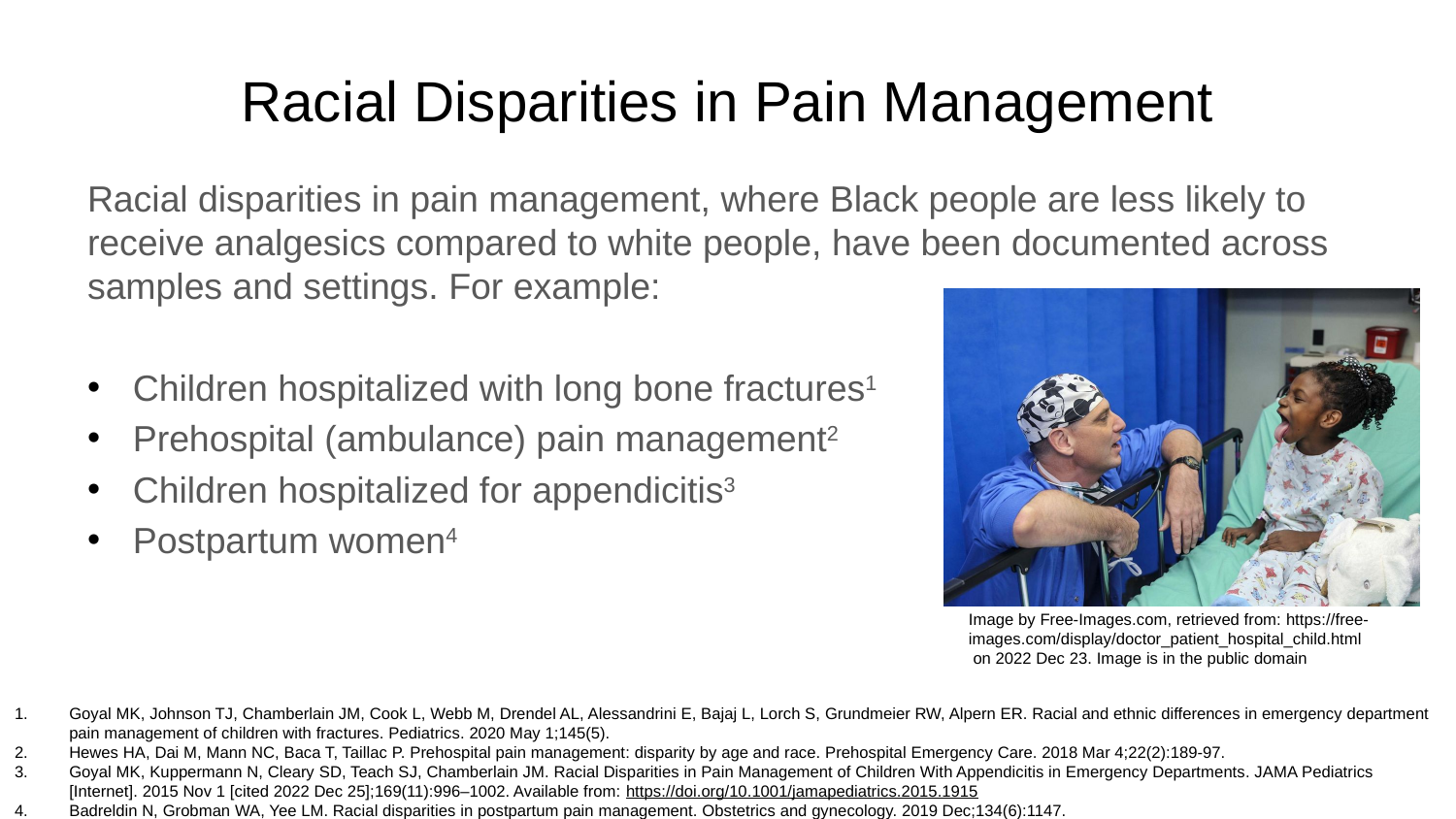

# Racial Disparities in Pain Management
Racial disparities in pain management, where Black people are less likely to receive analgesics compared to white people, have been documented across samples and settings. For example:
Children hospitalized with long bone fractures1
Prehospital (ambulance) pain management2
Children hospitalized for appendicitis3
Postpartum women4
Image by Free-Images.com, retrieved from: https://free-images.com/display/doctor_patient_hospital_child.html
 on 2022 Dec 23. Image is in the public domain
Goyal MK, Johnson TJ, Chamberlain JM, Cook L, Webb M, Drendel AL, Alessandrini E, Bajaj L, Lorch S, Grundmeier RW, Alpern ER. Racial and ethnic differences in emergency department pain management of children with fractures. Pediatrics. 2020 May 1;145(5).
Hewes HA, Dai M, Mann NC, Baca T, Taillac P. Prehospital pain management: disparity by age and race. Prehospital Emergency Care. 2018 Mar 4;22(2):189-97.
Goyal MK, Kuppermann N, Cleary SD, Teach SJ, Chamberlain JM. Racial Disparities in Pain Management of Children With Appendicitis in Emergency Departments. JAMA Pediatrics [Internet]. 2015 Nov 1 [cited 2022 Dec 25];169(11):996–1002. Available from: https://doi.org/10.1001/jamapediatrics.2015.1915
Badreldin N, Grobman WA, Yee LM. Racial disparities in postpartum pain management. Obstetrics and gynecology. 2019 Dec;134(6):1147.

## Slide 28
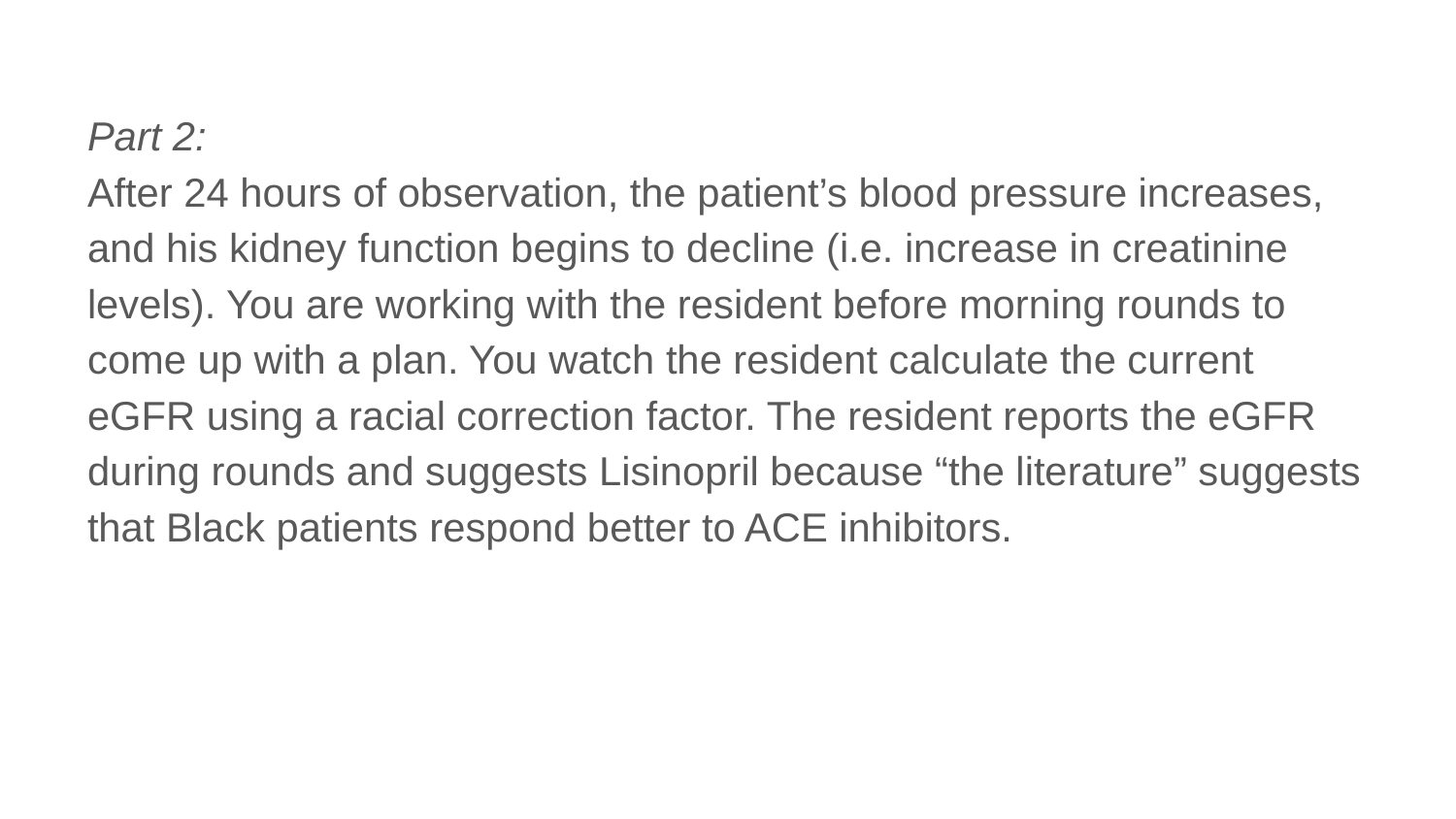

Part 2:
After 24 hours of observation, the patient’s blood pressure increases, and his kidney function begins to decline (i.e. increase in creatinine levels). You are working with the resident before morning rounds to come up with a plan. You watch the resident calculate the current eGFR using a racial correction factor. The resident reports the eGFR during rounds and suggests Lisinopril because “the literature” suggests that Black patients respond better to ACE inhibitors.

## Slide 29
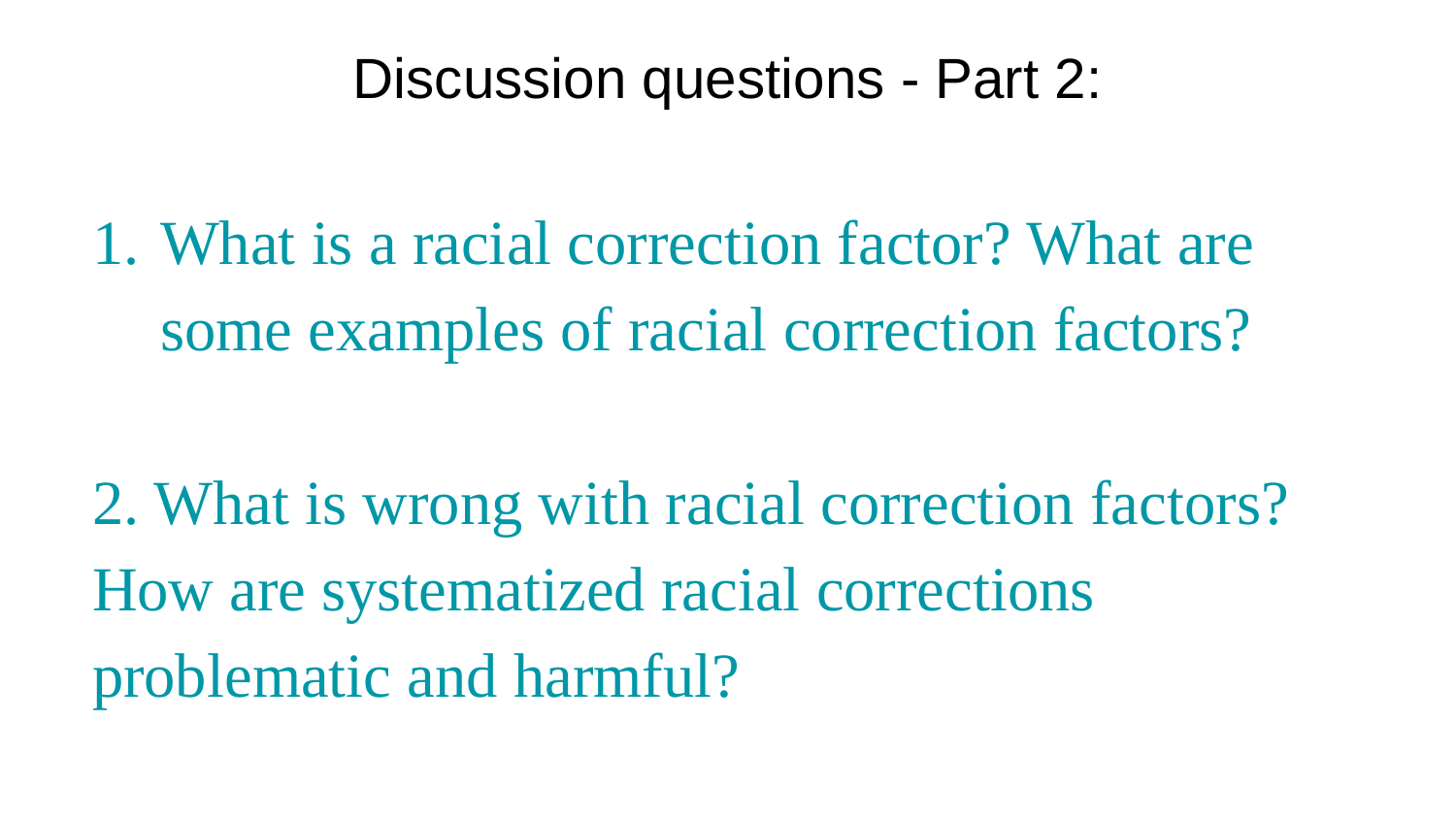

# Discussion questions - Part 2:
What is a racial correction factor? What are some examples of racial correction factors?
2. What is wrong with racial correction factors? How are systematized racial corrections problematic and harmful?

## Slide 30
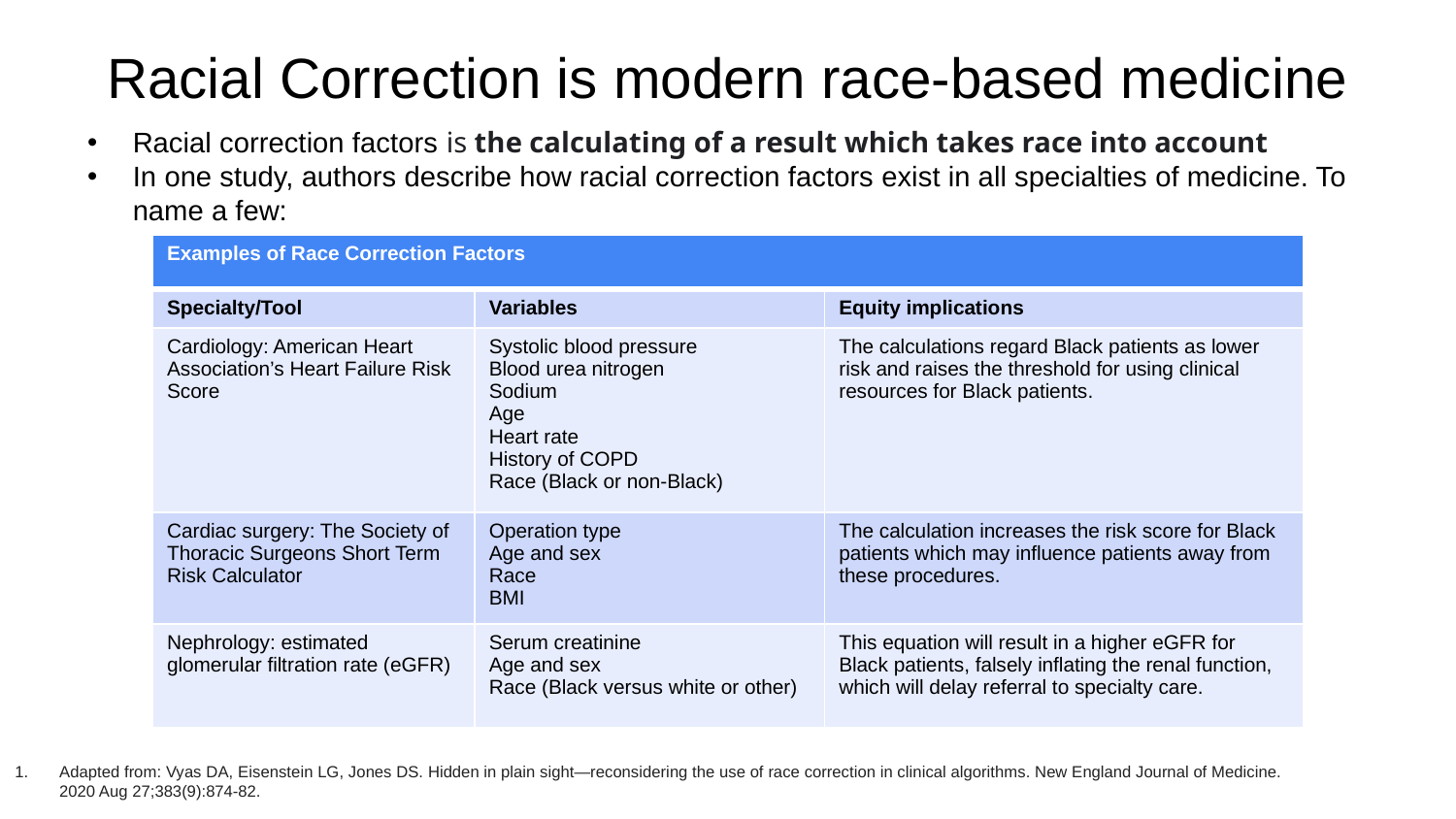

# Racial Correction is modern race-based medicine
Racial correction factors is the calculating of a result which takes race into account
In one study, authors describe how racial correction factors exist in all specialties of medicine. To name a few:
| Examples of Race Correction Factors | | |
| --- | --- | --- |
| Specialty/Tool | Variables | Equity implications |
| Cardiology: American Heart Association’s Heart Failure Risk Score | Systolic blood pressure Blood urea nitrogen Sodium Age Heart rate History of COPD Race (Black or non-Black) | The calculations regard Black patients as lower risk and raises the threshold for using clinical resources for Black patients. |
| Cardiac surgery: The Society of Thoracic Surgeons Short Term Risk Calculator | Operation type Age and sex Race BMI | The calculation increases the risk score for Black patients which may influence patients away from these procedures. |
| Nephrology: estimated glomerular filtration rate (eGFR) | Serum creatinine Age and sex Race (Black versus white or other) | This equation will result in a higher eGFR for Black patients, falsely inflating the renal function, which will delay referral to specialty care. |
Adapted from: Vyas DA, Eisenstein LG, Jones DS. Hidden in plain sight—reconsidering the use of race correction in clinical algorithms. New England Journal of Medicine. 2020 Aug 27;383(9):874-82.

## Slide 31
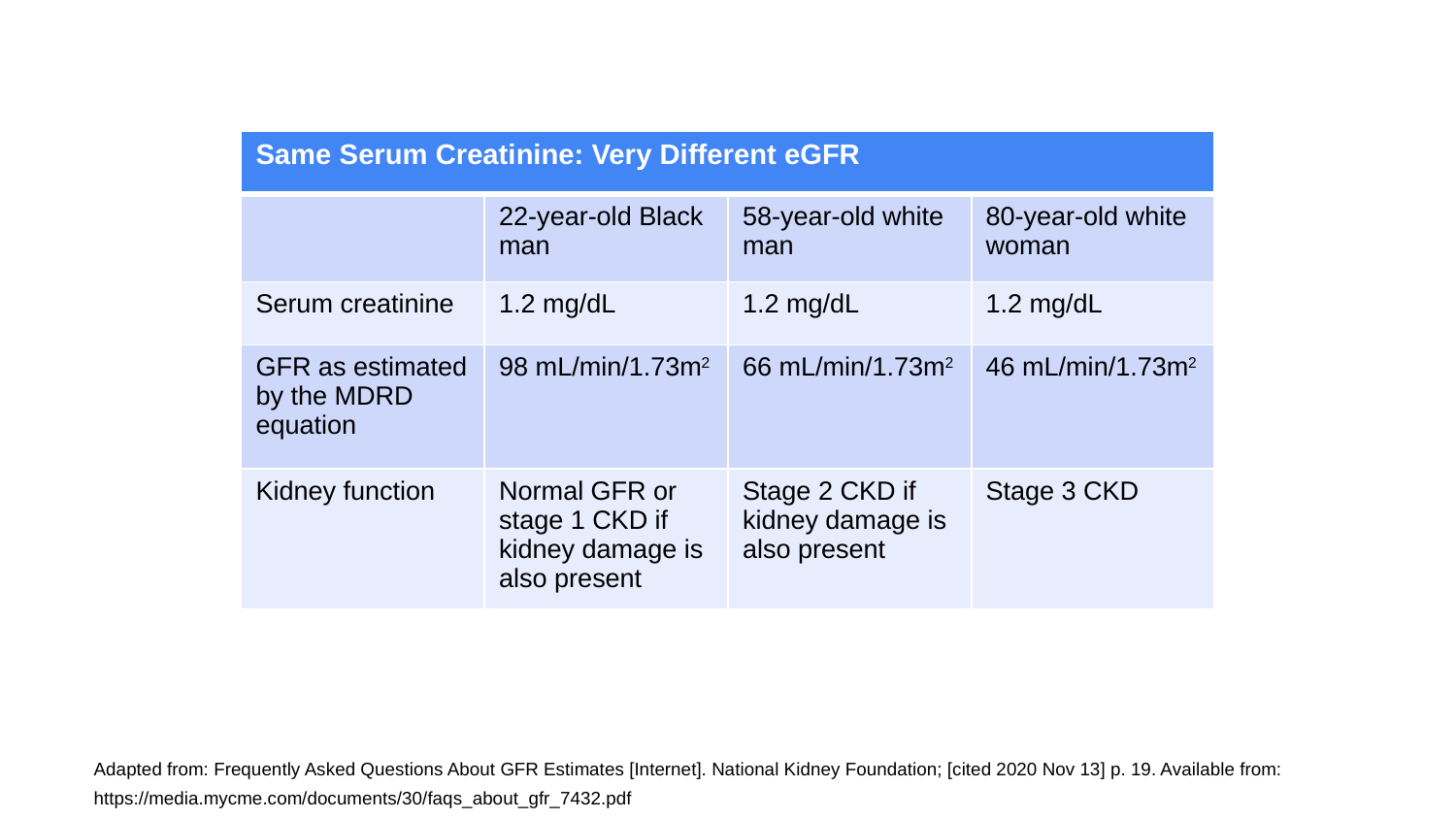

| Same Serum Creatinine: Very Different eGFR | | | |
| --- | --- | --- | --- |
| | 22-year-old Black man | 58-year-old white man | 80-year-old white woman |
| Serum creatinine | 1.2 mg/dL | 1.2 mg/dL | 1.2 mg/dL |
| GFR as estimated by the MDRD equation | 98 mL/min/1.73m2 | 66 mL/min/1.73m2 | 46 mL/min/1.73m2 |
| Kidney function | Normal GFR or stage 1 CKD if kidney damage is also present | Stage 2 CKD if kidney damage is also present | Stage 3 CKD |
Adapted from: Frequently Asked Questions About GFR Estimates [Internet]. National Kidney Foundation; [cited 2020 Nov 13] p. 19. Available from: https://media.mycme.com/documents/30/faqs_about_gfr_7432.pdf

## Slide 32
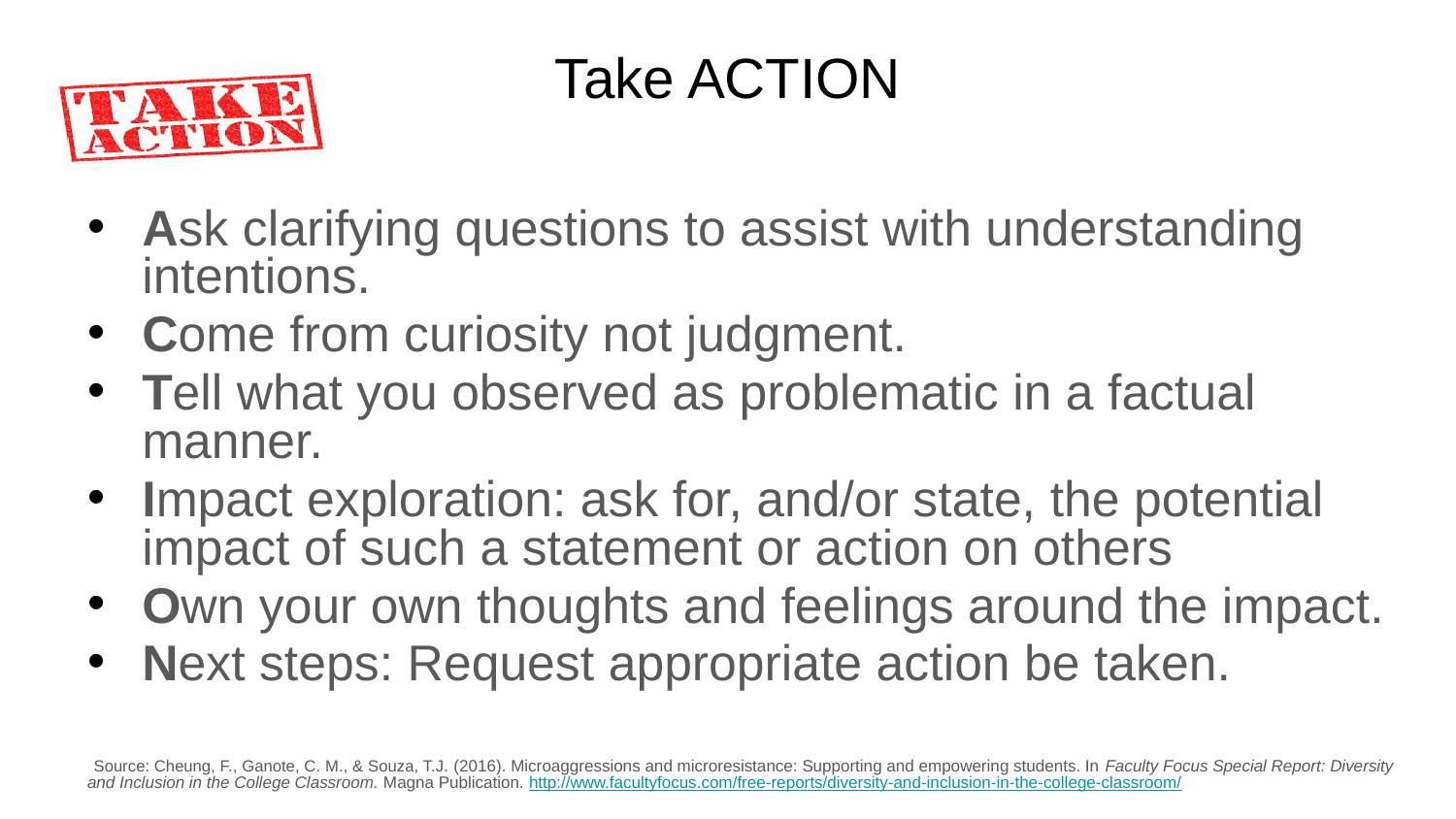

# Take ACTION
Ask clarifying questions to assist with understanding intentions.
Come from curiosity not judgment.
Tell what you observed as problematic in a factual manner.
Impact exploration: ask for, and/or state, the potential impact of such a statement or action on others
Own your own thoughts and feelings around the impact.
Next steps: Request appropriate action be taken.
 Source: Cheung, F., Ganote, C. M., & Souza, T.J. (2016). Microaggressions and microresistance: Supporting and empowering students. In Faculty Focus Special Report: Diversity and Inclusion in the College Classroom. Magna Publication. http://www.facultyfocus.com/free-reports/diversity-and-inclusion-in-the-college-classroom/

## Slide 33
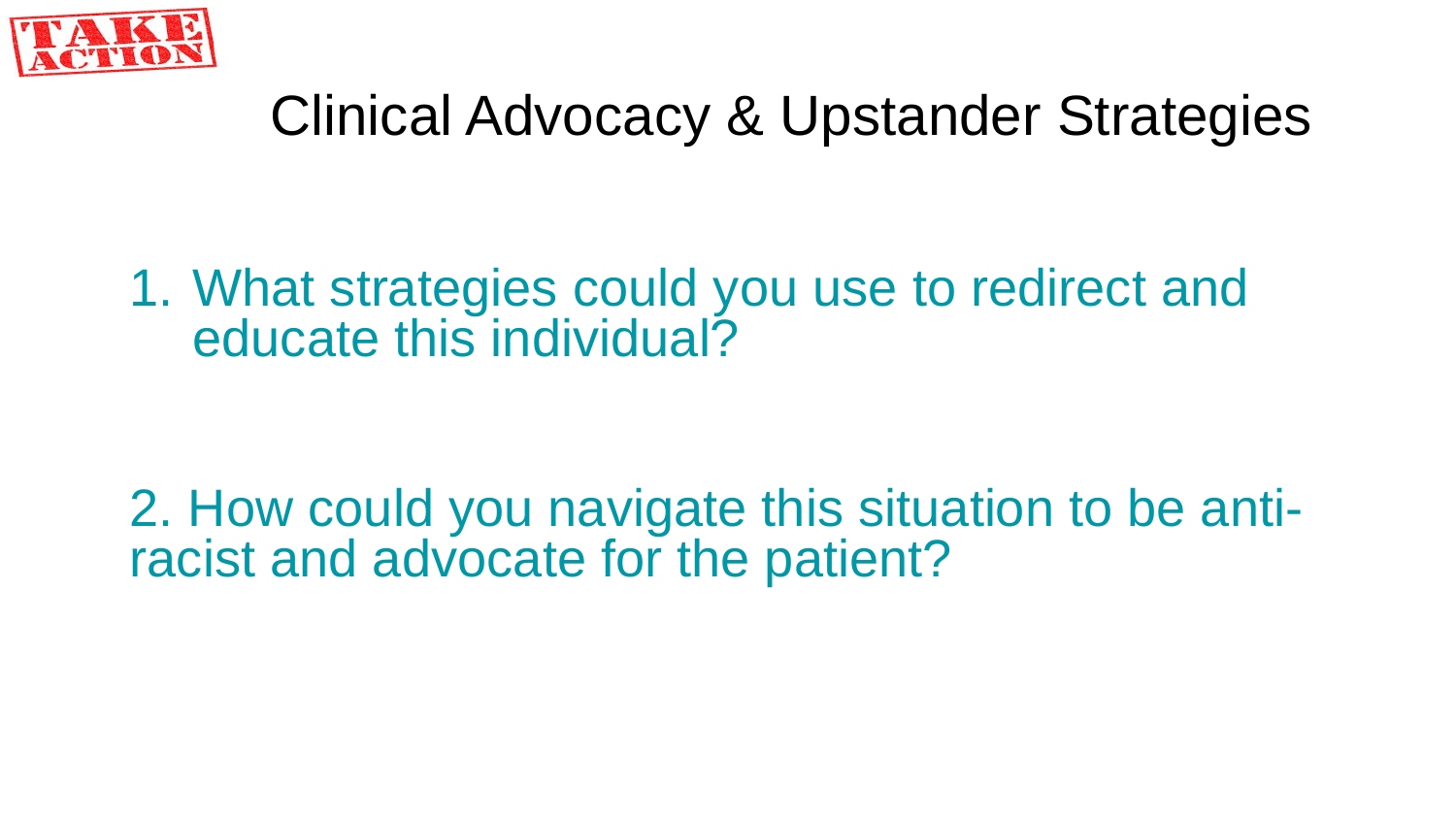

# Clinical Advocacy & Upstander Strategies
What strategies could you use to redirect and educate this individual?
2. How could you navigate this situation to be anti-racist and advocate for the patient?
