# Supplementary material for: Core Competencies of an Anti-racist Physician: Elective Course for Undergraduate Medical Students
Source: MedEdPORTAL. 2024 May 14;20:11395. doi: 10.15766/mep_2374-8265.11395 (PMC11219086; doi:10.15766/mep_2374-8265.11395)
Supplement: Supplementary file 1 — Disorienting Dilemmas.docxFacilitator Guidelines.docxPrework Module.docxOpening Slides.pptxFacilitator Slides.pptxClosing Remarks Slides.pptxExit Ticket.docxPre- and Postassessment.docx [file mep_2374-8265.11395-s001.zip › F. Closing Remarks Slides.pptx]

## Slide 1
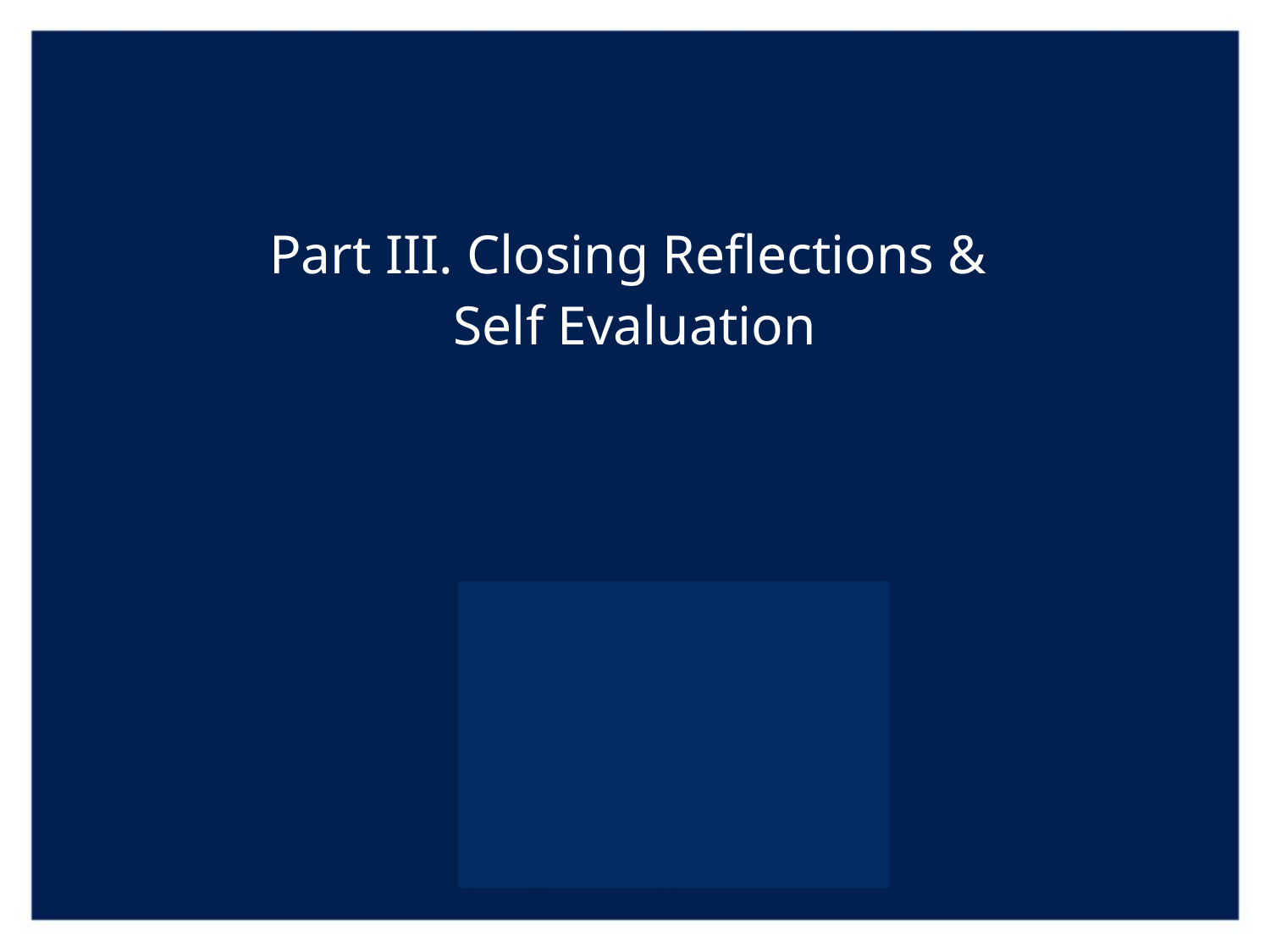

# Part III. Closing Reflections & Self Evaluation

## Slide 2
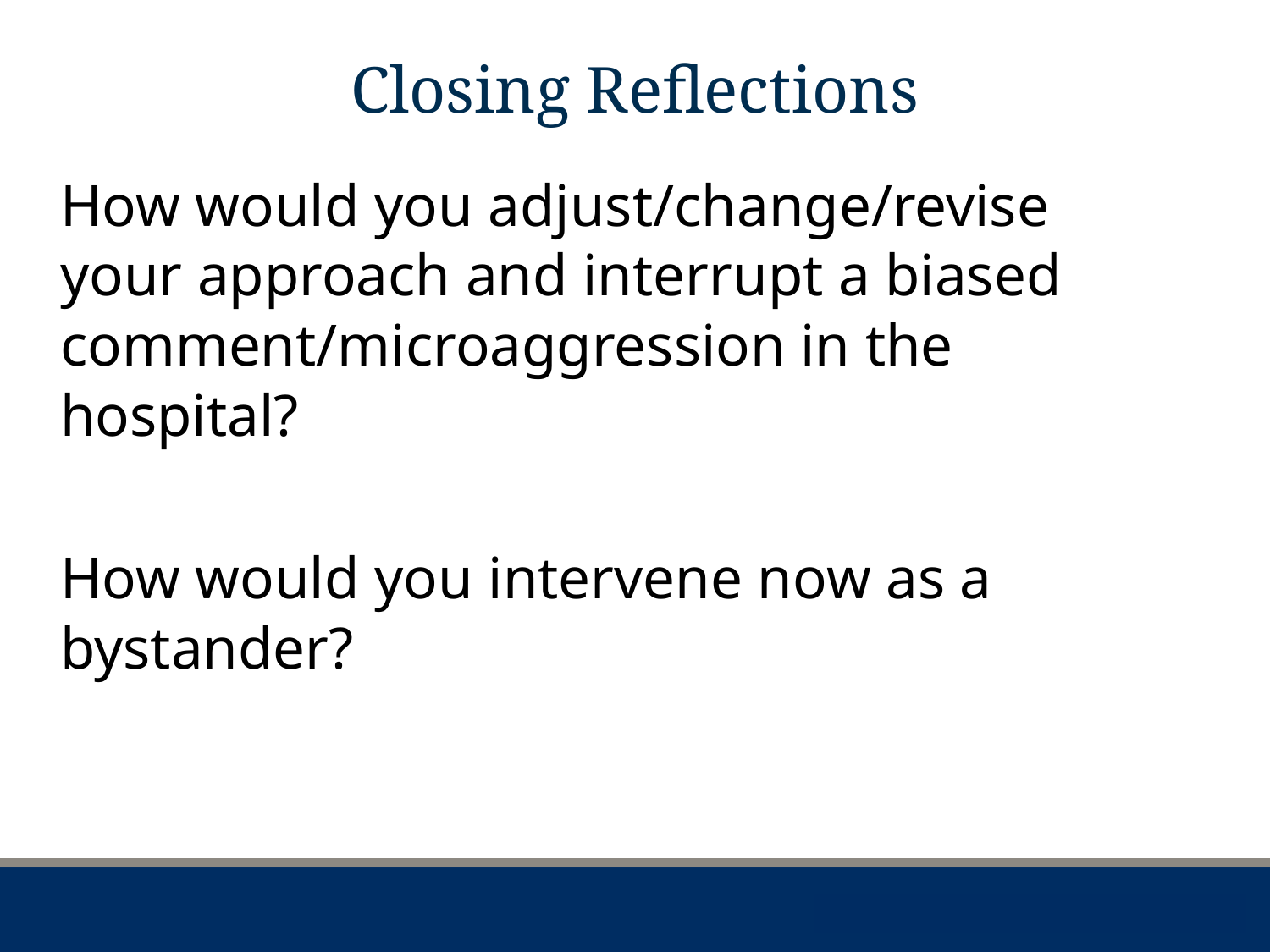

# Closing Reflections
How would you adjust/change/revise your approach and interrupt a biased comment/microaggression in the hospital?
How would you intervene now as a bystander?

## Slide 3
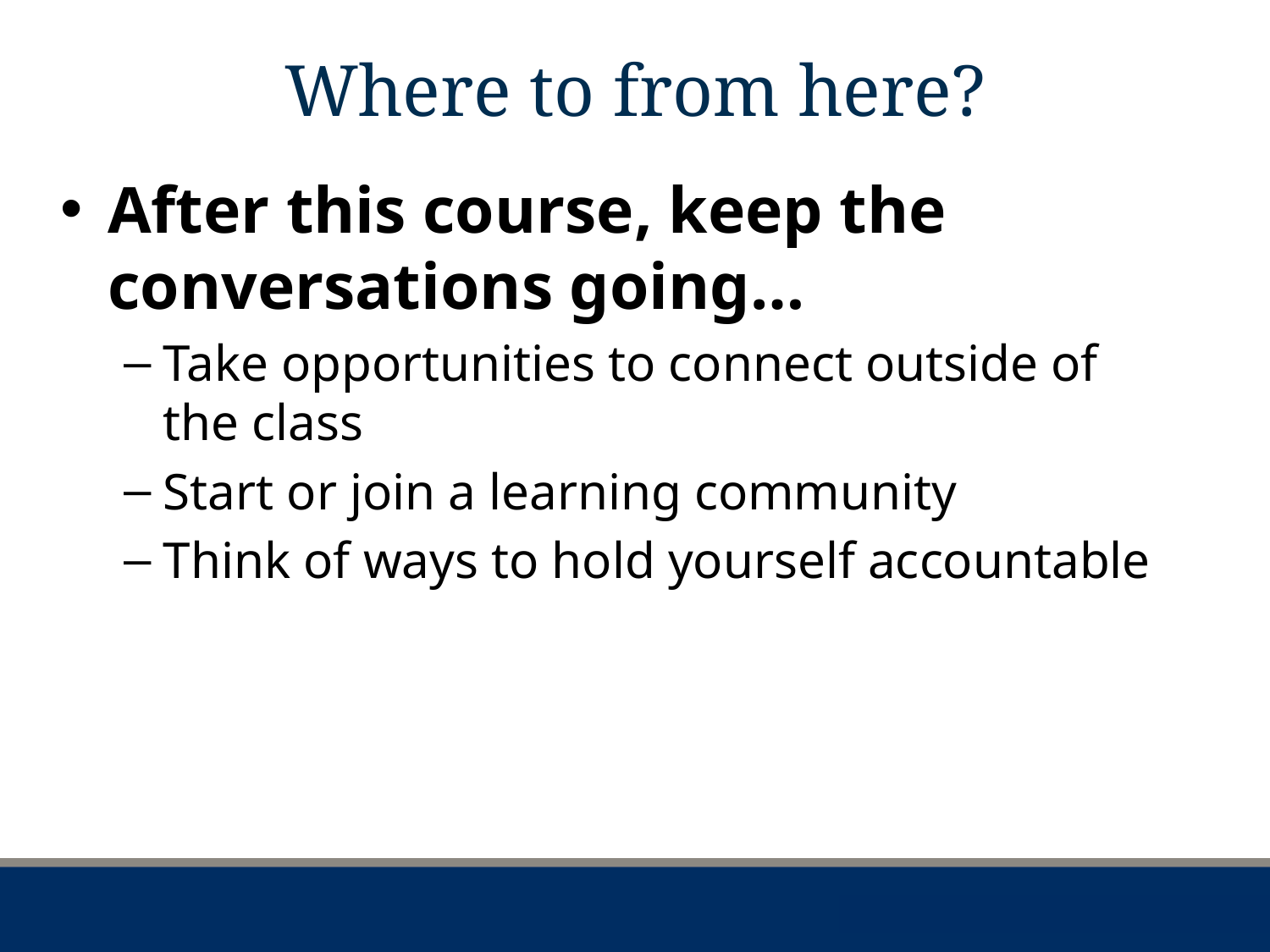

# Where to from here?
After this course, keep the conversations going…
Take opportunities to connect outside of the class
Start or join a learning community
Think of ways to hold yourself accountable

## Slide 4
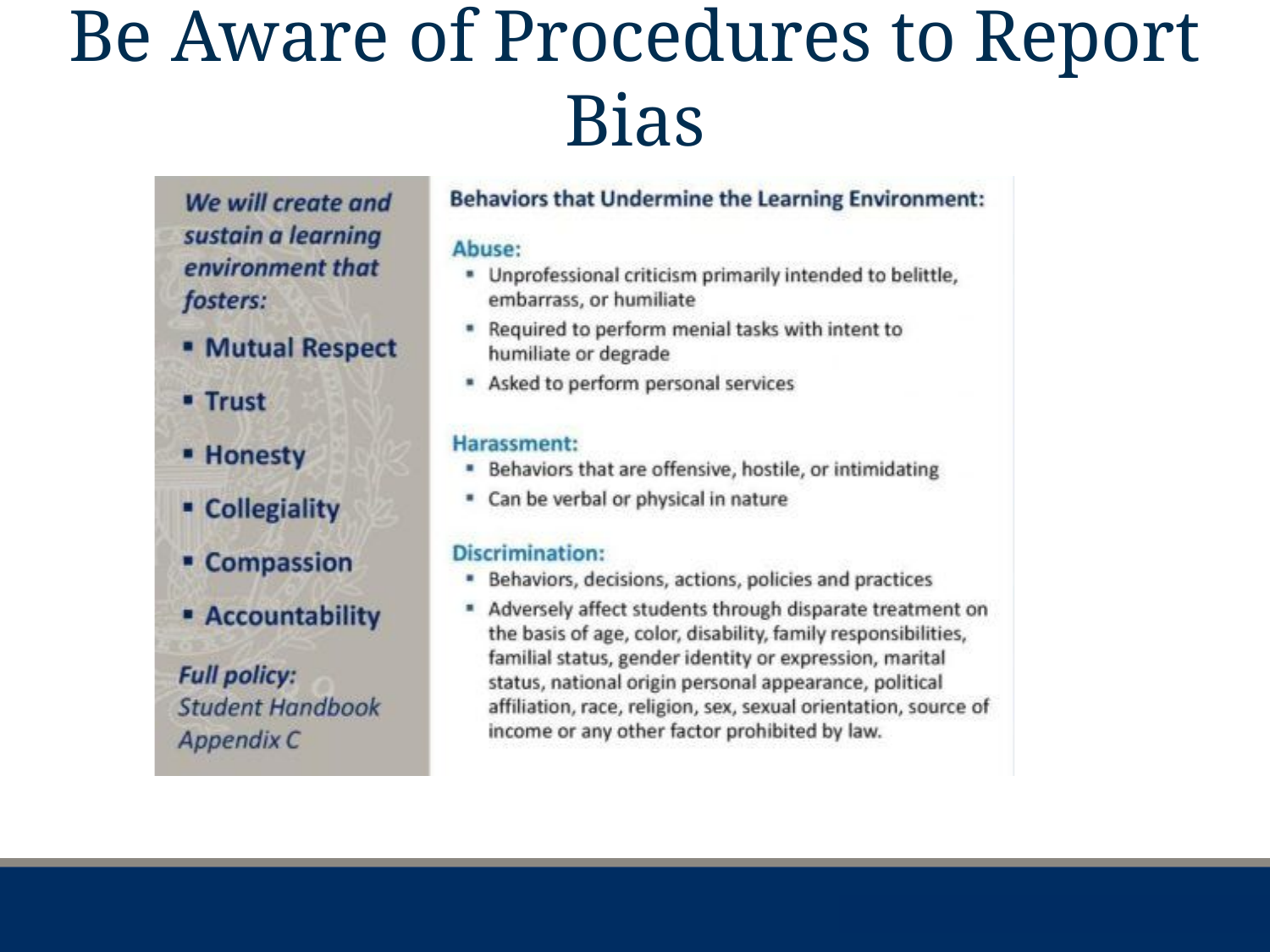

# Be Aware of Procedures to Report Bias

## Slide 5
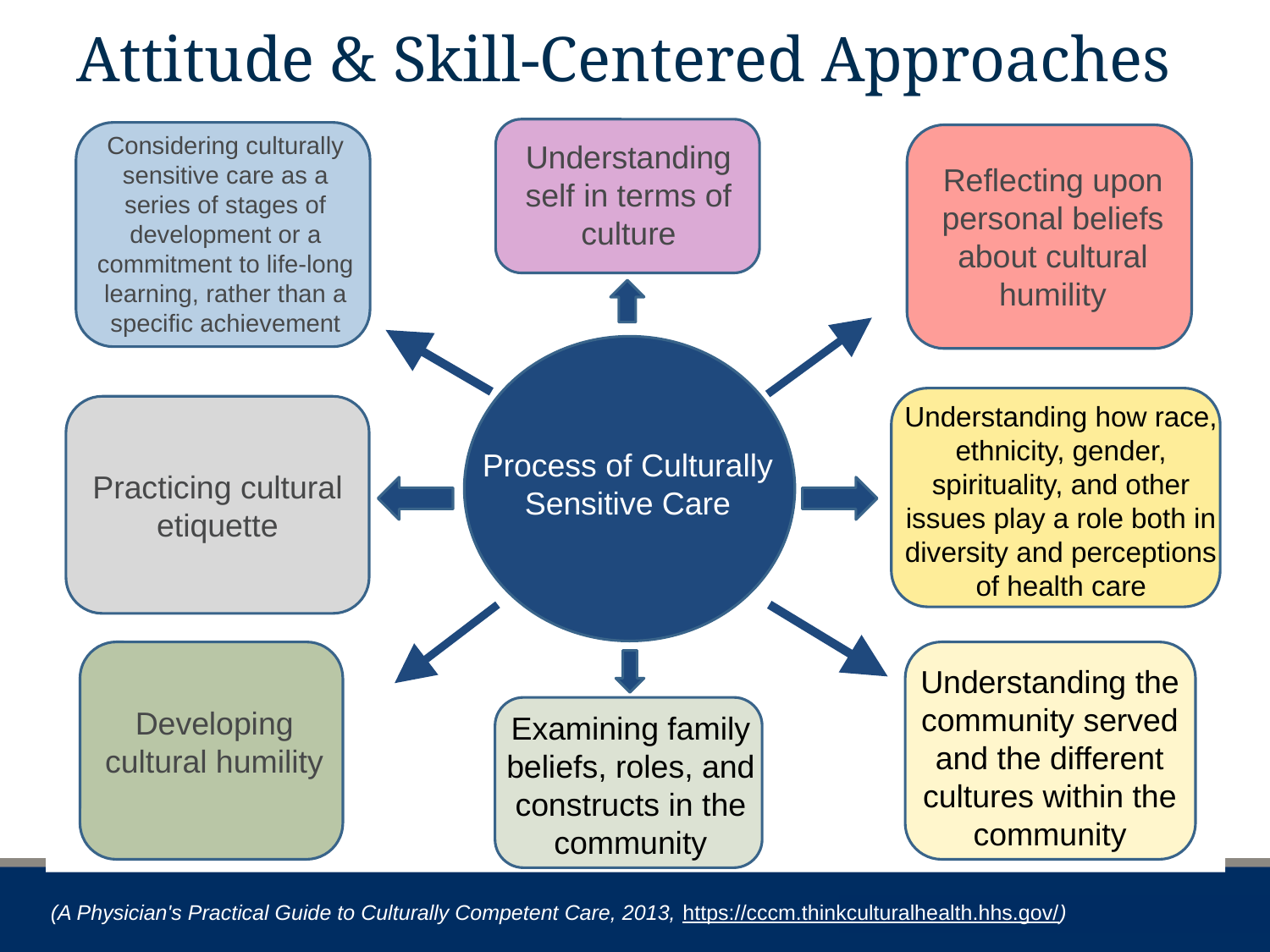

# Attitude & Skill-Centered Approaches
Considering culturally sensitive care as a series of stages of development or a commitment to life-long learning, rather than a specific achievement
Understanding self in terms of culture
Reflecting upon personal beliefs about cultural humility
Understanding how race, ethnicity, gender, spirituality, and other issues play a role both in diversity and perceptions of health care
Practicing cultural etiquette
Process of Culturally Sensitive Care
Understanding the community served and the different cultures within the community
Developing cultural humility
Examining family beliefs, roles, and constructs in the community
‹#›
(A Physician's Practical Guide to Culturally Competent Care, 2013, https://cccm.thinkculturalhealth.hhs.gov/)

## Slide 6
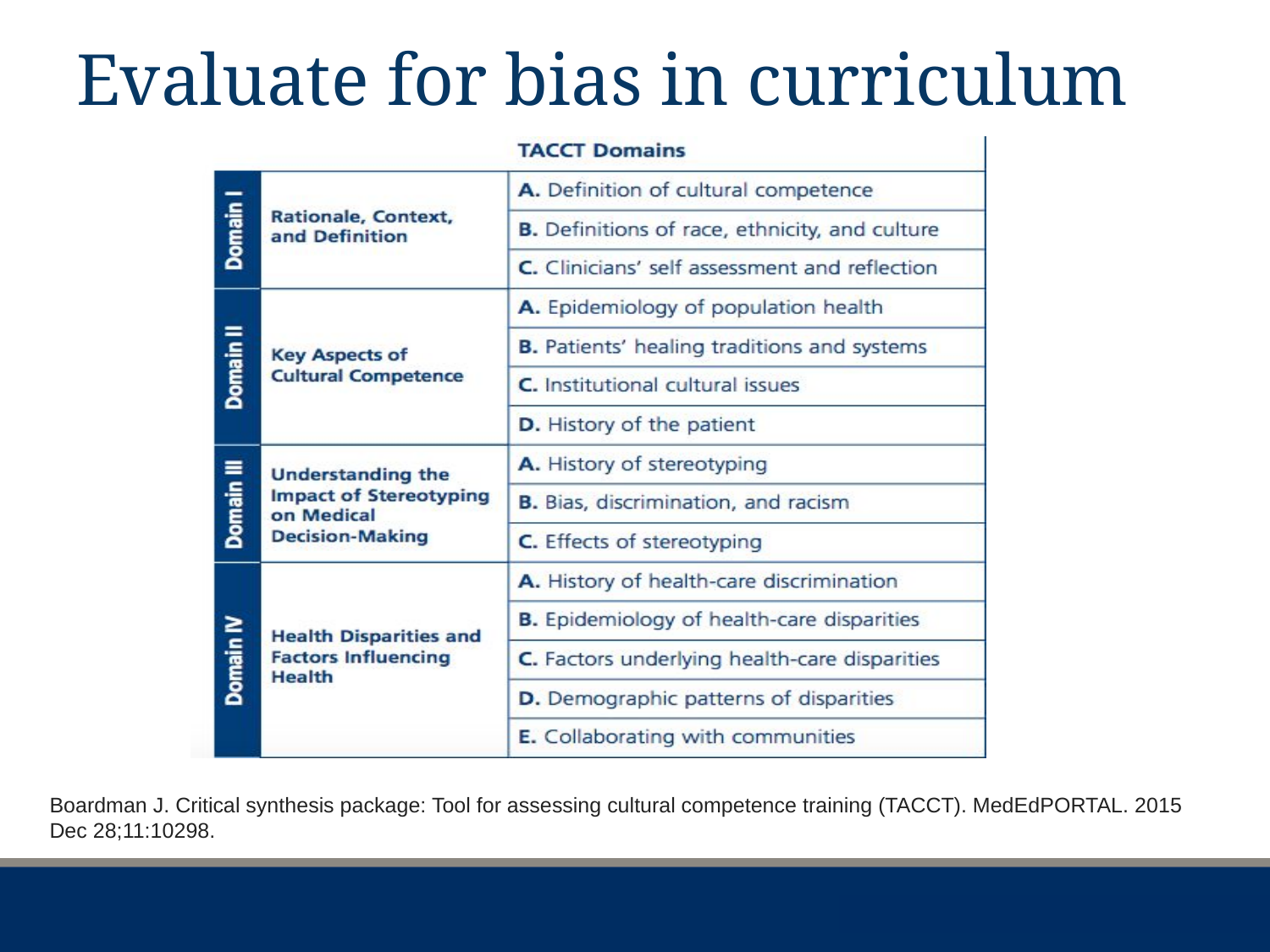

Evaluate for bias in curriculum
Boardman J. Critical synthesis package: Tool for assessing cultural competence training (TACCT). MedEdPORTAL. 2015 Dec 28;11:10298.
‹#›

## Slide 7
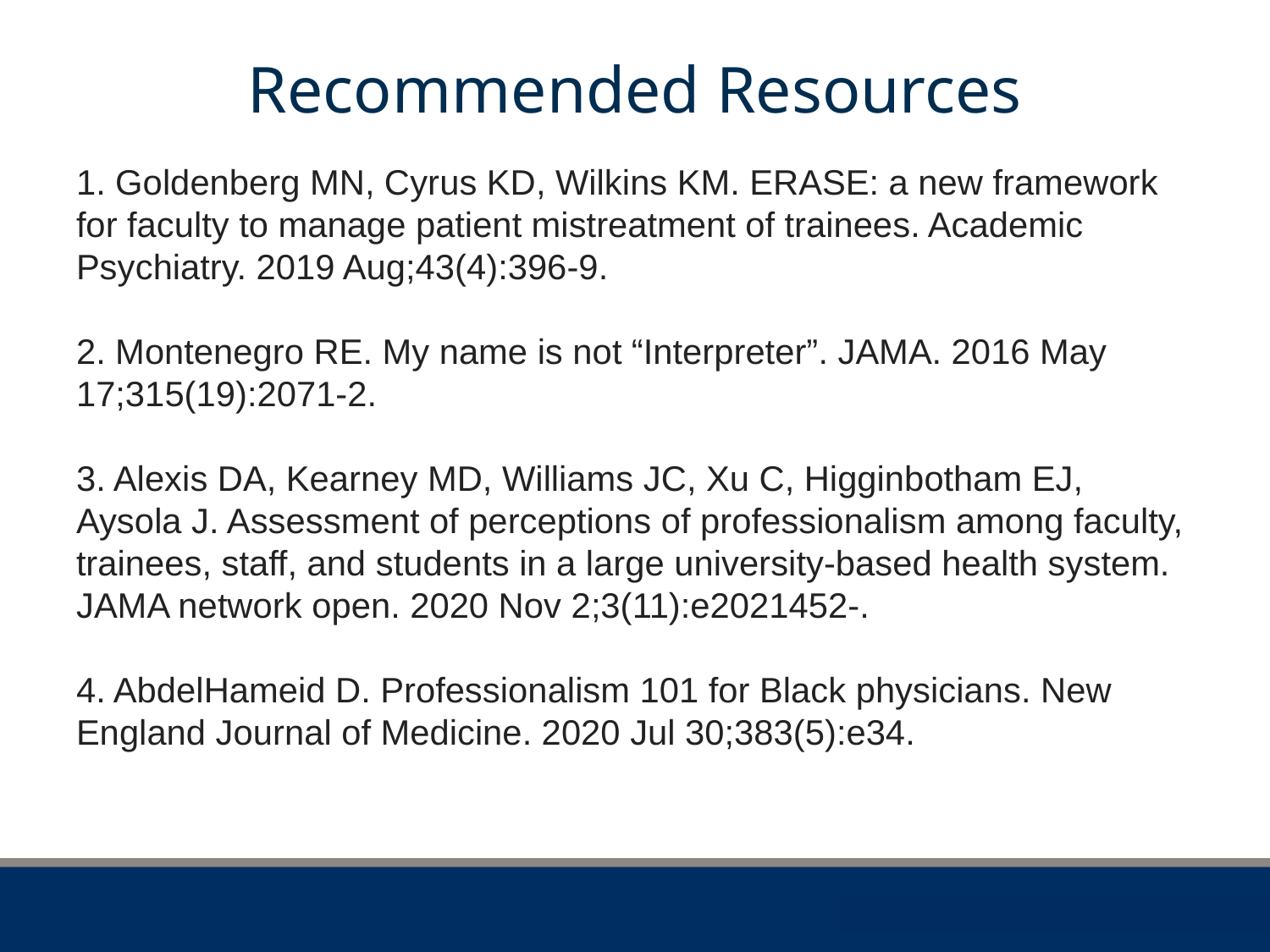

# Recommended Resources
1. Goldenberg MN, Cyrus KD, Wilkins KM. ERASE: a new framework for faculty to manage patient mistreatment of trainees. Academic Psychiatry. 2019 Aug;43(4):396-9.
2. Montenegro RE. My name is not “Interpreter”. JAMA. 2016 May 17;315(19):2071-2.
3. Alexis DA, Kearney MD, Williams JC, Xu C, Higginbotham EJ, Aysola J. Assessment of perceptions of professionalism among faculty, trainees, staff, and students in a large university-based health system. JAMA network open. 2020 Nov 2;3(11):e2021452-.
4. AbdelHameid D. Professionalism 101 for Black physicians. New England Journal of Medicine. 2020 Jul 30;383(5):e34.

## Slide 8
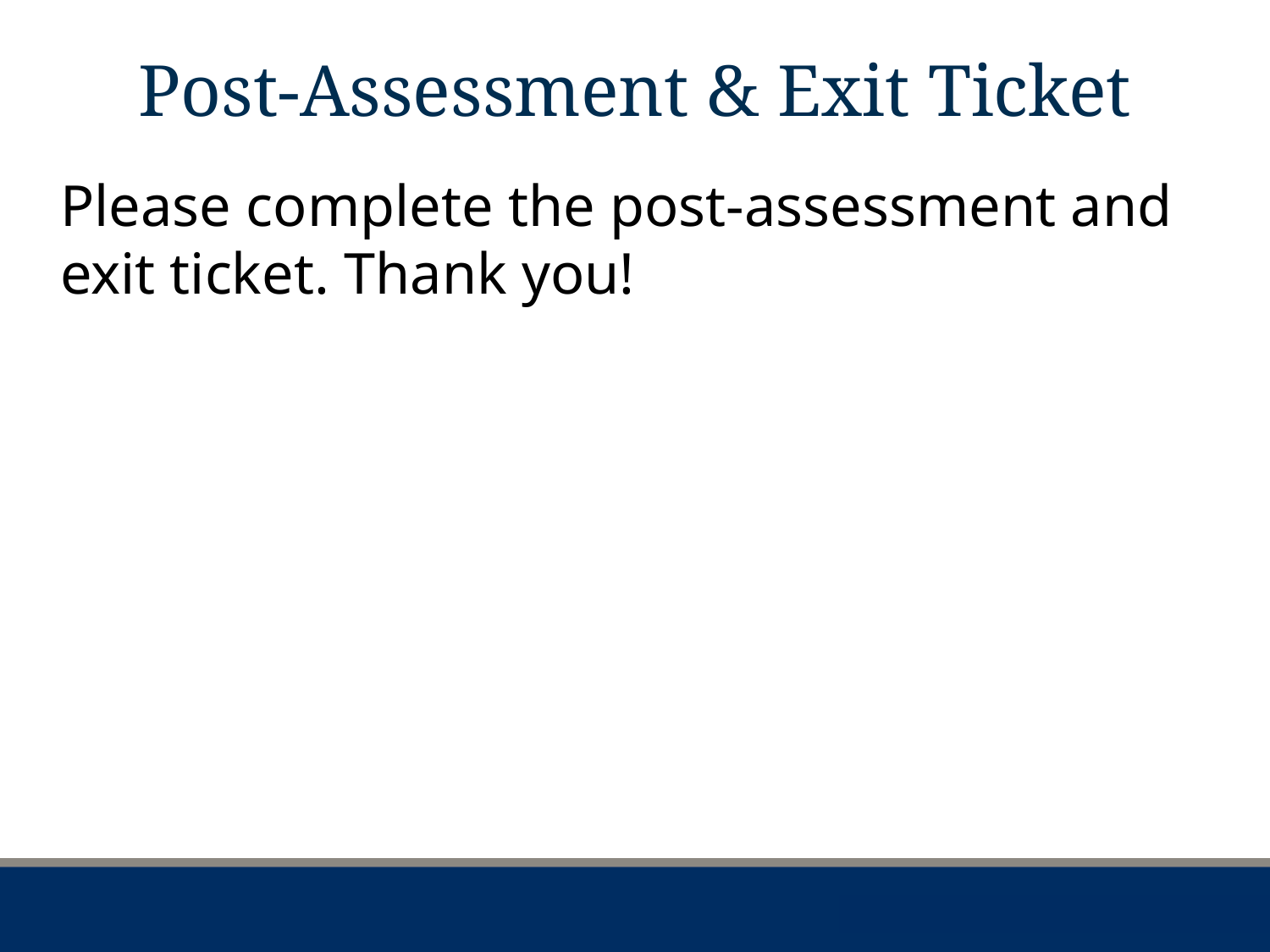

# Post-Assessment & Exit Ticket
Please complete the post-assessment and exit ticket. Thank you!
