# Supplementary material for: Core Competencies of an Anti-racist Physician: Elective Course for Undergraduate Medical Students
Source: MedEdPORTAL. 2024 May 14;20:11395. doi: 10.15766/mep_2374-8265.11395 (PMC11219086; doi:10.15766/mep_2374-8265.11395)
Supplement: Supplementary file 1 — Disorienting Dilemmas.docxFacilitator Guidelines.docxPrework Module.docxOpening Slides.pptxFacilitator Slides.pptxClosing Remarks Slides.pptxExit Ticket.docxPre- and Postassessment.docx [file mep_2374-8265.11395-s001.zip › G. Exit Ticket.docx]

Anti-Racist Physician Course - Exit Ticket

Anti-Racist Physician Course - Exit Ticket

After today's session, please take 5 minutes to complete an anonymous exit ticket that will help improve the session. Thanks!


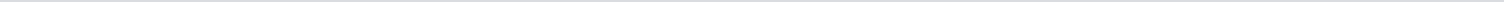


* Required

1. Rate the effectiveness of the session today. *

***Mark only one oval.***

1 2 3 4 5


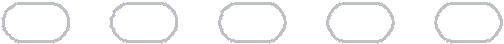

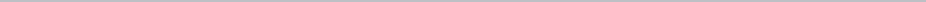


Not effective Highly effective


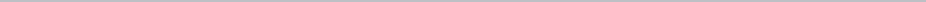


1. What did the session help you to understand better?


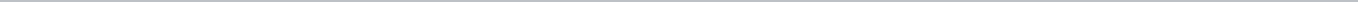

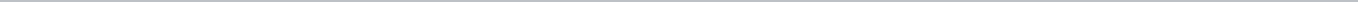

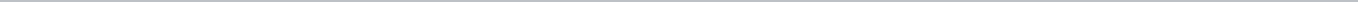

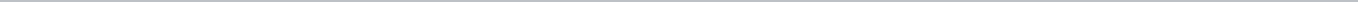

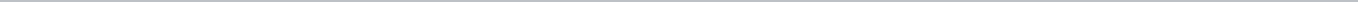


1. How could the session be improved?


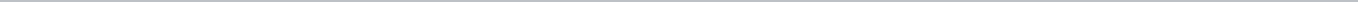

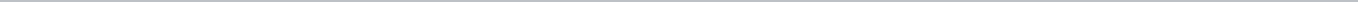

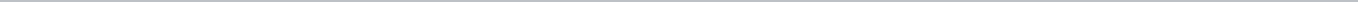

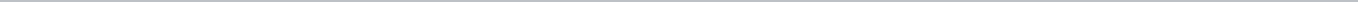

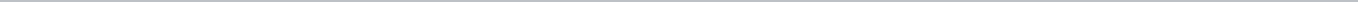


1. What is one thing you can walk away with from today’s session that will have an immediate impact on your work?


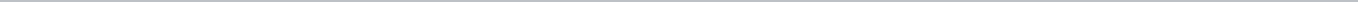

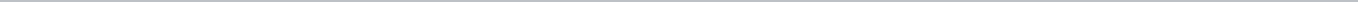

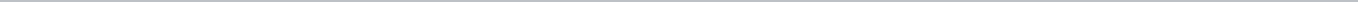

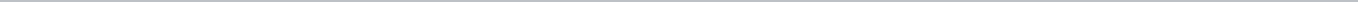

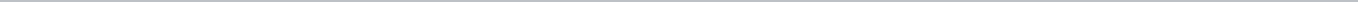


1. What is the most pressing question you still have, after today’s session?


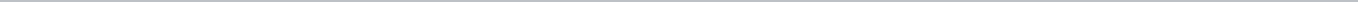

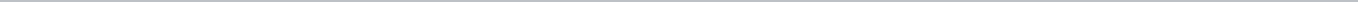

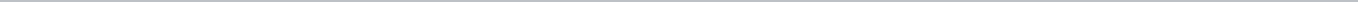

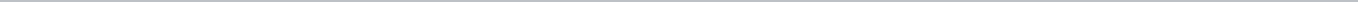

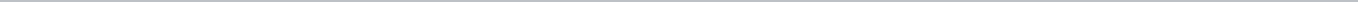


1. Any other comments or concerns:


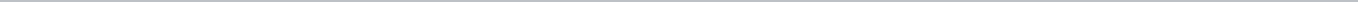

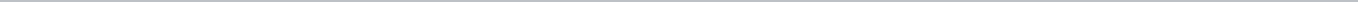

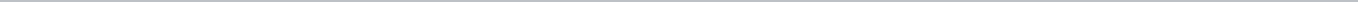

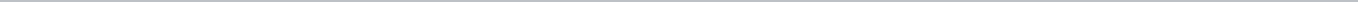

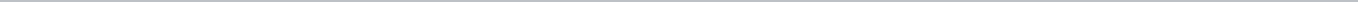


2/2
