# Supplementary material for: Core Competencies of an Anti-racist Physician: Elective Course for Undergraduate Medical Students
Source: MedEdPORTAL. 2024 May 14;20:11395. doi: 10.15766/mep_2374-8265.11395 (PMC11219086; doi:10.15766/mep_2374-8265.11395)
Supplement: Supplementary file 1 — Disorienting Dilemmas.docxFacilitator Guidelines.docxPrework Module.docxOpening Slides.pptxFacilitator Slides.pptxClosing Remarks Slides.pptxExit Ticket.docxPre- and Postassessment.docx [file mep_2374-8265.11395-s001.zip › H. Pre- and Postassessment.docx]

**Core Competencies for the Anti-Racist Physician – pre-assessment**

Consent:

Thanks for your interest in the course "Core Competencies of the Anti-Racist Physician". We hope you learn important knowledge, skills, and attitudes from this course. If you have not already, please complete the online learning module before you complete this pre-assessment.

You are invited to take part in the following pre-assessment to better assess learning outcomes. Your participation will require approximately 10 minutes and is completed online at your computer or mobile device.

There are no known risks or discomforts associated with this assessment. Taking part in this assessment is completely voluntary. If you choose to participate you can exit at any time without adversely affecting your grade or relationship with anyone. Your responses will be kept strictly confidential, and de-identified digital data will be stored in secure computer files.

If any report of this assessment is made available for research purposes, we will not include your name or any other individual information by which you could be identified. If you have questions or want a copy or summary of the assessment results, you can contact Dr. Williams (jcorey.a.williams@medstar.net). Please feel free to print a copy of this consent page to keep for your records.

Clicking the next arrow ">>" below indicates that you are 18 years of age or older and indicates your consent to participate in this assessment.

1. Patients with minoritized racial identities are routinely treated differently in the hospital, especially Black patients.

|  | 1 | 2 | 3 | 4 | 5 |
| --- | --- | --- | --- | --- | --- |

| (1)Strongly Disagree (2) Disagree (3) Neutral (4)Agree (5)Strongly Agree | 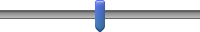 |
| --- | --- |

1. I know where to go to report an incident of racism that occurred in a clinical setting.

| (1)Strongly Disagree (2) Disagree (3) Neutral (4)Agree (5)Strongly Agree | 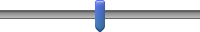 |
| --- | --- |

1. Anti-Black racism is a problem in U.S. healthcare systems.

| (1)Strongly Disagree (2) Disagree (3) Neutral (4)Agree (5)Strongly Agree | 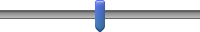 |
| --- | --- |

1. The racialized comments and narratives that emerge in clinical situations may reinforce misconceptions about racial groups and can lead to the biased treatment of patients.

| (1)Strongly Disagree (2) Disagree (3) Neutral (4)Agree (5)Strongly Agree | 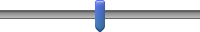 |
| --- | --- |

1. Black patients are sometimes associated with harmful stereotypes and negative value judgments.

| (1)Strongly Disagree (2) Disagree (3) Neutral (4)Agree (5)Strongly Agree | 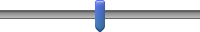 |
| --- | --- |

1. Evidence shows that Black patients routinely receive less patient-centered care and poor communication from providers.

| (1)Strongly Disagree (2) Disagree (3) Neutral (4)Agree (5)Strongly Agree | 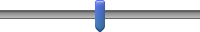 |
| --- | --- |

1. Structurally racist policies and practices from the past continue to impact patients today.

| (1)Strongly Disagree (2) Disagree (3) Neutral (4)Agree (5)Strongly Agree | 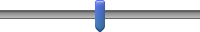 |
| --- | --- |

1. I understand some of the relevant historical roots that shape structural racism in U.S. healthcare.

| (1)Strongly Disagree (2) Disagree (3) Neutral (4)Agree (5)Strongly Agree | 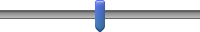 |
| --- | --- |

1. The legacy of scientific racism has shaped modern medicine in ways that have contributed to disparities in care.

| (1)Strongly Disagree (2) Disagree (3) Neutral (4)Agree (5)Strongly Agree | 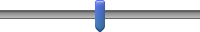 |
| --- | --- |

1. I have a good sense of how anti-Black racism can manifest in a clinical setting.

| (1)Strongly Disagree (2) Disagree (3) Neutral (4)Agree (5)Strongly Agree | 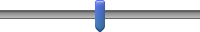 |
| --- | --- |

1. I feel empowered to interrupt racist comments in a clinical setting.

| (1)Strongly Disagree (2) Disagree (3) Neutral (4)Agree (5)Strongly Agree | 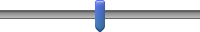 |
| --- | --- |

1. It is my responsibility to interrupt and redirect racist speech and behavior in clinical settings.

| (1)Strongly Disagree (2) Disagree (3) Neutral (4)Agree (5)Strongly Agree | 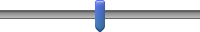 |
| --- | --- |

1. I understand the ways that race is falsely biologized in a clinical context.

| (1)Strongly Disagree (2) Disagree (3) Neutral (4)Agree (5)Strongly Agree | 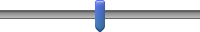 |
| --- | --- |

1. I understand strategies to interrupt or redirect racist narratives and comments in a clinical setting.

| (1)Strongly Disagree (2) Disagree (3) Neutral (4)Agree (5)Strongly Agree | 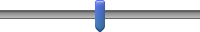 |
| --- | --- |

1. I understand ways to practice self-care when encountering stressful situations in a clinical setting.

| (1)Strongly Disagree (2) Disagree (3) Neutral (4)Agree (5)Strongly Agree | 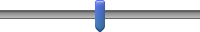 |
| --- | --- |

1. In the space below, set a racial equity goal for your personal and/or professional life. For example: "I am planning to read more books by Black authors." "I will interrupt or redirect racist hate speech in my personal or professional life." "I will take the IAT to better understand my bias."
